# Rationally designed dual-plasmonic gold nanorod@cuprous selenide hybrid heterostructures by regioselective overgrowth for *in vivo* photothermal tumor ablation in the second near-infrared biowindow

**DOI:** 10.7150/thno.51287

**Published:** 2020-09-19

**Authors:** Beibei Shan, Haitao Wang, Linhu Li, Guangzhi Zhou, Yu Wen, Mingyang Chen, Ming Li

**Affiliations:** School of Materials Science and Engineering, Central South University, Changsha, Hunan 410083, China.

**Keywords:** plasmonic materials, *in vivo* photothermal therapy, NIR-II light, site-selective deposition, metal-semiconductor heterostructures

## Abstract

NIR-II plasmonic materials offer multiple functionalities for *in vivo* biomedical applications, such as photothermal tumor ablation, surface-enhanced Raman scattering biosensing, photoacoustic imaging, and drug carriers. However, integration of noble metals and plasmonic semiconductors is greatly challenging because of the large lattice-mismatch. This study reports the regioselective overgrowth of Cu_2-x_Se on gold nanorods (GNRs) for preparation of dual-plasmonic GNR@Cu_2-x_Se hybrid heterostructures with tunable NIR-II plasmon resonance absorption for *in vivo* photothermal tumor ablation.

**Methods:** The regioselective deposition of amorphous Se and its subsequent conversion into Cu_2-x_Se on the GNRs are performed by altering capping agents to produce the GNR@Cu_2-x_Se heterostructures of various morphologies. Their photothermal performances for NIR-II photothermal tumor ablation are evaluated both *in vitro* and *in vivo*.

**Results:** We find that the lateral one- and two-side deposition, conformal core-shell coating and island growth of Cu_2-x_Se on the GNRs can be achieved using different capping agents. The Cu_2-x_Se domain size in these hybrids can be effectively adjusted by the SeO_2_ concentration, thereby tuning the NIR-II plasmon bands. A photothermal conversion efficiency up to 58-85% and superior photostability of these dual-plasmonic hybrids can be achieved under the NIR-II laser. Results also show that the photothermal conversion efficiency is dependent on the proportion of optical absorption converted into heat; however, the temperature rise is tightly related to the concentration of their constituents. The excellent NIR-II photothermal effect is further verified in the following *in vitro* and *in vivo* experiments.

**Conclusions:** This study achieves one-side or two-side deposition, conformal core-shell coating, and island deposition of Cu_2-x_Se on GNRs for GNR@Cu_2-x_Se heterostructures with NIR-II plasmonic absorption, and further demonstrates their excellent NIR-II photothermal tumor ablation *in vivo*. This study provides a promising strategy for the rational design of NIR-II dual-plasmonic heterostructures and highlights their therapeutic *in vivo* potential.

## Introduction

Multifunctional plasmonic materials operating in the second near-infrared biowindow (NIR-II, 1000-1700 nm) have attracted considerable attention because they afford tremendous capability for *in vivo* biomedical applications 1-5. The intrinsic advantages of deep tissue penetration, high spatial resolution and high maximum permissible laser exposure to tissues of the NIR-II light make NIR-II plasmonic materials outperform those with plasmonic properties in the visible (400-700 nm) and NIR-I (700-900 nm) regions 6-12. Specifically, these NIR-II plasmonic materials could be exploited in a living system for *in vivo* visualization of deep tumors and implementation of the light-induced tumor therapy through optical modalities including surface-enhanced Raman scattering (SERS), photoacoustic imaging, photothermal and photodynamic therapy (PTT and PDT), and light-triggered chemotherapy. The key to implement these *in vivo* optical diagnostics and therapy lies in the NIR-II plasmonic materials. Plasmonic noble metals (*i.e.*, gold, silver) display size-, composition- and shape-dependent optical properties tunable in the wavelength range from the visible to NIR regions 13-19, while cuprous chalcogenides (Cu_2-x_E, E=S, Se, Te, 0<x<1) are a new set of plasmonic materials of plasmon absorption in the NIR spectral region deriving from the collective oscillation of holes 20-23. Integration of plasmonic noble metals and Cu_2-x_E into a single entity has sparked a wide-ranging enthusiasm for producing dual-plasmonic hybrids for enhanced NIR-II biomedical applications due to the synergistic coupling effect. The integration of two intrinsically dissimilar plasmonic building blocks may synergistically enhance the physical and chemical properties beyond the sum of their constituents and generate new intriguing properties that may not be achievable by their single component materials.

Previous studies chiefly utilized isotropic gold nanoparticles (GNPs) for preparing dual-plasmonic core-shell GNP@Cu_2-x_E structures in which, however, the low spectral overlap of the plasmon bands of the spherical (isotropic) GNP and Cu_2-x_E domains restricts their plasmonic coupling interactions 24-26. Recently, gold-on-gold homometallic hybrids were obtained with the controllable overgrowth of either spherical or branched gold domains on the GNR surface through engineering the interface energy and growth kinetics 27. Compared with isotropic GNPs, gold nanorods (GNRs) have anisotropic morphology and exhibit two plasmon bands called transverse and longitudinal plasmon bands; an outstanding advantage of GNRs is that their longitudinal plasmon band can be pushed into the NIR region by simply varying the aspect ratio (length/width) 28-31. This implies that there may exist a strong coupling interaction between the NIR plasmon bands of GNRs and Cu_2-x_E since they have much larger spectral overlap with respect to the spherical counterparts. The spatial arrangement of the component domains in a hybrid is of paramount importance for the final performance of a hybrid as well 32,33. However, the large lattice-mismatch between the GNRs and Cu_2-x_E is a severe obstacle for the direct epitaxial growth of the Cu_2-x_Se constituents on the GNRs. Few studies have been performed to create spherical GNP-based concentric or eccentric GNP@Cu_2-x_E core-shell and dimer structures by exploiting different capping agents in various synthetic methods such as the template method and cation exchange method 25,26. In addition, the dual-plasmonic GNR@Cu_2-x_Se core-shell structure consisting of a GNR core and a Cu_2-x_Se shell was also developed using a cation exchange method with the GNR@CdE core-shell hybrids as the starting template 34. However, a disadvantage of the cation exchange for the preparation of GNR@Cu_2-x_E hybrids is that the host Cd^2+^ cannot be completely substituted by Cu^+^ so that the residual Cd^2+^ ions in the resulting hybrids could have a harmful effect on the plasmonic properties and the following biomedical applications because of its toxicity and others; high oxygen and moisture sensitivity also requires the cation exchange implemented in an inert glovebox. The rising demand for the *in vivo* optical biomedical applications has driven the development of high-quality dual-plasmonic GNR@Cu_2-x_E hybrids with optical response in the NIR-II region. Although site-specific overgrowth of a second metal on the faceted GNRs has been extensively studied through the control of either surface energy or regiospecific functionalization 35-39, the anisotropic overgrowth of Cu_2-x_E on the GNRs has yet to be explored. On all accounts, developing an effective synthetic strategy for regiospecific deposition of Cu_2-x_E on GNRs is urgently needed but remains a great challenge for preparation of GNR@Cu_2-x_E hybrids with optimal NIR-II plasmonic properties. In addition, fine control of spatial arrangement of Cu_2-x_E on the GNR surface will not only produce high-quality NIR-II plasmonic materials for high-performance *in vivo* diagnostic and therapeutic applications but also enable the fundamental investigation of the plasmonic coupling in the dual-plasmonic GNR@Cu_2-x_E hybrids.

Herein, we report the regioselective overgrowth of Cu_2-x_Se on the GNRs by controlling the surface chemistry to prepare dual-plasmonic GNR@Cu_2-x_Se heterostructures of tunable plasmonic properties in the NIR-II biowindow for *in vivo* photothermal tumor ablation. The lateral one-side deposition, lateral two-side deposition, conformal core-shell coating and island growth of Cu_2-x_Se on GNRs were realized with cationic hexadecyltrimethylammonium chloride (CTAC), cationic hexadecyltrimethylammonium bromide (CTAB), neutral poly(vinylpyrrolidone) (PVP) and anionic poly(sodium 4-styrenesulfonate) (PSS), and cationic poly(diallyldimethyl ammonium chloride) (PDDA), respectively. Specifically, the Se layer was first selectively deposited onto the GNRs and then converted to the plasmonic Cu_2-x_Se in the presence of various capping agents. The Cu_2-x_Se thickness in all these GNR@Cu_2-x_Se structures can be effectively controlled by varying the starting SeO_2_ concentration, accompanying the tunable NIR-II plasmonic properties. Both experimentation and theoretical calculations confirmed the high photothermal conversion efficiency of these GNR@Cu_2-x_Se structures under either NIR-I (808 nm) or NIR-II (1064 nm) laser. We demonstrated the highly efficient photothermal ablation of tumors at an *in vivo* level with the combination of GNR@Cu_2-x_Se hybrids and NIR-II laser. The present work provides a promising strategy for the regioselective overgrowth of Cu_2-x_Se on anisotropic GNRs and demonstrates their excellent performance for *in vivo* optical biomedical applications with reduced side effects.

## Methods

### Chemicals and materials

Trisodium citrate dihydrate (HOC(COONa)(CH_2_COONa)_2_•2H_2_O, analytical reagent), hydrochloride (HCl, 36.5%), silver nitrate (AgNO_3_, ≥99.8%), sodium borohydride (NaBH_4_, >98%), poly(vinylpyrrolidone) (PVP, (C_6_H_9_NO)_n_, molecular weight, 4.4-5.4 kg/mol), copper(II) sulfate pentahydrate (CuSO_4_•5H_2_O, ≥99.0%), ascorbic acid (AA, ≥99%) and selenium dioxide (SeO_2_, ≥99%) were purchased from Sinopharm Chemical Reagent Co., Ltd. Chloroauric acid (HAuCl_4_•4H_2_O, 99% trace metals basis) was purchased from Shanghai Civi Chemical Technology Co., Ltd. Hexadecyltrimethylammonium bromide (CTAB, 99%), hexadecyltrimethylammonium chloride (CTAC, 97%) were purchased from Aladdin Reagent Company. Poly(sodium 4-styrenesulfonate) (PSS, molecular weight, 70 kg/mol) was purchased from Alfa Aesar. Poly(diallyldimethyl ammonium chloride) (PDDA, molecular weight, 100 kg/mol) was purchased from Shanghai Macklin Biochemical Technology Co., Ltd. Poly(ethylene glycol) monomethyl ether thiol (mPEG-SH, molecular weight, 5 kg/mol) was purchased from Hunan HuaTeng Pharmaceutical Co., Ltd. Calcein acetoxymethyl ester (calcein-AM) and propidium iodide (PI) were obtained from Shanghai Yeasen Biotech Co., Ltd. Cell Counting Kit-8 (CCK-8) assay kit was purchased from Dojingdo Laboratories (Kumamoto, Japan). Gold standard solution (1000 µg/mL) containing 1.5 M HCl, copper standard solution containing1.0 M HNO_3_ and selenium standard solution containing 2.0 M HNO_3_ were obtained from Beijing Guobiao Testing and Certification Co., Ltd. Fetal bovine serum (FBS), penicillin-streptomycin, Dulbecco modified Eagle's medium (DMEM), and trypsin-EDTA were acquired from Biological Industries Co. (Haemek, Israel). Ultrapure water with a resistivity of 18.2 MΩ•cm (at 25 °C) was produced with a Millipore Direct-Q3 UV system (Millipore Corporation, Molshein, France) and used throughout the experiments. All chemicals and solvents were of analytical grade and used as received. All glassware used in this work was cleaned with aqua regia, rinsed thoroughly with ultrapure water and then air-dried prior to use throughout all experiments.

### Instrumentation

UV-vis-NIR absorption spectra were collected using a Cary 5000 UV-vis-NIR spectrophotometer (Agilent Technology, USA). Powder X-ray diffraction (XRD) patterns were recorded with a SmartLab3kW X-ray diffractometer (Rigaku, Japan), operating at 40 kV and 30 mA using Cu Kα radiation (λ=1.5406 Å). Scanning electron microscopy (SEM) images were acquired on a FEI Helios Nanolab G3 UC dual-beam scanning electron microscope operating at 10 kV. Transmission electron microscopy (TEM) images and high-resolution TEM (HRTEM) images were taken using a FEI Tecnai G2 F20 transmission electron microscope operating at 200 kV. The samples were prepared by dropping the suspension on carbon-coated copper grids and being then drying under ambient conditions. The dimensional parameters of all samples were statistically measured from the TEM data using the ImageJ analysis software, and more than 150 nanoparticles were counted for each sample. The inductively coupled plasma-optical emission spectroscopy (ICP-OES) analysis was carried out on an Agilent 5100 inductively coupled plasma-optical emission spectrometer (Agilent Technologies, USA). The samples for ICP-OES measurements were digested in a concentrated nitric acid and then diluted with ultrapure water prior to analysis. The hydrodynamic size and zeta potential were measured using dynamic light scattering (DLS) and electrophoretic light scattering (ELS) on a Malvern Zetasizer Nano-ZS ZEN3600 analyzer (Malvern Instrument Ltd., UK), respectively. Fourier transform infrared (FTIR) spectroscopy was conducted on a Thermo Nicolet 6700 spectrometer.

### Synthesis of CTAB-modified gold nanorods (GNRs)

The CTAB-modified GNRs were synthesized using a seed-mediated growth method previously reported in the literature 29,40, with a slight modification for massively producing GNR stock solution to avoid the batch-to-batch variation. Briefly, to prepare the gold seed solution, 0.25 mL of 0.01 M HAuCl_4_ was added to 9.75 mL of 0.1 M CTAB solution under vigorous stirring, followed by the rapid injection of freshly prepared, ice-cold NaBH_4_ aqueous solution (0.6 mL, 0.01 M). A light yellow-to-brown color change was immediately seen, indicating the formation of gold seeds. After reacting for 10 sec, the gold seed solution was kept still at 30 ºC to age for 2 h prior to use. For preparation of the GNR suspension, the growth solution was prepared by the successive addition of 5 mL of 0.05 M HAuCl_4_ aqueous solution and 2.5 mL of 0.02 M AgNO_3_ aqueous solution to 500 mL of 0.1 M CTAB aqueous solution. To the growth solution was added 10 mL of 1 M HCl for the pH adjustment, and then 1.6 mL of 0.2 M AA was added. Once the reaction solution became colorless from the dark yellow, 1.5 mL of the gold seed solution obtained above was rapidly injected and the reaction continued for overnight at 30 ºC. The GNRs were purified by successive centrifugation and washing at least twice with ultrapure water. The resultant GNR pellets were re-dispersed into a 5 mM CTAB solution, producing the CTAB-modified GNR suspension with a concentration of 167 mg/L, estimated by ICP-OES.

### Surface modification of gold nanorods

CTAC-, PVP-, PSS- or PDDA-modified GNRs were prepared by a ligand exchange method following the well-established protocols in the literature 41,42. The CTAC-modified GNRs were prepared by centrifugation of as-prepared CTAB-modified GNRs at 8000 rpm for 30 min and then washing twice with ultrapure water, followed by redispersion of the pellets in a 5 mM CTAC aqueous solution. For preparation of PVP-, PSS- or PDDA-modified GNRs, the CTAB-modified GNRs were first centrifuged at 8000 rpm for 30 min and washed with ultrapure water. The pellets were re-dispersed in an aqueous solution containing 0.15 wt% PSS, and after incubation for 2 h the suspension was centrifuged at 8000 rpm for 30 min and re-dispersed in an aqueous solution containing 0.15 wt% PSS. The centrifugation-redispersion process was repeated three times to minimize the CTAB on the GNRs. The resultant pellets were PSS-modified GNRs, which were re-dispersed in an aqueous solution containing 0.05 wt% PSS for further use. To prepare PVP- or PDDA-modified GNRs, the PSS-modified GNRs were centrifuged and re-dispersed in a solution of 5 mM sodium citrate, and after incubation for 12 h the citrate-modified GNRs were obtained. Following this, the citrate-modified GNRs were centrifuged at 9000 rpm for 20 min and then re-dispersed in a solution of 20 mg/mL PVP or 10 mg/mL PDDA. As a result, PVP- and PDDA-modified GNRs were produced. Thus, we obtained CTAB-, CTAC-, PVP-, PSS- or PDDA-modified GNRs, whose concentrations all were adjusted to be 167 mg/L for the following use.

### Synthesis of Cu_2-x_Se nanocrystals (NCs)

Cu_2-x_Se NCs were synthesized following a slightly modified protocol from our previous work 5. Specifically, 200 μL of 0.1 M AA aqueous solution and 50 μL of 0.05 M SeO_2_ were added to 2 mL of the aqueous solution containing 10 mg/mL PVP. After being stirred for 10 min, 50 μL of 0.1 M CuSO_4_•5H_2_O was added simultaneously with 400 μL of 0.1 M AA solution under vigorous stirring. The reaction continued until a dark green color appeared, indicating the formation of Cu_2-x_Se NCs. The Cu_2-x_Se NCs were purified by centrifugation at 9000 rpm for 10 min and then washing twice with ultrapure water, followed by re-dispersion in 2.5 mL ultrapure water for further use.

### Overgrowth of Cu_2-x_Se on gold nanorods

The overgrowth of Cu_2-x_Se on GNRs was carried out by a Se template method. CTAB-, CTAC-, PVP-, PSS- or PDDA-modified GNRs obtained above were used for the synthesis of GNR@Cu_2-x_Se hybrids, respectively. First, 1.0 mL of 167 mg/L GNRs modified with various capping agents and 200 μL of 0.1 M AA aqueous solution were successively added to 1.0 mL of ultrapure water, followed by the addition of various SeO_2_ concentrations (0.28, 0.56 and 1.12 mM). After being stirred for 10 min, CuSO_4_•5H_2_O of various concentrations (0.56, 1.12 and 2.24 mM) were added together with 400 μL of 0.1 M AA under vigorous stirring. After the reaction continued for overnight, the products were purified by centrifugation at 7500 rpm for 15 min and washing at least twice with ultrapure water. The resultant pellets were the GNR@Cu_2-x_Se hybrids re-dispersed in 2.5 mL ultrapure water, whose concentrations were determined by ICP-OES.

### Evaluation of the photothermal performance

#### Experimental Measurements

To evaluate the photothermal conversion efficiency, the temperature of aqueous suspensions of GNRs or GNR@Cu_2-x_Se hybrids with the gold concentration of 100 μg/mL was recorded as a function of time with an FLIR A35 infrared thermal camera under the continuous irradiation of either 808 nm or 1064 nm laser. In the case of Cu_2-x_Se NCs, the concentration was set to be 100 μg/mL. The power density was adjusted to be 1.0 W/cm^2^ for both 808 nm and 1064 nm lasers. Following continuous irradiation for 10 min, the laser was switched off and then the suspensions naturally cooled down to room temperature. Five consecutive laser on/off cycles were repeated to investigate the stability of the photothermal performance. The photothermal conversion efficiency (*η*) was determined by the following equation 43-45:


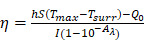
(1)

where *I* and *A*_λ_ are the incident laser power and optical density of the suspension at the wavelength *λ*, respectively; *h* and *S* are the heat transfer coefficient and surface area of the container covered by the sample, respectively; *T*_max_ and *T*_surr_ represent the steady-state temperatures under the laser irradiation of the sample suspensions and water solvent, respectively; *Q*_0_ represents the heat dissipated due to the absorption of the incident laser at the wavelength *λ* by the sample container only containing the solvent, which can be calculated by *Q*_0_=5.4×10^-4^×*I* (J/s). The *hS* can be derived from the following equation 43-45:


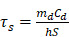
(2)

where *m*_d_ and *C*_d_ are the mass and heat capacity (4.2 J/g) of the water solvent, respectively. The time constant (*τ*_s_) of the suspension system can be determined by the linear fitting of the *t* vs -ln*θ* relationship during the cooling period after the laser is switched off.


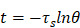
(3)


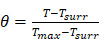
(4)

#### Numerical Simulations

The Finite-Difference Time-Domain (FDTD) simulations of optical absorption spectra were performed by using the commercial software Lumerical FDTD solutions. The GNR was modeled as a cylinder capped with two hemi-ellipsoidal ends, created on the basis of its dimensional parameters of 15.8 nm in width × 57.5 nm in length extracted from the experimental TEM images (Figure S1). The dimensional parameters of the Cu_2-x_Se domain were statistically measured from the TEM images of their respective GNR@Cu_2-x_Se structures and used for the modeling. The dielectric function of gold was taken from the experimental data measured by Johnson and Christy 46, while the dielectric function (*ε*(*ω*)=*ε*_1_(*ω*)+ i*ε*_2_(*ω*)) of Cu_2-x_Se was calculated from Cu_2-x_Se NCs according to the Drude model 5,47:


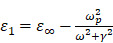
(5)


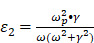
(6)

where *ε*_∞_ and *ω*_p_ represent the high frequency dielectric permittivity and the plasma frequency of the free carriers in the Cu_2-x_Se domain, respectively; *γ* is the free carrier damping numerically equal to the full-width-at-half-maxima (fwhm) of the plasmon band. *ε*_∞_ is equal to 10 for Cu_2-x_Se used in this work according to the literature 47, and *ω*_p_ can be derived from the plasmon resonance frequency (*ω*_sp_) by the following formula:


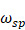
=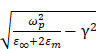
(7)

The dielectric constant (*ε*_m_) of the surrounding medium (water) is calculated from its background refractive index (*n*_m_=1.33) by *ε*_m_=* n*_m_^2^=1.33^2^=1.77. Thus, with the plasmon resonance frequency and the *γ* value (0.43 eV), we obtained the *ω*_p_ value of 4.62 eV for Cu_2-x_Se.

Utilizing the same geometric models above, we calculated the distributions of electric field intensity, steady-state temperature and power volume density of GNRs, Cu_2-x_Se NCs and GNR@Cu_2-x_Se heterostructures excited by a continuous-wave (CW) 1064 nm laser with COMSOL Multiphysics 5.4 (COMSOL Inc., USA), a commercial finite element analysis software. In general, the plasmonic structures first absorb the incident light to generate heat energy, followed by the heat transfer toward the base medium. With the excitation of a CW laser, the transient-state temperature (***T***) throughout the plasmonic structure and its surroundings can be described by the following formula 48,49:


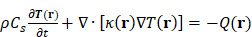
(8)

where ***T*** are both position (***r***)- and time (*t*)-dependent; *ρ*, *C*_s_ and* κ* are the position-dependent mass density, specific heat capacity and thermal conductivity of the materials, respectively; and *Q* is the power volume density of external heat source. In this work, the heat source is the optical absorption of the incident laser, which is locally proportional to the intensity of the incident electric field (**E**(**r**, *t*)) and the imaginary part of the dielectric function (ε(**r**, *ω*)) within the materials, written as follows 48,49:


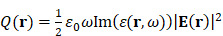
(9)

where *ε*_0_ and *ω* are the vacuum permittivity and frequency of the incident light, respectively. Since we are focusing on the steady-state temperature distribution in this work, the time derivative of the temperature (∂*T*(**r**)/∂*t*) in the first term at the left of Eq 8 is zero. We can obtain both optical properties and electric field distribution, and then generate the total absorption power (*P*(**r**, *ω*)) spectrum by 50:


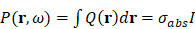
(10)

where *σ*_abs_ and *I* represent the absorption cross-section calculated from the FDTD simulations and the light intensity of the incident laser, respectively. In this calculation, the thermal conductivity, *κ*, of the Cu_2-x_Se was adopted from the literature 51, the incident power density and ambient temperature were set to 1 mW/μm^2^ and 298 K, respectively. The simulation volume is 300 nm × 300 nm × 300 nm with the perfectly matched layer boundary used.

### Cell culture and *in vitro* cytotoxicity

Breast cancer MDA-MB-231 cells and human normal liver cell line L-02 cells were originally obtained from American Type Culture Collection (ATCC). Cells were incubated at 37 °C in a 5% CO_2_ incubator in DMEM supplemented with 1% penicillin/streptomycin and 10% fetal bovine serum (FBS).

The cytotoxicity of samples was assessed against human normal liver cell line L-02 cells and breast cancer MDA-MB-231 cells by a standard CCK-8 viability assay. Cells were first seeded into 96-well plates at a density of 1×10^4^ cells/well (n = 5). After incubation for 24 h, the culture medium was removed and the cells were washed twice with PBS. For evaluation of *in vitro* cytotoxicity against L-02 cells, the fresh culture media containing GNR@Cu_2-x_Se hybrids made with PVP (0 (control), 25, 50, 75, 100, 150, and 200 μg/mL for gold) were added and incubated for 24, 48 and 72 h, respectively. For evaluation of *in vitro* cytotoxicity against MDA-MB-231 cells, the fresh culture media containing various samples including GNRs (100 μg/mL), Cu_2-x_Se NCs (200 μg/mL) or GNR@Cu_2-x_Se hybrids (100 μg/mL for gold) were added and incubated for 24 h. Following this, 100 μL of the DMEM medium containing 10% CCK-8 solution was added to each well and incubated at 37 °C for another 4 h. Afterwards, the absorbance (*A*_s_) of each well was measured at 450 nm on a Tecan Spark microplate reader (Männedorf, Switzerland). The absorbance (*A*_c_) of the wells without samples was measured as control for cell viability calculation: cell viability (%) =*A*_s_/*A*_c_×100.

### *In vitro* NIR-II photothermal cell ablation

To evaluate the *in vitro* NIR-II photothermal cell ablation, MDA-MB-231 cells were seeded in 96-well plates at a density of 1×10^4^ cells/well and incubated at 37 °C in a 5% CO_2_ incubator for 12 h. Then, the culture media were replaced with 100 μL of the DMEM medium containing GNRs (100 μg/mL), Cu_2-x_Se NCs (200 μg/mL) or GNR@Cu_2-x_Se hybrids (100 μg/mL for gold), followed by incubation for another 12 h. Afterwards, the cells were rinsed with PBS and then exposed to irradiation of a 1064 nm laser for 5 min at a power density of 1.0 W/cm^2^ with the 5 cm-distance between the laser and the cells. After that, the cells were rinsed with PBS and the cell viability was evaluated by the CCK-8 assay. The cells without the laser irradiation but with all other conditions identical were used as a control.

We further performed the live/dead assay using the Calcein AM and PI co-staining approach for the GNR@Cu_2-x_Se structures made with PVP. MDA-MB-231 cells were seeded in a 12-well plate at a density of 1×10^5^ cells/well and incubated in DMEM for 12 h, followed by the replacement of the culture medium with the fresh DMEM medium containing the GNR@Cu_2-x_Se structures with the same gold concentration aforementioned. After incubation for 12 h, each well was subjected to the 1064 nm laser irradiation at a power density of 1.0 W/cm^2^ for 5 min, the cells were rinsed three times with PBS and then co-stained with Calcein AM and PI for 15 min. After that, the cells were imaged on an inverted fluorescence microscope (Axio Vert. A1, Carl Zeiss). Control experiments were conducted using either 1064 nm laser only or GNR@Cu_2-x_Se hybrids only with all other conditions identical.

### *In vivo* NIR-II photothermal antitumor performance and *in vivo* biodistribution

The female nude mice (4 weeks, 13-16 g) were purchased from Chang Zhou Cavens Laboratory Animal Ltd. (Changzhou, China). All animal experiments were performed in accordance with the standards established by the Guidelines for the Care and Use of Laboratory Animals by Central South University and approved by the Management Committee of the Central South University. For *in vivo* studies, the breast tumor model was established by subcutaneous injection of 100 μL MDA-MB-231 cells in PBS (5×10^6^ cells) into the right frank region using 50% (v/v) Matrigel. When the tumors grew to ~150 mm^3^, the mice were employed for *in vivo* experiments. All MDA-MB-231 tumor-bearing mice were intratumorally injected with 50 μL of GNR@Cu_2-x_Se hybrids containing the gold concentration of 200 μg/mL. After injection for 2 h, the mice were irradiated for 5 min with a 1064 nm laser at a power density 1.0 W/cm^2^, which can be achieved at a 5 cm distance between the laser and the tumor. The real-time temperatures and photothermal images of mice were recorded by an FLIR A35 infrared thermal camera over the irradiation time. The laser was adjusted to center the laser spot on the tumor and completely cover the entire tumor. The tumor volume and body weights were measured every other day for individual animals in all experiments. The tumor volume was calculated by the following formula: tumor volume = ((tumor length) × (tumor width)^2^)/2. After 15 days of the photothermal treatment, the mice were sacrificed, and the tumors were collected, weighted and then sectioned for *ex vivo* analysis.

### Statistical analysis

At least three independent experiments were carried out throughout the study, and all data were presented as mean ± standard deviation (SD). Statistical analysis was performed by student's *t*-test using the SPSS-18 software. The statistical significance was indicated as *P < 0.05, **P < 0.01, and ***P < 0.001.

## Results and Discussion

### Regioselective overgrowth for synthesis of GNR@Cu_2-x_Se heterostructures

The initial GNRs were produced by a conventional seed-mediated synthetic route using CTAB as the surfactant 29,40, leading to CTAB-modified GNRs with the average length × width of 57.5±6.1 nm × 15.8±2.6 nm (Figures S2 and S3). CTAB-modified GNRs were further converted to CTAC-, PVP-, PSS- and PDDA-modified GNRs according to the well-established protocols in the literature 41,42. The direct ligand exchange between CTAB and CTAC produced the CTAC-modified GNRs. Anionic PSS could be used as a detergent for removing CTAB adsorbed on GNRs, followed by the ligand exchange with nonionic PVP and cationic PDDA, respectively. This leads to PVP- and PDDA-modified GNRs, respectively. Surface modification with these capping agents did not result in observable change of plasmon bands with respect to that of CTAB-modified GNRs, indicating no significant aggregation (Figure S4). The CTAC-modified GNRs display a zeta potential of +40.2 mV, slightly more positive with respect to +36.5 mM of CTAB-modified GNRs, and PDDA-modified GNRs have a zeta potential of +32.9 mV, while PVP- and PSS-modified GNRs are negatively charged with zeta potential values of -19.2 and -41.6 mV, respectively. The successful surface modifications of GNRs with different capping agents are further confirmed by FTIR measurements (Figure S5). All these results corroborate that CTAB-, CTAC-, PVP-, PSS- and PDDA-modified GNRs are successfully prepared by the ligand exchange method without significant aggregation.

**Figure 1:**
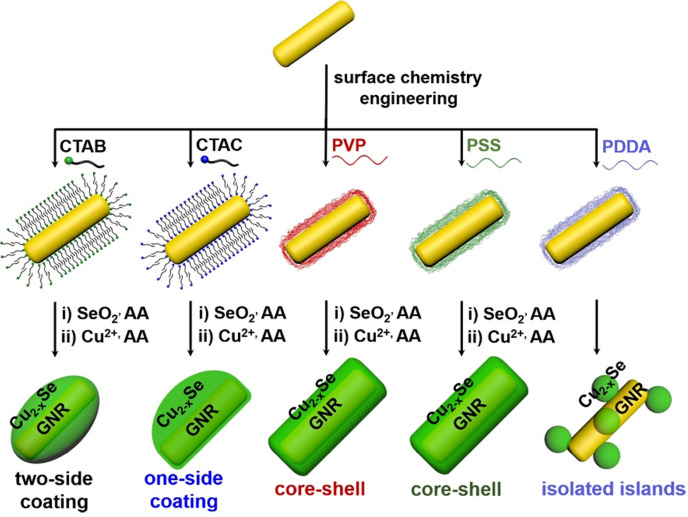
** Schematic illustrating the regioselective overgrowth of Cu_2-x_Se on GNRs and their characterizations**. (A) Schematic illustration of surface chemistry-mediated regioselective growth of Cu_2-x_Se on GNRs with various capping agents in a Se template method, including the controllable deposition of Se layer by the reduction of SeO_2_ with the AA reducing agent and its subsequent conversion into Cu_2-x_Se in the presence of Cu^2+^ and AA. (B) Zeta potentials of CTAB-, CTAC-, PVP-, PSS- and PDDA-modified GNRs, their derived GNR@Se hybrids, GNR@Cu_2-x_Se heterostructures, and PEGylated GNR@Cu_2-x_Se heterostructures in PBS, respectively. (C) Powder XRD patterns of GNRs, GNR@Se core-shell hybrids and GNR@Cu_2-x_Se core-shell heterostructures prepared with 1.12 mM SeO_2_ in the presence of PVP. Powder XRD patterns of all other types of GNR@Cu_2-x_Se heterostructures are shown in Figure S6 in the Supplementary Material. The standard patterns of cubic gold (JCPDS # 01-1172) and cubic berzelianite Cu_2-x_Se (JCPDS # 06-0680) are shown as well.

The preparation of GNR@Cu_2-x_Se heterostructures involves a two-step process - (i) the initial deposition of elemental Se on the GNR surface by the reduction of SeO_2_ with AA as the reducing agent to form the GNR@Se hybrids, and (ii) the subsequent conversion of GNR@Se hybrids into GNR@Cu_2-x_Se heterostructures with simultaneous addition of Cu^2+^ and AA (Figure 1A) 5,25. The strategy is inspired by the high affinity of elemental chalcogens and chalcogenides toward noble metals (*i.e.*, gold, silver), widely adopted in the literature for synthesis of noble metal-semiconductor heterostructures with large lattice-mismatch 43,44. The large lattice-mismatch between gold and Cu_2-x_Se does not allow the direct heteroepitaxial growth of Cu_2-x_Se on the GNR surface (Table S1). Nevertheless, this study exploits the amorphous Se template for synthesis of GNR@Cu_2-x_Se heterostructures, which could overcome the issue of the large lattice-mismatch. The deposition of amorphous elemental Se and the subsequent conversion into Cu_2-x_Se cause insignificant change of their zeta potentials with respect to their corresponding capping agent-modified GNRs (Figure 1B). The absence of characteristic peaks of elemental Se in the XRD pattern of GNR@Se hybrids made with PVP and 1.12 mM SeO_2_ confirms the amorphous form of the deposited Se (Figure 1C). In addition to the intense XRD peaks at 2θ=38.1° and 77.5° both assigned to the (111) and (311) planes of the face-centered cubic gold phase from the GNRs, these GNR@Cu_2-x_Se hybrids exhibit consistent XRD peaks at 2θ=26.7°, 44.6°, 52.9° and 65.0° all indexed to the (111), (220), (311) and (400) planes of the cubic berzelianite Cu_2-x_Se phase, respectively (Figures 1C and S6).

**Figure 2:**
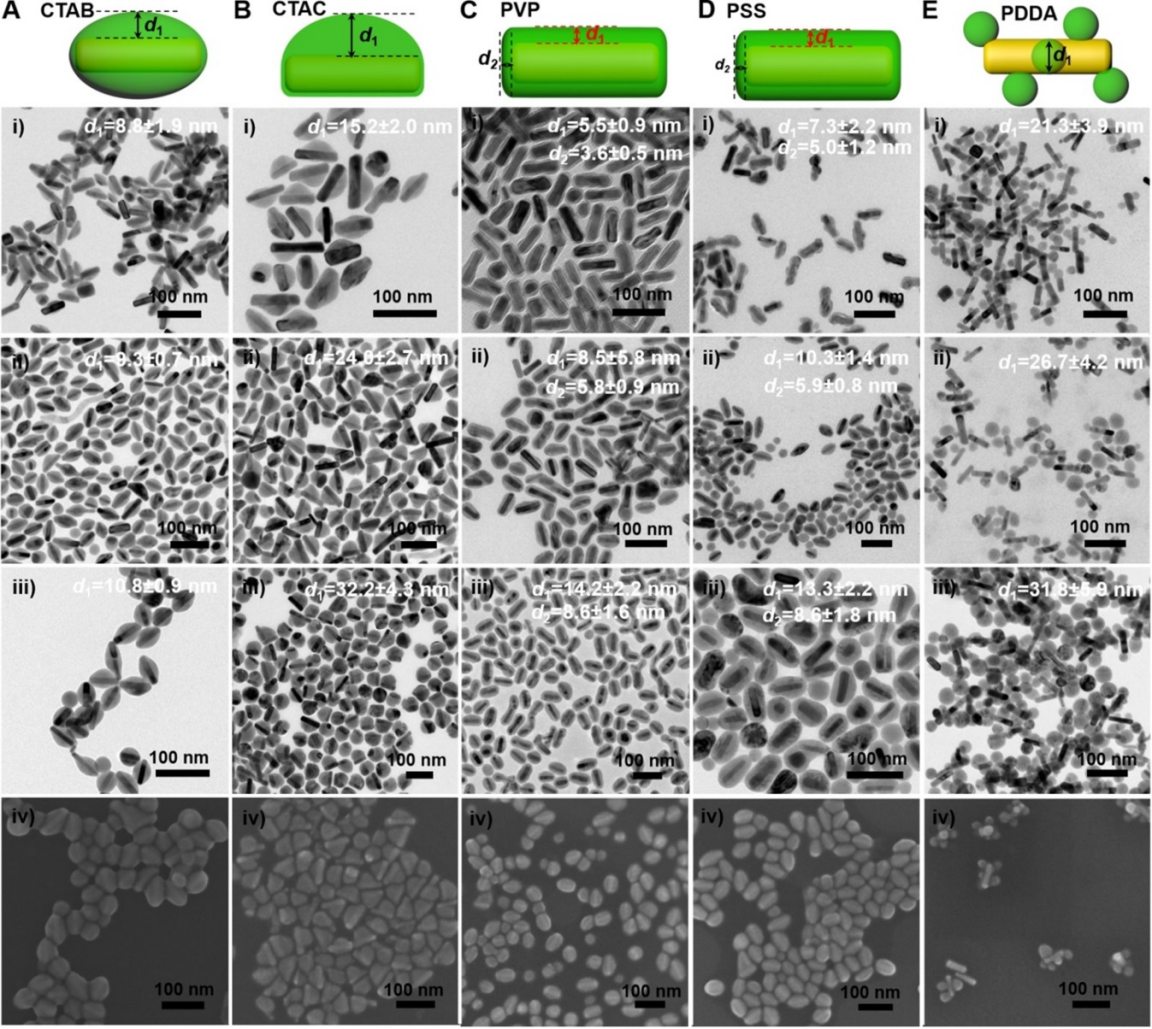
** Morphology of GNR@Cu_2-x_Se heterostructures**. (A-E) (i-iii) TEM images of GNR@Cu_2-x_Se heterostructures prepared with (A) CTAB, (B) CTAC, (C) PVP, (D) PSS, and (E) PDDA in the presence of (i) 0.28 mM, (ii) 0.56 mM and (iii) 1.12 mM SeO_2_, and (iv) their corresponding SEM images prepared with 0.56 mM SeO_2_. Schematic of each type of GNR@Cu_2-x_Se structures is shown as well in the top panel. The dimensional parameters (*d*_1_ and *d*_2_) of each type of GNR@Cu_2-x_Se structures were statistically measured from their TEM images for each SeO_2_ concentration, which are shown along with the corresponding TEM images as well as in Figure S22 of the Supplementary Material.

**Figure 3:**
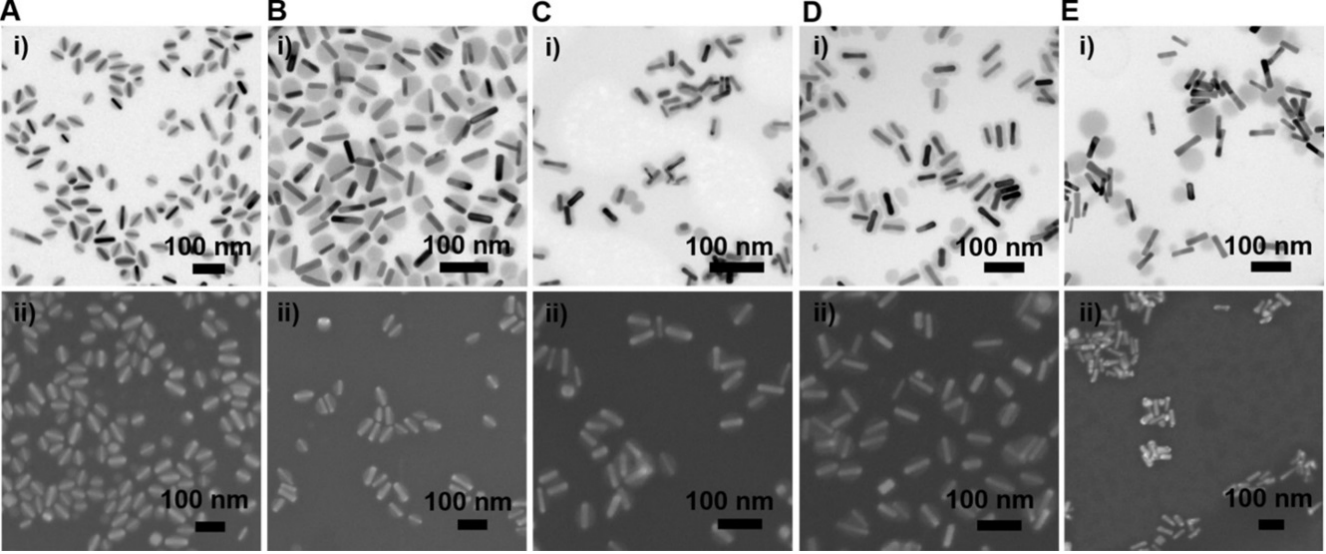
** Morphology of GNR@Se hybrids prepared with various capping agents**. (A-E) (i) Representative TEM and (ii) SEM images of GNR@Se structures in the presence of 0.56 mM SeO_2_ prepared with (A) CTAB, (B) CTAC, (C) PVP, (D) PSS and (E) PDDA.

We further examined the morphology of the GNR@Cu_2-x_Se heterostructures derived from GNRs modified with various capping agents using TEM and SEM (Figure 2A-E and Figures S7-S16). TEM images show the exclusive overgrowth of Cu_2-x_Se on the lateral two-sides of CTAB-modified GNRs with the thickness highest at the center and decreasing from the center to the end, forming a rice-like GNR@Cu_2-x_Se heterostructures. Surprisingly, the Cu_2-x_Se overgrowth on only one side of the CTAC-modified GNRs proceeds. Conformal coating of Cu_2-x_Se on the GNRs takes place to form the core-shell GNR@Cu_2-x_Se heterostructures for both PVP- and PSS-modified GNRs, with the Cu_2-x_Se layer a little thicker on the lateral two-sides than at the two-ends. However, the island growth mode of Cu_2-x_Se on the GNRs is preferred with PDDA. Thus, we can clearly see the capping agent-mediated regioselective overgrowth of Cu_2-x_Se in a specific region of the anisotropic GNRs using the present Se template approach. To account for the surface chemistry dependent difference of the Cu_2-x_Se overgrowth, we carefully checked the morphology of these GNR@Se hybrids by both SEM and TEM, as shown in Figure 3 and Figures S17-S21. We found that both GNR@Se and GNR@Cu_2-x_Se structures had the similar morphology for each type of capping agent. Conversion of GNR@Se hybrids into their GNR@Cu_2-x_Se counterparts inherited their initial morphology without observable changes in all cases of CTAB, CTAC and PDDA; As we can see from Figure 3C,D and Figures S19, S20, heavy aggregation and relatively rough Se shells of the GNR@Se core-shell hybrids were observed for both PVP and PSS probably resulting from the coalescence of the Se shells due to the weak interaction between the elemental Se and capping agents, compromising the surface protection. However, the high monodispersity of GNR@Cu_2-x_Se heterostructures and uniformity of Cu_2-x_Se shells suggest much stronger capping agent-Cu_2-x_Se interaction for both PVP and PSS, significantly improving the colloidal stability of GNR@Cu_2-x_Se heterostructures.

**Scheme 1:**
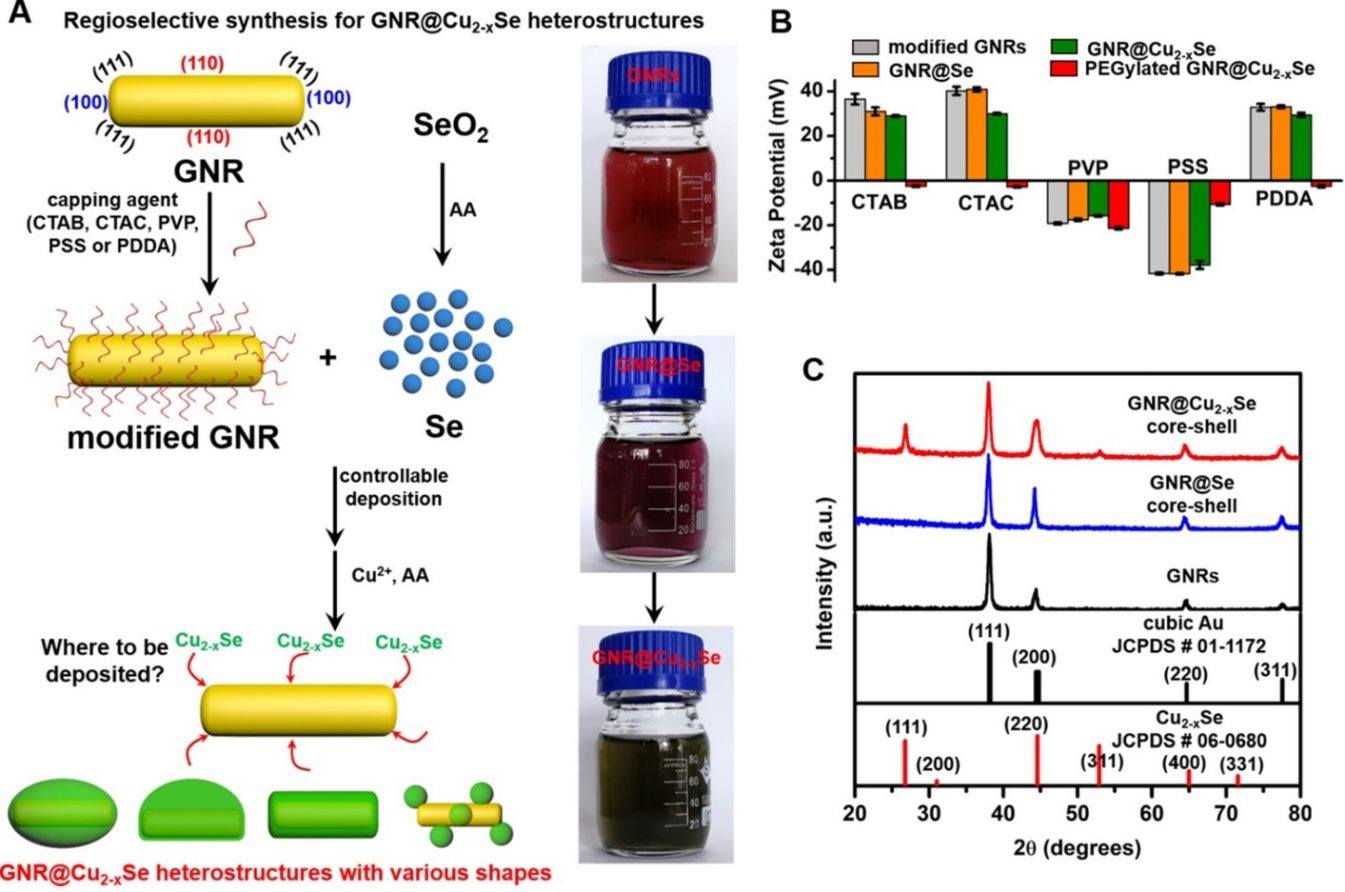
Capping agent-mediated overgrowth of Cu_2-x_Se on the GNRs for synthesis of GNR@Cu_2-x_Se heterostructures via a Se template method.

Thus, the morphology of GNR@Cu_2-x_Se hybrids is principally determined by the initial GNR@Se structures, essentially preferential deposition of elemental Se dictated by capping agents. As is well-known, the overgrowth of a second material is governed by several comprehensive factors such as lattice mismatch, interfacial energy, binding affinity and surface blocking effect 32,35,45. The lattice mismatch is not the influencing factor in this study since the Se layer deposited on the GNRs is in an amorphous form. Electron microscopic studies showed that CTAB-modified GNRs made by the seed-mediated growth route constituted distinctive crystal facets with (110) facets on the lateral sides, (100) at the tips and (111) at the shoulders, schematically illustrated in Figure 1A 46-48. These crystal facets have distinct specific surface energy (*γ*) in the order of *γ*(111) < *γ*(100) < *γ*(110) 49. Thus, CTAB molecules are preferentially adsorbed on the (110) side facets with a higher ligand packing density than those at the tips and shoulders 36,50. The blocking effect due to the strong adsorption of Br^-^ of CTAB on the (100) facets results in the selective deposition of elemental Se on the lateral sides with high surface energy of CTAB-modified GNRs 35,51, forming a rice-like GNR@Se structure. Both CTAB and CTAC have similar quaternary ammonium cations but different halide anions (Br^-^ and Cl^-^), the site-blocking effect cannot explain unidirectional overgrowth of the Se layer on one side of the CTAC-modified GNRs since the crystal facets along all parallel lateral sides are expected to be the same. We infer that the local surface strains initialized by the deposition of Se atoms are responsible for the growth of Se on one side of GNRs, which could break the growth symmetry of the Se layer previously observed in the overgrowth of gold on the GNR surface 52. The negative charge of PVP- or PSS-modified GNRs induces the conformal symmetric growth of the Se layer due to the electrostatic interaction; however, the island growth mode is dominant when PDDA-modified GNRs are used. PDDA is a positively charged polymer so that PDDA forms a stable and dense PDDA layer around the GNR surface, preventing the facet selective growth of Se, which is observed in cases of both CTAB and CTAC 25. Thus, we substantiate that changing the capping agents of the GNRs could control the growth of Cu_2-x_Se at a specific region of the GNR surface, producing GNR@Cu_2-x_Se heterostructures with distinct morphology (Scheme 1). We further adjusted the Cu_2-x_Se domain size through changing the concentration (0.28, 056 and 1.12 mM) of the SeO_2_ precursor (Figure 2A-E and Figures S7-S16). The Cu_2-x_Se domain size increases with more SeO_2_ addition for all types of GNR@Cu_2-x_Se structures (Figure S22). Even with high SeO_2_ concentration, growth of the Cu_2-x_Se layer proceeds only on two sides and one side of the GNRs with CTAB and CTAC capping agents, respectively. The HRTEM image also verifies the cubic berzelianite crystal structure of Cu_2-x_Se consistent with the results from XRD measurements (Figure S23 and Figure 1C). GNR@Cu_2-x_Se structures with a high yield can be achieved with only few isolated Cu_2-x_Se nanoparticles observed in all these structures. The quantitative measurement by ICP-OES reveals an increase in weight percentage of Cu_2-x_Se ranging from 35-70 wt% in all heterostructures with the increasing SeO_2_ addition (Table 1). All results above confirm the regioselective overgrowth of Cu_2-x_Se on the GNRs mediated by the capping agents in the present Se template method, and the Cu_2-x_Se domain size can be finely adjusted by changing the SeO_2_ concentration.

### Optical properties and photothermal performance

**Figure 4:**
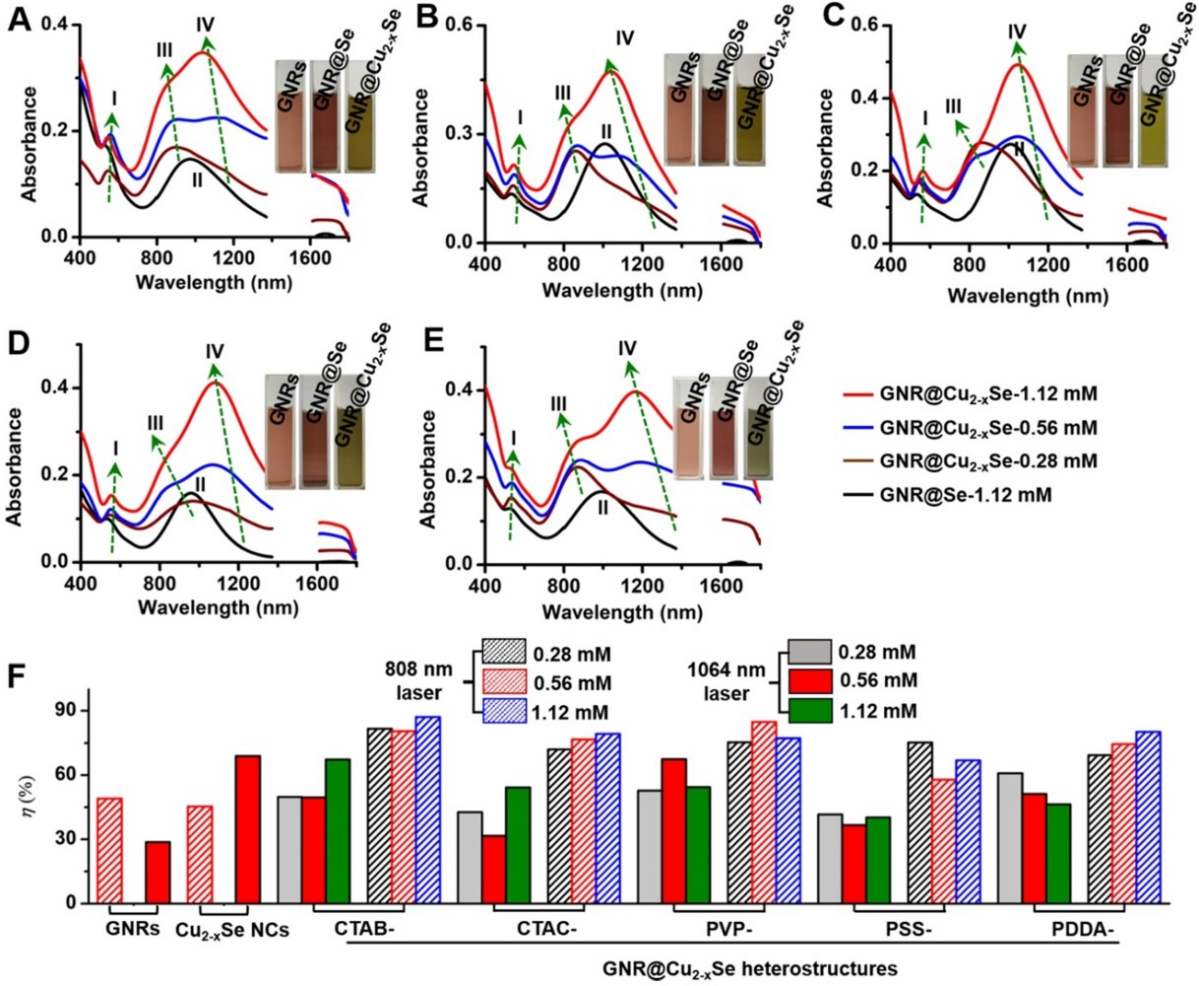
** Optical properties and photothermal performance of GNR@Cu_2-x_Se heterostructures**. (A-E) Optical absorption spectra of GNR@Cu_2-x_Se heterostructures prepared with (A) CTAB, (B) CTAC, (C) PVP, (D) PSS and (E) PDDA capping agents in the presence of 0.28 mM (wine curve), 0.56 mM (blue curve) and 1.12 mM (red curve) SeO_2_. Optical absorption spectra of the corresponding GNR@Se hybrids prepared with 1.12 mM SeO_2_ (black curve) are shown as well for each type of capping agent. Insets in each panel are optical photographs of aqueous suspensions of capping agent-modified GNRs, GNR@Se hybrids and GNR@Cu_2-x_Se hybrids prepared with 1.12 mM SeO_2_. Four plasmon bands are labeled as plasmon bands I, II, III and IV. (F) Photothermal conversion efficiency under either 808 nm (NIR-I)- or 1064 nm (NIR-II)-laser of aqueous suspensions of GNRs, Cu_2-x_Se NCs and GNR@Cu_2-x_Se structures prepared with various capping agents in the presence of 0.28, 0.56 and 1.12 mM SeO_2_, respectively.

Optical absorption spectra of GNR@Se hybrids show two characteristic bands at ~540 and ~1005 nm, which correspond to the transverse and longitudinal plasmon resonance of GNRs labeled as plasmon bands I and II, respectively (Figure 4A-E). The large redshifts (> 25 nm for plasmon band I, > 200 nm for plasmon II) with respect to those of the starting GNRs are chiefly due to the higher refractive index (n> 2.7 at 500-2000 nm) of amorphous Se in comparison with that (n = 1.33) of the water medium (Figure S1) 5,53. The conversion of the GNR@Se hybrids into their GNR@Cu_2-x_Se counterparts was accompanied by the blueshift of the plasmon band II to plasmon band III, the blueshift of which becomes more obvious with the increasing Cu_2-x_Se percentage. In addition, we observed an intense plasmon band IV in the NIR-II region originated from the Cu_2-x_Se domain, which is tunable in both wavelength and intensity in the wavelength range of 1050-1250 nm. The plasmon band IV blueshifts and becomes more intense with the increasing Cu_2-x_Se on the GNRs. Along with the conversion into the GNR@Cu_2-x_Se structures, the plasmon band I slightly redshifts with the increasing Cu_2-x_Se, contrary to the plasmon band III. These spectral changes are also reflected by the color change of these colloidal solutions, further justified by the theoretical calculations (insets in Figure 4A-E and Figure S1). We attribute these changes to the combined electronic interaction and plasmonic coupling interaction between the GNR and Cu_2-x_Se domains 5. Thus, our results corroborate the tunable NIR-II plasmon absorption in both wavelength and intensity for all GNR@Cu_2-x_Se structures investigated in this work through changing the SeO_2_ concentration. We also confirm the negligible change of optical absorption spectra of GNR@Cu_2-x_Se hybrids in suspensions of distinct pH values ranging from the tumor microenvironment to heathy physiological conditions, indicating excellent stability (Figure S24).

**Figure 5:**
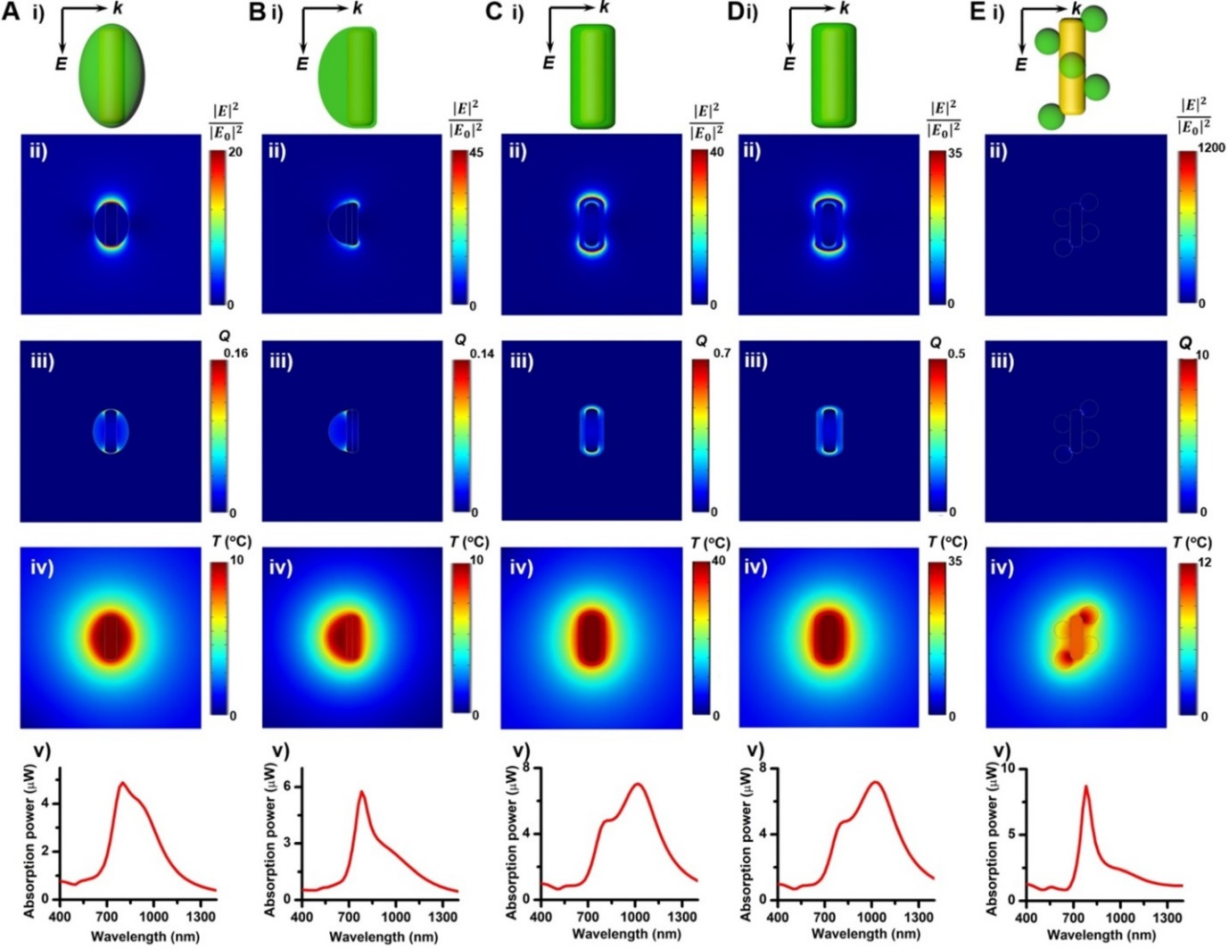
** Theoretical analysis of photothermal effects of a GNR@Cu_2-x_Se heterostructure in water prepared using various capping agents**. (A-E) (i) Schematic describing the model for the theoretical calculations of the optical properties and photothermal properties, distributions of (ii) calculated electric field intensity (|**E**|^2^/|**E**_0_|^2^) at 1064 nm, (iii) calculated heat power volume density (*Q*, nW/nm^3^) and (iv) calculated steady-state temperature (*T*, °C) under the NIR-II laser, and (v) absorption power spectra of a GNR@Cu_2-x_Se nanoparticle. The dimensional parameters were statistically measured from the TEM images of GNR@Cu_2-x_Se heterostructures made with 0.56 mM SeO_2_ using various capping agents. The electric field (**E**) and wavevector (***k***) orientation of the incident field is parallel and normal to the longitudinal axis of the GNR, respectively, as also shown in panels (i). The incident field intensity is 1 mW/µm^2^. The calculated results with the 808 nm excitation of a GNR, Cu_2-x_Se NC and GNR@Cu_2-x_Se structure are shown in Figure S38 in the Supplementary Material.

To evaluate the photothermal performance of these GNR@Cu_2-x_Se structures, aqueous suspensions of as-prepared GNR@Cu_2-x_Se structures with the same gold concentration were irradiated at a power density of 1.0 W/cm^2^ under either 808 nm- or 1064 nm-laser (Figure 4F). The photothermal effects of aqueous suspensions of GNRs and Cu_2-x_Se NCs were also measured as the control (Figures S2 and S25). The temperature profiles and photothermal images were recorded using an infrared thermal camera (Figures S26-S37). The photothermal conversion efficiency (*η*) was calculated on the basis of the time-constant for heat transfer and the maximum steady-state temperature, which are described in detail in the Methods section 54-56. We can get a* η* value as high as 49.1% under the 808 nm-laser and 28.7% under the 1064 nm-laser for GNRs. The *η* value of Cu_2-x_Se NCs is significantly higher with the 1064 nm-laser (70.0%) than that with the 808 nm-laser (45%). All these GNR@Cu_2-x_Se structures exhibit much higher *η* values under the 1064 nm-laser than under the 808 nm-laser, and we found that there existed slight differences among these *η* values under either laser irradiation for all these dual-plasmonic heterostructures, which may arise from the distinct Cu_2-x_Se percentage in these hybrids. We also find that these *η* values of these hybrids are much higher than that of Cu_2-x_Se NCs alone under the 808 nm-laser but much lower than that of Cu_2-x_Se NCs alone under the 1064 nm-laser. Our rationale is that the *η* value is closely dependent on the proportion of optical absorption converted into heat of the incident light power but not linearly related to the fraction of GNRs and Cu_2-x_Se in the heterostructures; however, the temperature increase is tightly related to the concentration of GNRs and Cu_2-x_Se because they significantly affect the total optical absorption. We attribute the high *η* value in GNR@Cu_2-x_Se hybrids developed in this work to the strong plasmonic coupling due to the large spectral overlap between the GNR and Cu_2-x_Se components. To further understand the photothermal performance of these heterostructures, we performed the theoretical analysis of the optical properties and photothermal properties of GNRs, Cu_2-x_Se NCs and GNR@Cu_2-x_Se heterostructures at the wavelength of 808 nm or 1064 nm (Figure 5 and Figure S38). The heterostructure is excited by a CW 1064 nm laser with the polarized electric field **E** along the longitudinal axis of the GNR and the light wavevector **k** along the transversal axis, as shown in Figure 5. Our results show the localized, non-uniform electric field distribution around the structures and the GNR-Cu_2-x_Se interface but the electric field is uniform within these structures. In contrast, the absorption power volume density is non-uniform inside the hybrids. The temperature distribution is nearly uniform inside the GNR domain different from the Cu_2-x_Se domain and surrounding medium, which is due to the different thermal conductivities between the substituents of heterostructures (*κ*(nanoscale gold) = 126 W/(m•K), *κ*(Cu_2-x_Se)=1.2 W/(m•K)) and the surrounding medium (*κ*(water)= 0.6 W/(m•K)) 57. The significantly larger thermal conductivity of gold with respect to the Cu_2-x_Se and water medium makes the heat diffusion fast inside the GNR domain so that thermal energy accumulates at the gold-Cu_2-x_Se interface. The thermal gradient in the Cu_2-x_Se domain is observed due to the small difference of thermal conductivities between Cu_2-x_Se and the water medium. There is no significant difference in the peak absorption power among these GNR@Cu_2-x_Se heterostructures.

In addition, we further examined the photothermal stability of these NIR-II plasmonic materials by repeating the photothermal experiment for five laser on/off cycles (Figures S26-S37). The temperature profiles of all these GNR@Cu_2-x_Se heterostructures almost remain unchanged after five cycles of consecutive laser on/off irradiation, suggesting the high photothermal stability of these materials. Thus, we conclude that all these GNR@Cu_2-x_Se heterostructures have excellent NIR-II photothermal performance with the *η* value ranging from 58-85% consistent with previously reported *η* values in the literature 26,58-60, highlighting their potential as durable photothermal agents for NIR-II photothermal tumor ablation.

### *In vitro* NIR-II photothermal studies

**Figure 6:**
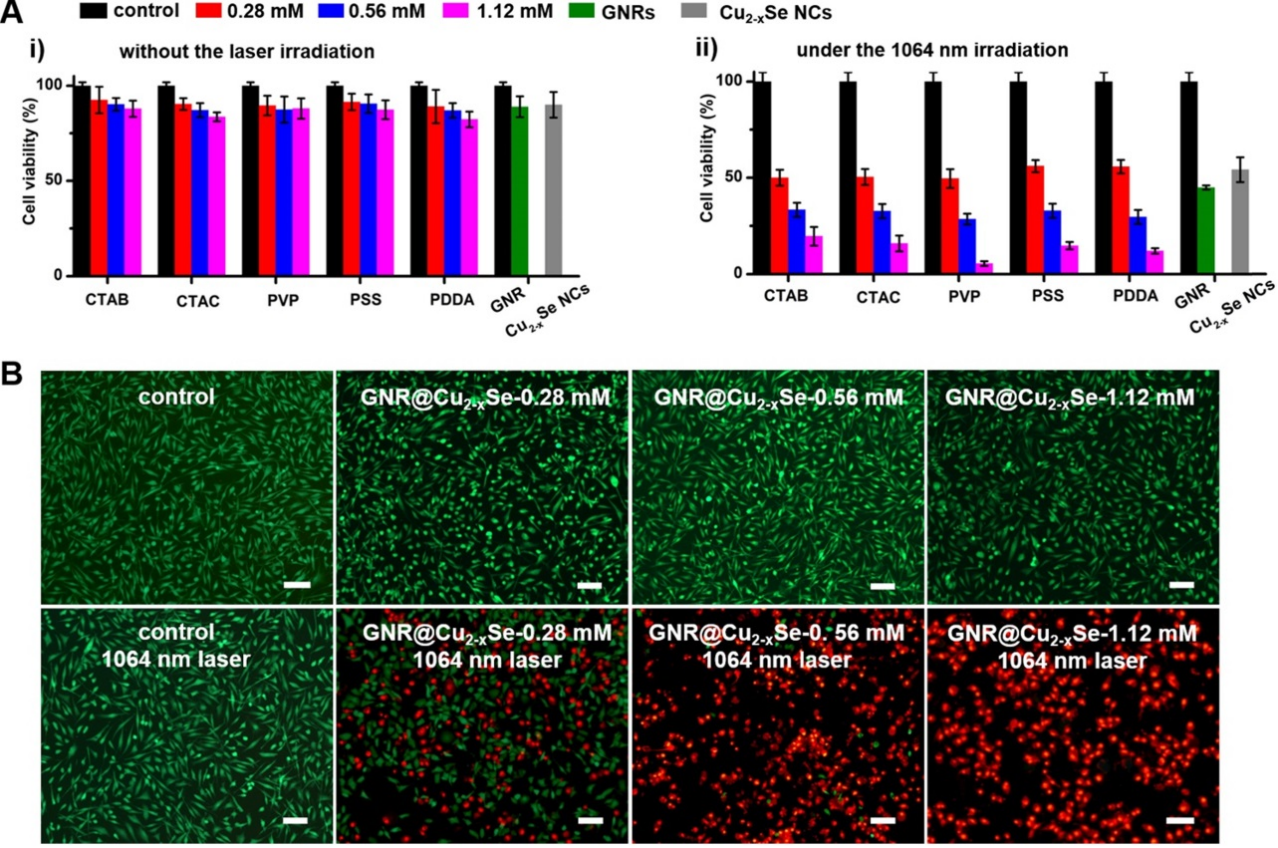
***In vitro* cytotoxicity and NIR-II photothermal ablation of cancer cells**. (A) Viability of breast cancer MDA-MB-231 cells after being incubated in culture medium containing GNRs (100 µg/mL), Cu_2-x_Se NCs (200 µg/mL) and GNR@Cu_2-x_Se hybrids (100 µg/mL for gold) prepared using various capping agents with 0.28, 0.56 and 1.12 mM SeO_2_ (i) for 24 h under no laser irradiation and (ii) under the NIR-II laser irradiation at a power density of 1 W/cm^2^ for 5 min. (B) Fluorescence images of live (green) and dead (red) cells stained by Calcein-AM and PI after being treated without (control) or with GNR@Cu_2-x_Se structures prepared using PVP-modified GNRs in the presence of 0.28, 0.56 or 1.12 mM SeO_2_ with or without the 1064 nm-laser irradiation. Scale bar: 100 µm.

The relatively high *η* value of these GNR@Cu_2-x_Se heterostructures permits their enormous potential as photothermal agents for NIR-II photothermal anticancer. We first modified the GNR@Cu_2-x_Se heterostructures with mPEG-SH to improve the biocompatibility and colloidal stability. The successful PEGylation of these GNR@Cu_2-x_Se structures was proved by the significant decrease of the zeta potential value (Figure 1B). The neutral or negative zeta potential after PEG modification could prevent these hybrids from aggregation and mitigate the detrimental electrostatic adsorption of various circulating biomolecules, thereby significantly improving their biocompatibility. The cytotoxicity of these GNR@Cu_2-x_Se structures was evaluated against human normal liver cell line L-02 cells and breast cancer MDA-MB-231 cells using a traditional CCK-8 assay. We did not observe the decrease of cell viability of L-02 cells even after incubation for 24, 48 and 72 h, respectively (Figure S39). A reduction of < 20% of the cell viability for MDA-MB-231 cells was observed even after incubation for 24 h in GNR@Cu_2-x_Se suspensions at a gold concentration of 100 μg/mL, suggesting good biocompatibility for all these plasmonic materials investigated in this work (Figure 6A). However, the cell viability significantly decreased after being irradiated with a NIR-II laser for 10 min at a power density of 1 W/cm^2^, and more cells were killed with the increasing Cu_2-x_Se percentage in the hybrids. Specifically, more than 80% of breast cancer MDA-MB-231 cells were killed, which was induced by the hyperthermia of GNR@Cu_2-x_Se heterostructures under the NIR-II laser. These results suggest that the NIR-II responsive Cu_2-x_Se component is responsible for the excellent photothermal ablation of cancer cells. It is worth noting that these dual-plasmonic heterostructures exhibit far superior NIR-II photothermal ablation performance than both individual GNRs and Cu_2-x_Se NCs, respectively, which is attributed to their strong coupling effects. Both excellent biocompatibility and *in vitro* NIR-II photothermal performance were further supported by the Calcein AM/PI live/dead cell staining assay, as shown in Figure 6B. No obvious dead cells were observed without the NIR-II laser and with either NIR-II laser or GNR@Cu_2-x_Se structures only, indicating the excellent biocompatibility and negligible laser damage to cells. By contrast, with both the addition GNR@Cu_2-x_Se heterostructures and the irradiation of the NIR-II laser, a fraction of dead cells (red fluorescence) were observed with more cells killed by the increased Cu_2-x_Se weight percentage, which is attributed to the hyperthermia effect induced by strong NIR-II absorption of the present dual-plasmonic heterostructures. These findings are in good agreement with the phototoxicity results from the CCK-8 assay, indicating that the present dual-plasmonic heterostructures could be used as potent photothermal agents for NIR-II photothermal antitumor treatment.

### *In vivo* NIR-II photothermal antitumor performance

**Figure 7:**
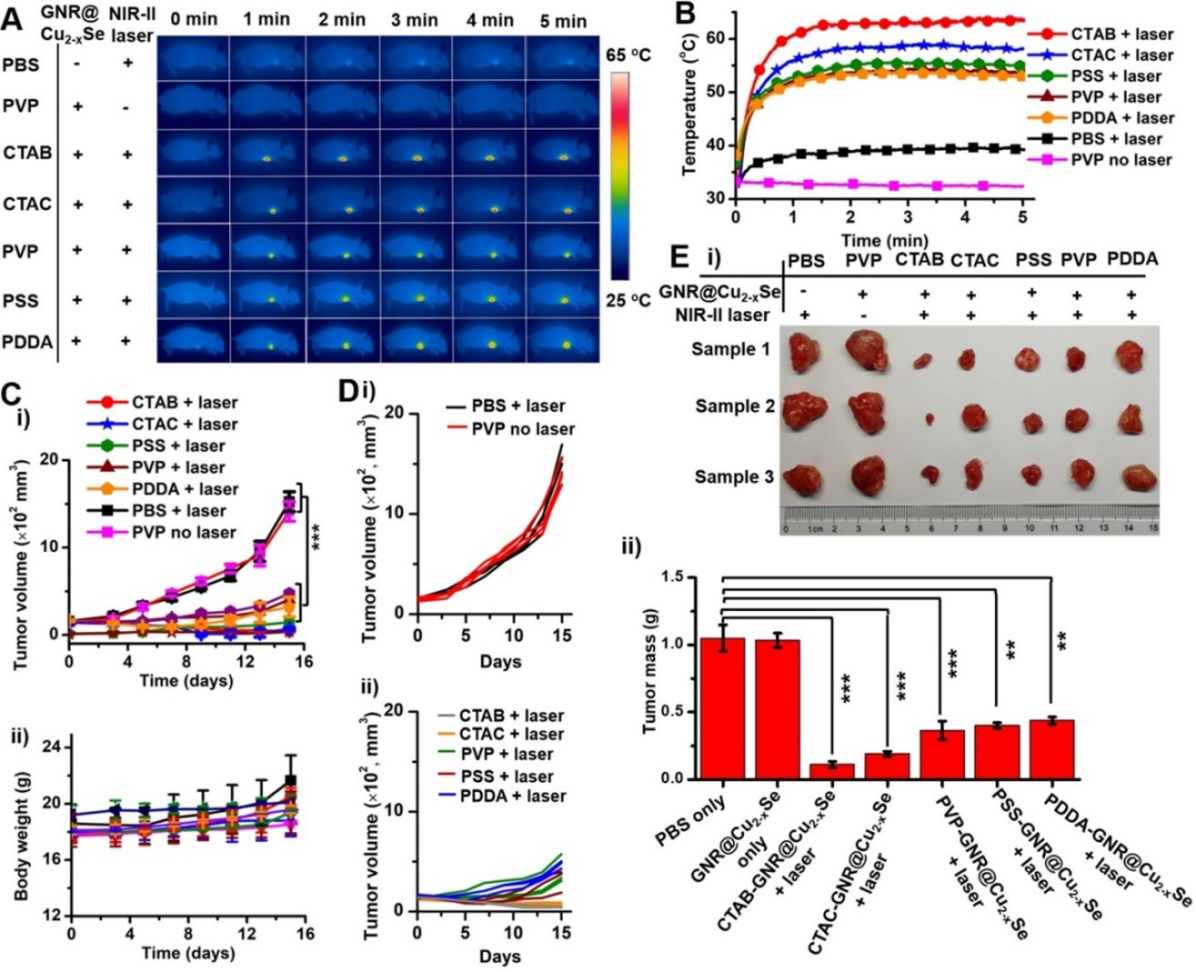
***In vivo* NIR-II photothermal antitumor performance**. (A) Infrared photothermal images at various time intervals and (B) temperature rising profiles of tumor-bearing mice treated under the NIR-II laser (1 W/cm^2^) together with GNR@Cu_2-x_Se structures prepared with CTAB, CTAC, PVP, PSS and PDDA, respectively. Control experiments were conducted with PBS + NIR-II laser (1 W/cm^2^) and GNR@Cu_2-x_Se structures (prepared with PVP) + NIR-II laser (1 W/cm^2^), respectively. (C) Change curves of (i) tumor volume and (ii) body weight of tumor-bearing mice with various treatments as mentioned above. (D) Individual tumor growth curves of the tumor-bearing mice shown in (C). (E) (i) Photographs and (ii) tumor mass of excised tumor tissues of the tumor-bearing mice examined at day 15 post-treatment with various GNR@Cu_2-x_Se heterostructures. Data were presented as mean ± standard deviation (SD), *P < 0.05, **P < 0.01, and ***P < 0.001.

Before performing evaluation of *in vivo* antitumor efficacy, we examined the hemocompatibility of the GNR@Cu_2-x_Se hybrids (Figure S40). Results show no observable hemolytic effect from the hybrids, indicating excellent hemocompatibility. Following this, analysis of the blood biochemistry of healthy mice intravenously administrated with GNR@Cu_2-x_Se hybrids (50 μL, 200 μg/mL) was performed to determine the levels of alkaline phosphatase (ALP), alanine transaminase (ALT), aspartate aminotransferase (AST), blood urea nitrogen (BUN), globulin (GLB) and creatinine (CREA) (Figure S41). All these levels in GNR@Cu_2-x_Se hybrid treated mice are similar to the control mice, indicating no significant hepatic and kidney toxicity. Thus, we demonstrate that GNR@Cu_2-x_Se hybrids have no observable systemic toxicity. To further assess the antitumor effects of these dual-plasmonic GNR@Cu_2-x_Se heterostructures, breast tumor-bearing mice model was established by subcutaneous injection of breast cancer MDA-MB-231 cells into the right frank region. When the tumors grew to ~150 mm^3^, suspensions of GNR@Cu_2-x_Se heterostructures (50 μL, 200 μg/mL for gold) were intratumorally injected into these tumor-bearing mice (n = 3), followed by irradiation with the NIR-II laser (1 W/cm^2^) for 5 min at 2 h post-injection. Control experiments were conducted with saline (50 μL) + NIR-II laser and GNR@Cu_2-x_Se hybrids only (50 μL, 200 μg/mL for gold), respectively. During the NIR-II laser irradiation, the full-body photothermal images and real-time tumor temperature were monitored by an infrared thermal camera (Figure 7A,B). The tumor temperature sharply rises to more than 53 ºC under the NIR-II laser irradiation for 5 min; the structures prepared with CTAB exhibit the highest steady-state temperature (63.6 ºC) compared with those with CTAC (58.2 ºC), PVP (53.6 ºC), PSS (55.1 ºC) and PDDA (53.0 ºC), respectively, all of which are significantly higher than PBS + NIR-II laser (39.4 ^o^C) and GNR@Cu_2-x_Se hybrids only (32.4 ºC). The temperature difference between those GRN@Cu_2-x_Se structures is due to the distinct Cu_2-x_Se percentage in these hybrids (Table 1). The relatively high tumor temperature induced by these hybrids under the NIR-II laser irradiation is enough to destroy tumor cells. The tumor volume and body weight of mice were monitored every other day after various treatments (Figure 7C,D and Figure S42). As seen, no significant tumor growth suppression was observed for tumor-bearing mice treated with either PBS + NIR-II laser or GNR@Cu_2-x_Se hybrids only. The tumors of mice were harvested and weighted at day 15 post-treatment (Figure 7E). It can be visually seen that the tumor growth is significantly inhibited by the treatment together with GNR@Cu_2-x_Se heterostructures and NIR-II laser, while individual treatments with PBS + NIR-II laser or GNR@Cu_2-x_Se structures only was unable to prevent the tumor growth. We can clearly see that the NIR-II laser irradiation causes obvious bruises and scars on tumor-bearing mice injected with GNR@Cu_2-x_Se heterostructures (Figures S43 and S44). Afterwards, the tumors gradually shrink, and the scars gradually vanish, indicating the tumor growth inhibition. We further performed the hematoxylin-eosin (H&E) staining analysis of tumor slices from tumor-bearing mice at day 15 post-treatment (Figure S44). The H&E staining images show severe tumor necrosis in tumor-bearing mice treated with all GNR@Cu_2-x_Se structures under the NIR-II irradiation, but no obvious damage to the tumors treated with either PBS + NIR-II laser or GNR@Cu_2-x_Se structures only. It is worth noting that the relative less tumor necrosis induced by the GNR@Cu_2-x_Se heterostructures under the NIR-II laser from PDDA-modified GNRs is due to the low Cu_2-x_Se percentage. These observations are in good agreement with the significant tumor growth inhibition with treatments by the GNR@Cu_2-x_Se heterostructures under the NIR-II laser irradiation aforementioned. Thus, these findings clearly demonstrate the superior *in vivo* photothermal antitumor performance of these dual-plasmonic GNR@Cu_2-x_Se heterostructures in the NIR-II biowindow.

## Conclusion

In summary, we have reported the capping agent-mediated regioselective overgrowth of Cu_2-x_Se on the GNRs to prepare dual-plasmonic GNR@Cu_2-x_Se heterostructures with tunable NIR-II plasmon absorption by a Se template method. The synthesis involves - (i) the initial deposition of the amorphous Se template *via* the reduction of SeO_2_ by the AA reducing agent, and (ii) then the following conversion into Cu_2-x_Se. We demonstrated the exclusive growth of Cu_2-x_Se on the lateral one-side and two-sides of the GNRs using CTAC and CTAB capping agents, respectively; conformal coating on the GNRs proceeded to form the core-shell GNR@Cu_2-x_Se structures with either neutral PVP or anionic PSS, while island growth of Cu_2-x_Se on the GNRs was observed with PDDA. This side-controllable deposition, conformal coating and island growth were clearly revealed by monitoring the SeO_2_ concentration-dependent morphological evolution. The regioselective deposition mediated by surface chemistry produced dual-plasmonic GNR@Cu_2-x_Se hybrids with tunable plasmon bands in both wavelength and intensity in the NIR-II biowindow ranging from 1050-1250 nm. Experimental results combined with numerical simulations proved the outstanding photothermal performance with the photothermal conversion efficiency up to 58-85% and high photostability under the NIR-II laser, much better than both individual GNRs and Cu_2-x_Se NCs due to the strong plasmonic coupling effects. We for the first time find that the photothermal conversion efficiency is closely dependent on the proportion of optical absorption converted into heat of the incident light but not linearly related to the fraction of constituents in the hybrids; however, the temperature increase is tightly related to the concentration of the constituents. We further proved that these dual-plasmonic GNR@Cu_2-x_Se heterostructures could be employed for both *in vitro* and *in vivo* photothermal ablation of cancer cells under the NIR-II laser. A combined treatment with GNR@Cu_2-x_Se heterostructures and NIR-II laser significantly inhibits the tumor growth. These hybrids present high optical absorption coefficient, high photothermal conversion efficiency, excellent stability, and so on, which is much superior to these inorganic and small organic NIR photothermal materials 61-63. Therefore, the present work provides a facile strategy for the spatially confined deposition of Cu_2-x_Se on the GNRs for dual-plasmonic GNR@Cu_2-x_Se heterostructures, and demonstrates their promising potential in the clinic as photothermal agents for photothermal ablation of tumors. We must point out that the exclusive deposition of Cu_2-x_Se on the one- or two-ends of GNRs has not been achieved by the present method, which are currently being pursued in our laboratory. Also, this study intratumorally administrates GNR@Cu_2-x_Se heterostructures to evaluate the *in vivo* photothermal performance in this work, so that almost all GNR@Cu_2-x_Se heterostructures stay in tumors. In addition to the photothermal ablation application, dual-plasmonic GNR@Cu_2-x_Se heterostructures could present potential multifunctionalities for biomedical applications, including surface-enhanced Raman spectroscopy, photoacoustic imaging, computed tomography and photodynamic therapy. We are currently working on developing all-in-one NIR-II theranostic platforms using these dual-plasmonic materials for multimodality detection-guided *in vivo* combined therapy of cancer, which allows us to systematically investigate *in vivo* biosafety in the future.

## Supplementary Material

Supplementary figures and tables.

## Figures and Tables

**Table 1:** Weight percentage of Cu_2-x_Se in GNR@Cu_2-x_Se hybrids prepared with various SeO_2_ concentrations using different capping agents

[SeO_2_] (mM)	GNR@Cu_2-x_Se heterostructures				
	CTAB-	CTAC-	PVP-	PSS-	PDDA-
0.28	36.6%	42.4%	32.2%	35.7%	39.6%
0.56	55.3%	55.1%	45.3%	53.2%	51.2%
1.12	65.0%	70.4%	65.5%	67.6%	69.7%
