# Supplementary material for: Rationally designed dual-plasmonic gold nanorod@cuprous selenide hybrid heterostructures by regioselective overgrowth for in vivo photothermal tumor ablation in the second near-infrared biowindow
Source: Theranostics. 2020 Sep 19;10(25):11656–72. doi: 10.7150/thno.51287 (PMC7546011; doi:10.7150/thno.51287)
Supplement: Supplementary file 1 — Supplementary figures and tables. [file thnov10p11656s1.pdf]

Supplementary data

Rationally designed dual-plasmonic gold nanorod@cuprous selenide hybrid heterostructures by regioselective overgrowth for *in vivo* photothermal tumor ablation in the second near-infrared biowindow

Beibei Shan, Haitao Wang, Linhu Li, Guangzhi Zhou, Yu Wen, Mingyang Chen, and Ming Li*

School of Materials Science and Engineering, Central South University, Changsha, Hunan 410083, China

*To whom the correspondence should be addressed. E-mail: liming0823@csu.edu.cn and liming0823@gmail.com

S1. Calculation of lattice mismatch between face-centered cubic gold domain and cubic berzelianite $Cu_{2-x}Se$ domain in GNR@$Cu_{2-x}Se$ heterostructures.

The lattice mismatch between the FCC gold domain and the cubic berzelianite $Cu_{2-x}Se$ domain in the GNR@$Cu_{2-x}Se$ heterostructures can be calculated on the basis of the conventional definition using the following formula:

$$lattice\ mismatch\ (\%) = \frac{lattice\ parameter\ of\ Cu_{2-x}Se - lattice\ parameter\ of\ gold}{lattice\ parameter\ of\ gold} \times 100$$

Table S1. Lattice mismatch between the FFC gold domain and the cubic berzelianite $Cu_{2-x}Se$ domain in the GNR@$Cu_{2-x}Se$ heterostructures.

Crystal plane of $Cu_{2-x}Se$	(111)	(111)	(200)	(200)	(220)	(220)	(220)	(311)
Crystal plane of gold	(111)	(220)	(111)	(220)	(111)	(220)	(311)	(111)
Lattice mismatch (%)	41	62	22	99	16	41	65	36
Crystal plane of $Cu_{2-x}Se$	(311)	(311)	(311)					
Crystal plane of gold	(220)	(311)	(400)					
Lattice mismatch (%)	20	41	70					

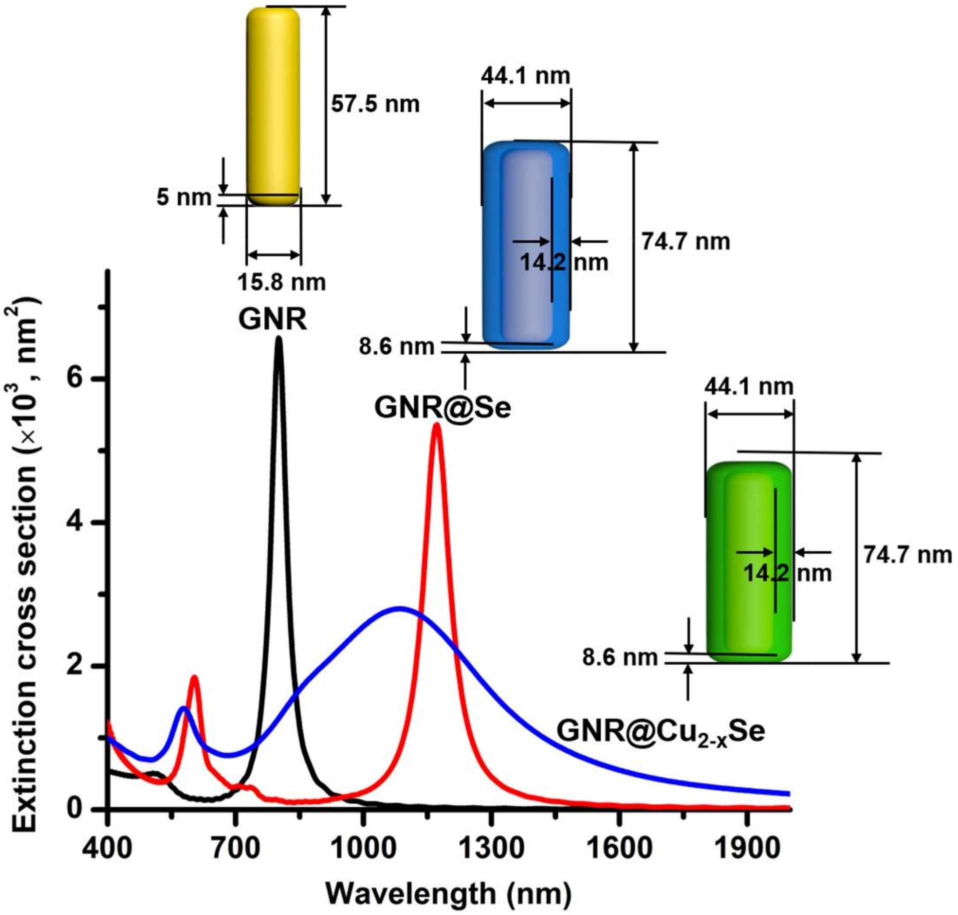

Figure S1. FDTD-calculated optical extinction spectra of GNRs, core-shell GNR@Se structures and core-shell GNR@$Cu_{2-x}Se$ structures. The models for FDTD simulations are shown as well. The dimensional parameters (length × width) of GNRs are adopted from the statistical results shown in Figure S1. The thickness of shell of core-shell GNR@Se structures and GNR@$Cu_{2-x}Se$ heterostructures is taken from GNR@$Cu_{2-x}Se$ structures prepared with PVP and 1.12 mM SeO_2.

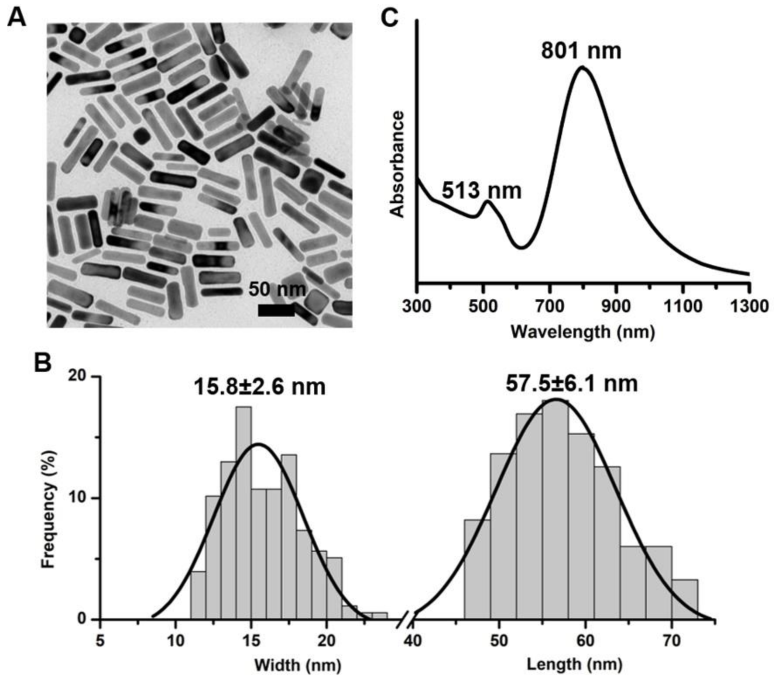

Figure S2. Structure and optical properties of GNRs. (A) TEM image, (B) size distribution and (C) optical absorption spectrum in water of GNRs. The length and width of GNRs were statistically measured from the TEM image, with at least 150 nanoparticles counted. The GNRs have an average length of 57.5 ± 6.1 nm and width of 15.8 ± 2.6 nm, and display the transverse plasmon band at 513 nm and longitudinal plasmon band at 801 nm.

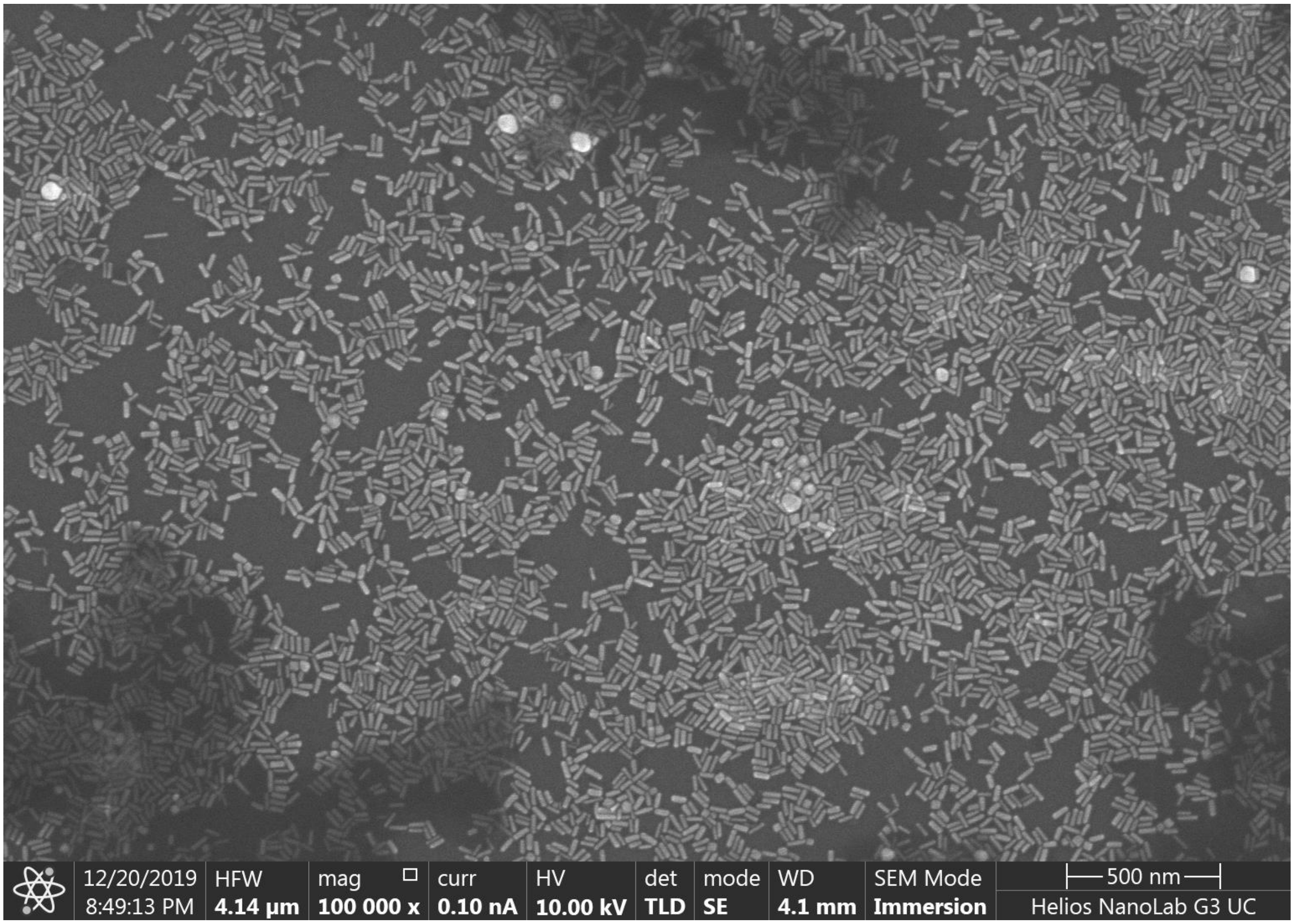

Figure S3. Large scale SEM image of as-prepared GNRs by the seed-mediated growth method.

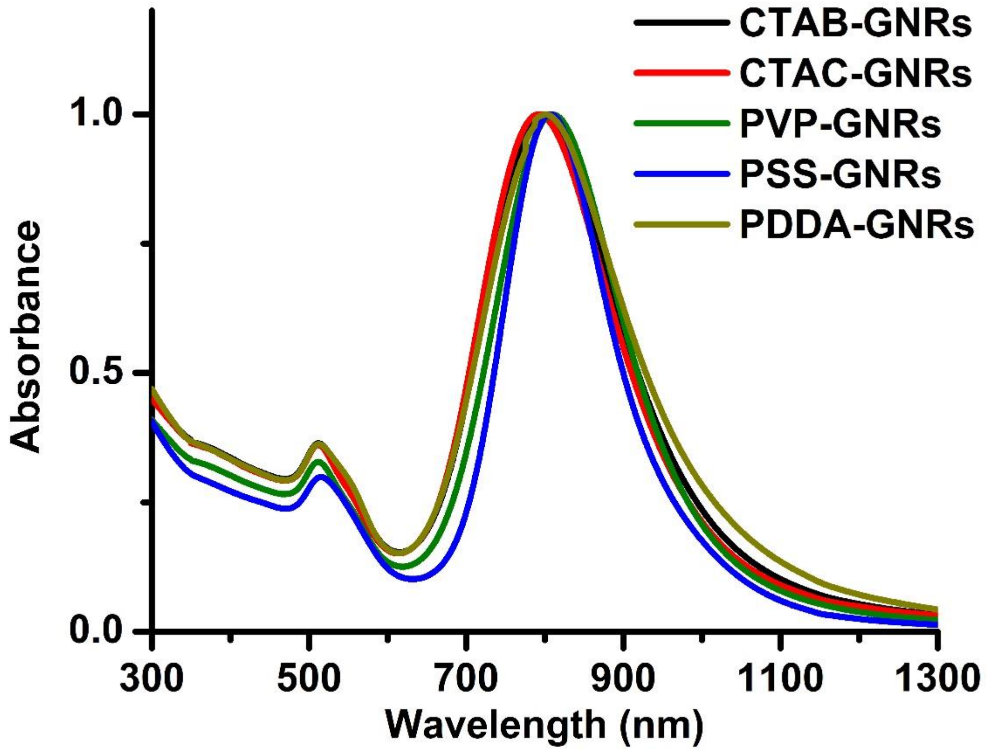

Figure S4. Optical absorption spectra of aqueous suspensions of GNRs modified with CTAB, CTAC, PVP, PSS and PDDA capping agents, respectively. Negligible spectral change in all these capping agent-modified GNRs was observed, indicating no significant aggregation.

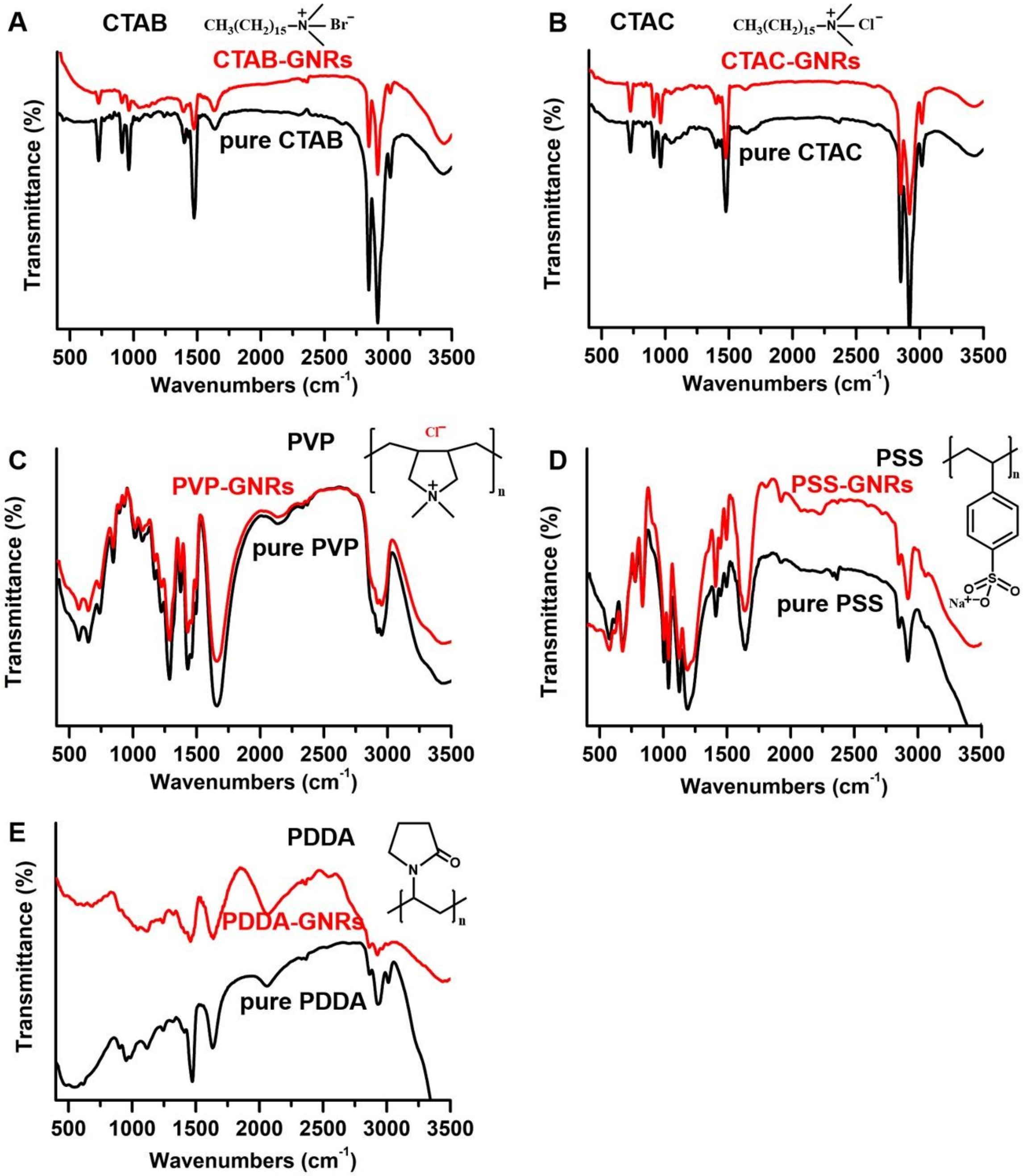

Figure S5. FTIR spectra of (A) CTAB-, (B) CTAC-, (C) PVP-, (D) PSS- and (E) PDDA-modified GNRs. CTAB-, CTAC-, PVP-, PSS- and PDDA-modified GNRs display FTIR bands characteristic of their capping agents, respectively, verifying the successful modification of GNRs.

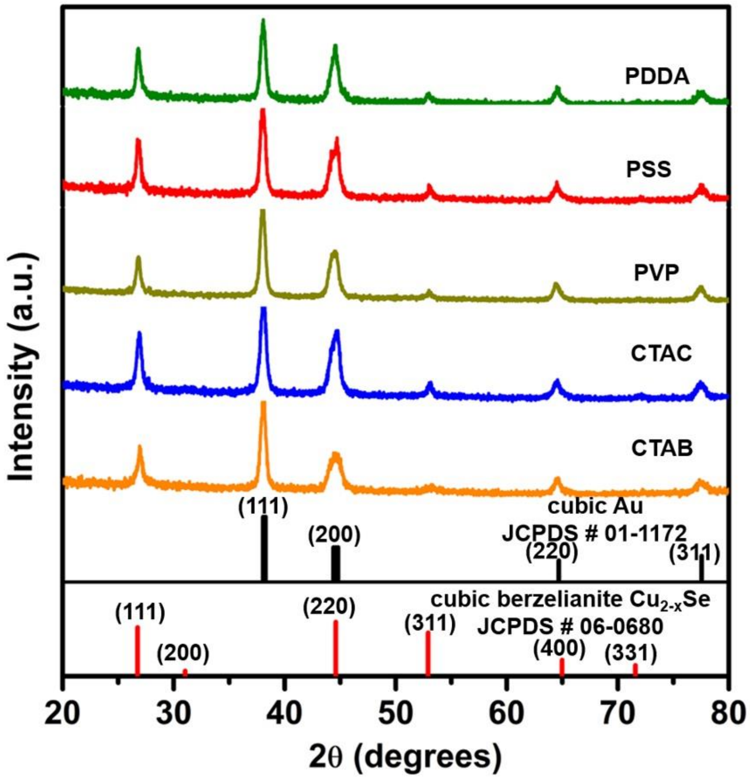

Figure S6. Powder XRD patterns of GNR@$Cu_{2-x}Se$ heterostructures prepared with CTAB, CTAC, PVP, PSS and PDDA, respectively. The standard XRD patterns of cubic gold (JCPDS # 01-1172) and cubic berzelianite $Cu_{2-x}Se$ (JCPDS # 06-0680) are shown for reference. All as-prepared GNR@$Cu_{2-x}Se$ heterostructures exhibits similar diffraction peaks, in which diffraction peaks at $2\theta=38.1^{o}$ and 77.5^{o} respectively assigned to the (111) and (311) planes of the face-centered cubic gold phase from the GNRs, these GNR@$Cu_{2-x}Se$ hybrids exhibit consistent XRD peaks at $2\theta=26.7^{o}$, 44.6^{o}, 52.9^{o} and 65.0^{o} all indexed to the (111), (220), (311) and (400) planes of the cubic berzelianite $Cu_{2-x}Se$ phase. XRD results confirmed that high crystalline GNR@$Cu_{2-x}Se$ nanostructures were prepared with various capping agents by the present Se template method.

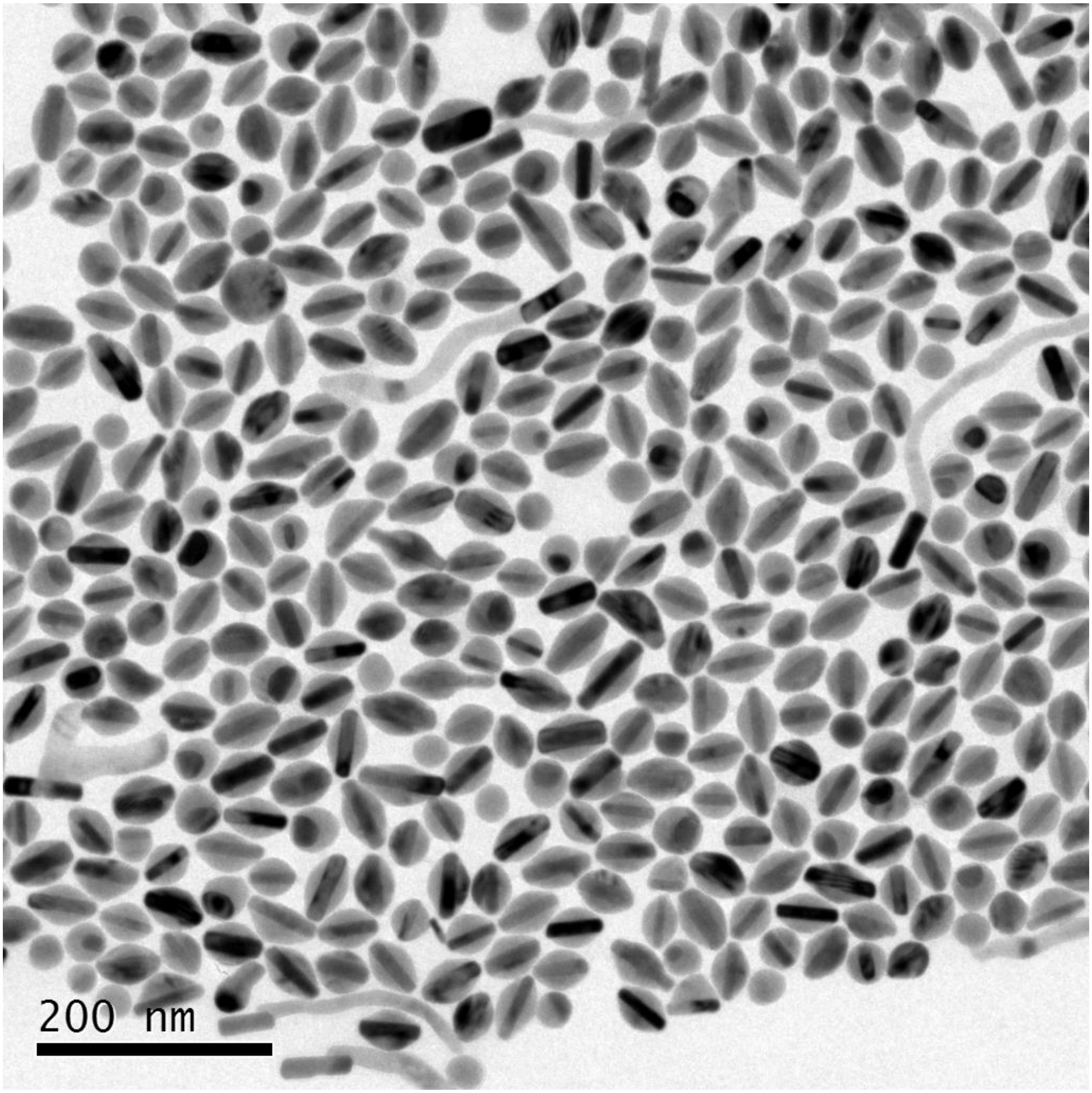

Figure S7. Large-area TEM image of GNR@$Cu_{2-x}Se$ heterostructures prepared with CTAB in the presence of 0.56 mM SeO_2.

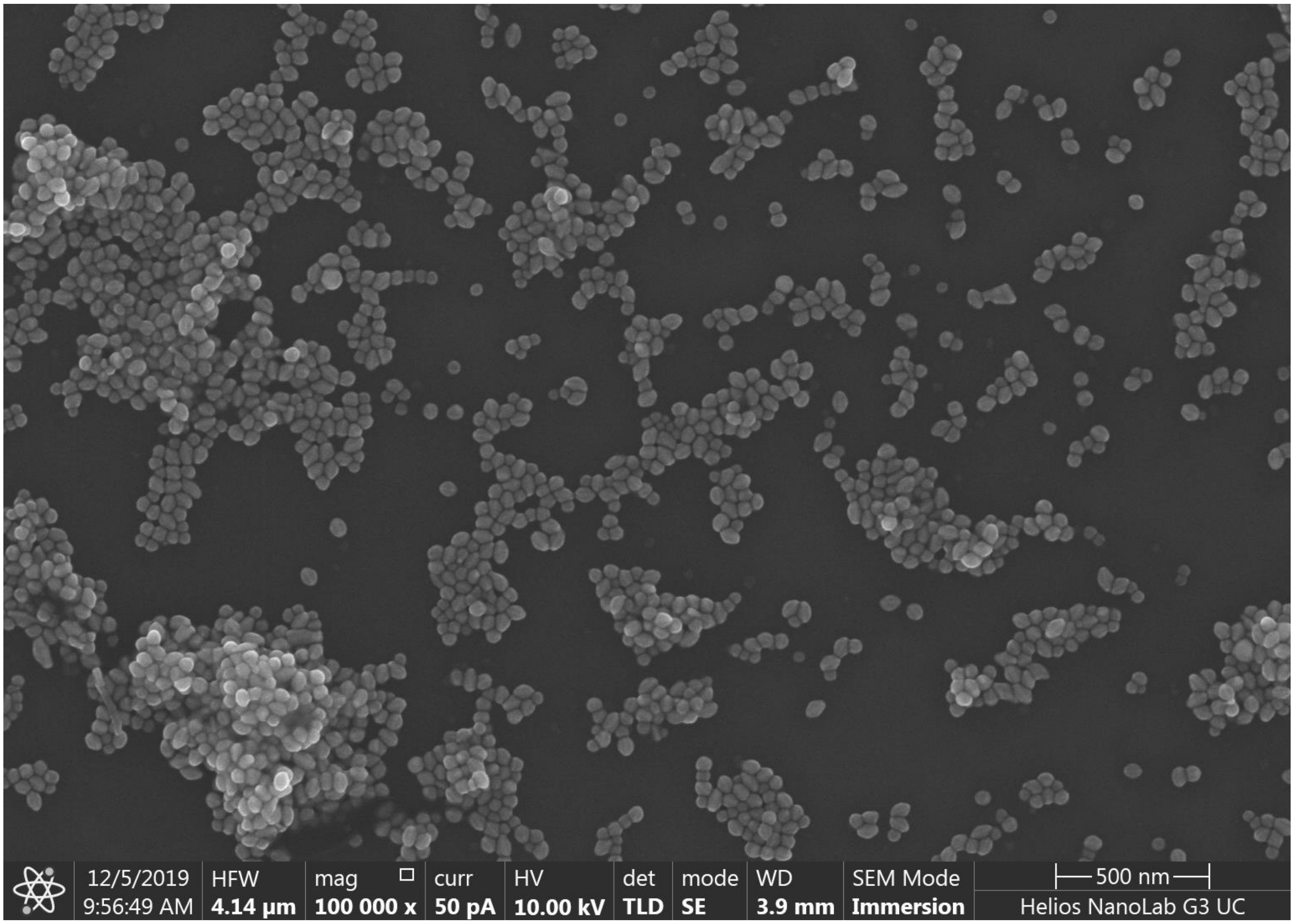

Figure S8. Large-area SEM image of GNR@$Cu_{2-x}Se$ heterostructures prepared with CTAB in the presence of 0.56 mM SeO_2.

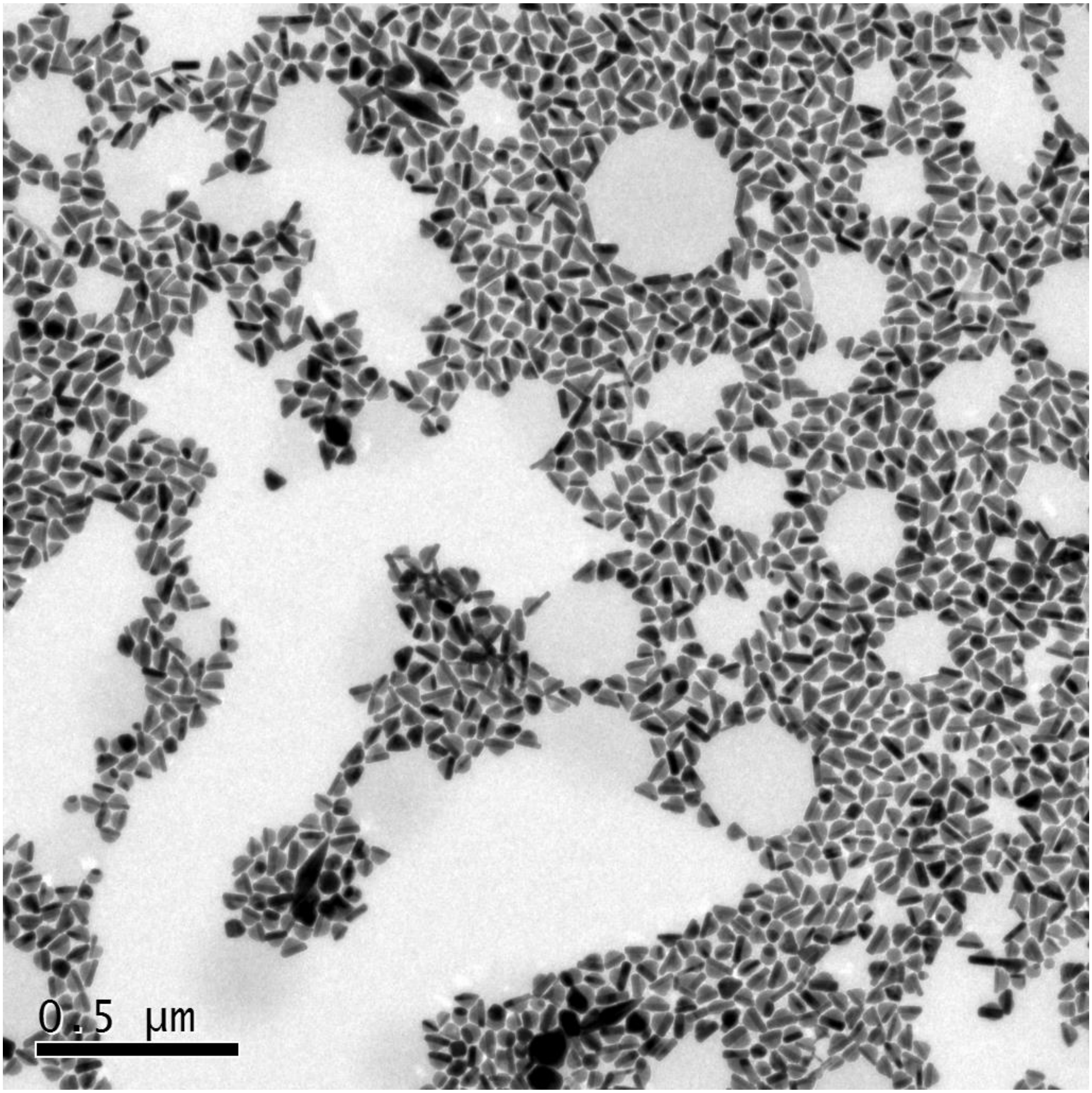

Figure S9. Large-area TEM image of GNR@$Cu_{2-x}Se$ heterostructures prepared with CTAC in the presence of 0.56 mM SeO_2.

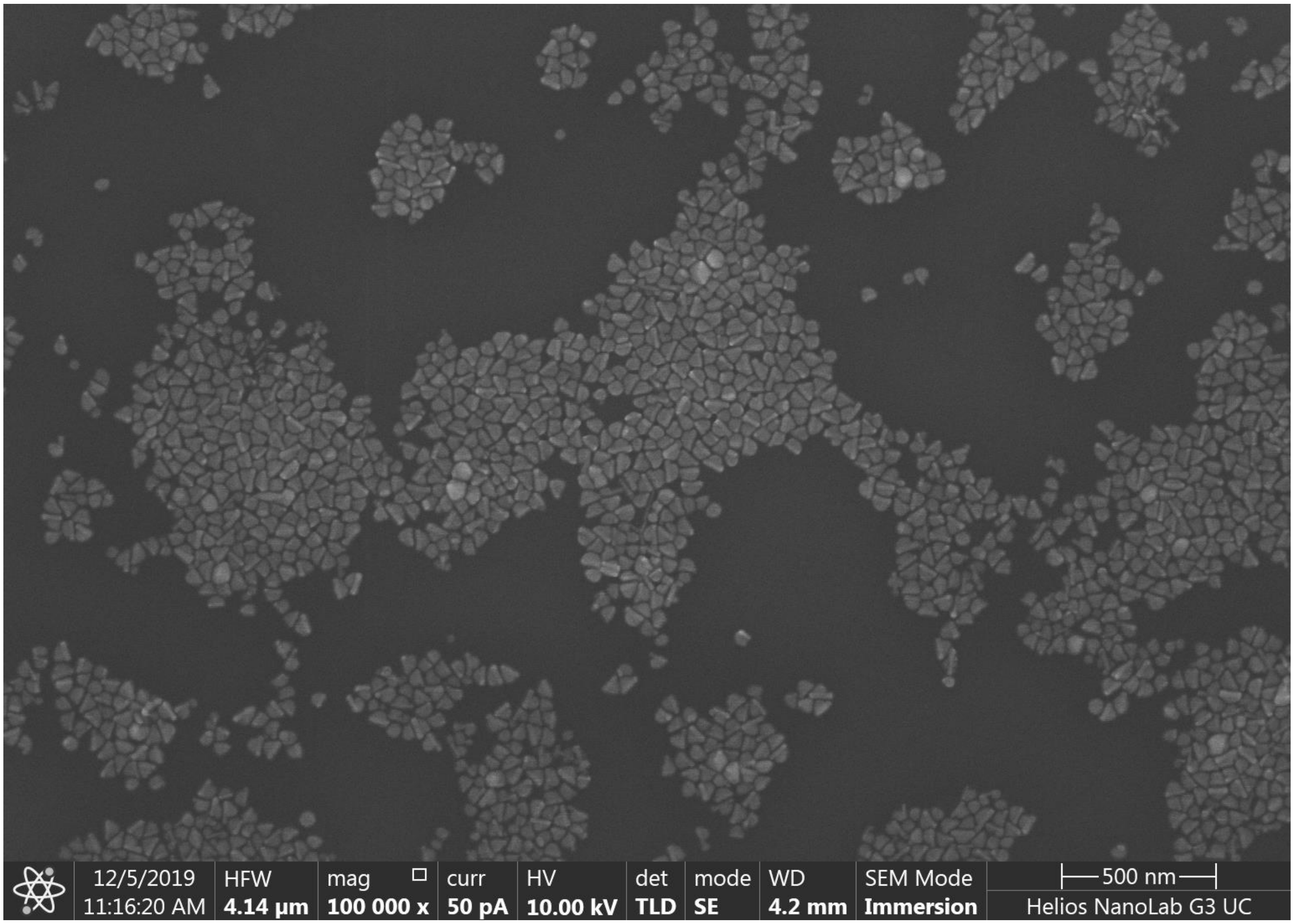

Figure S10. Large-area SEM image of GNR@$Cu_{2-x}Se$ heterostructures prepared with CTAC in the presence of 0.56 mM SeO_2.

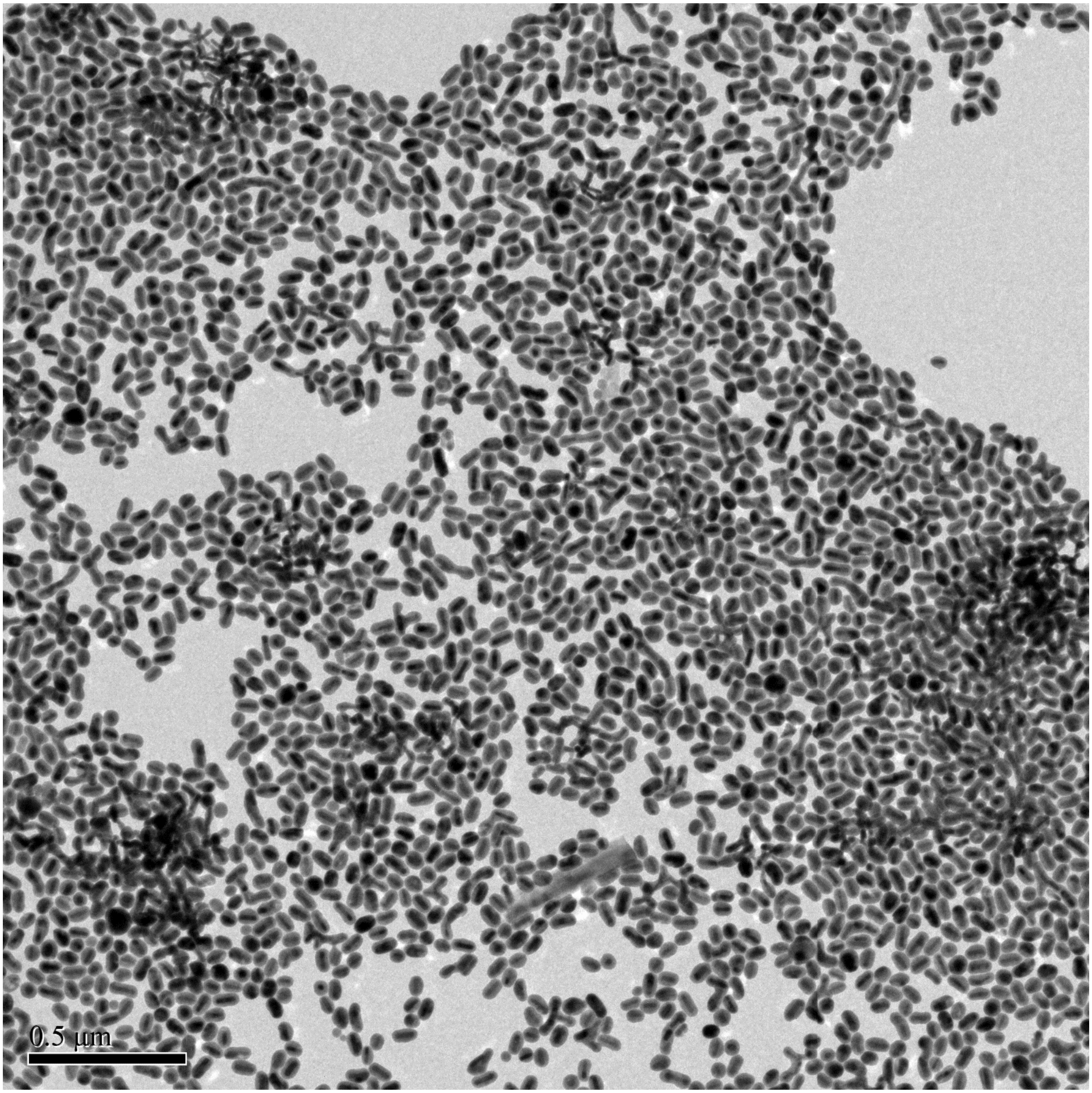

Figure S11. Large-area TEM image of GNR@$Cu_{2-x}Se$ heterostructures prepared with PVP in the presence of 0.56 mM SeO_2.

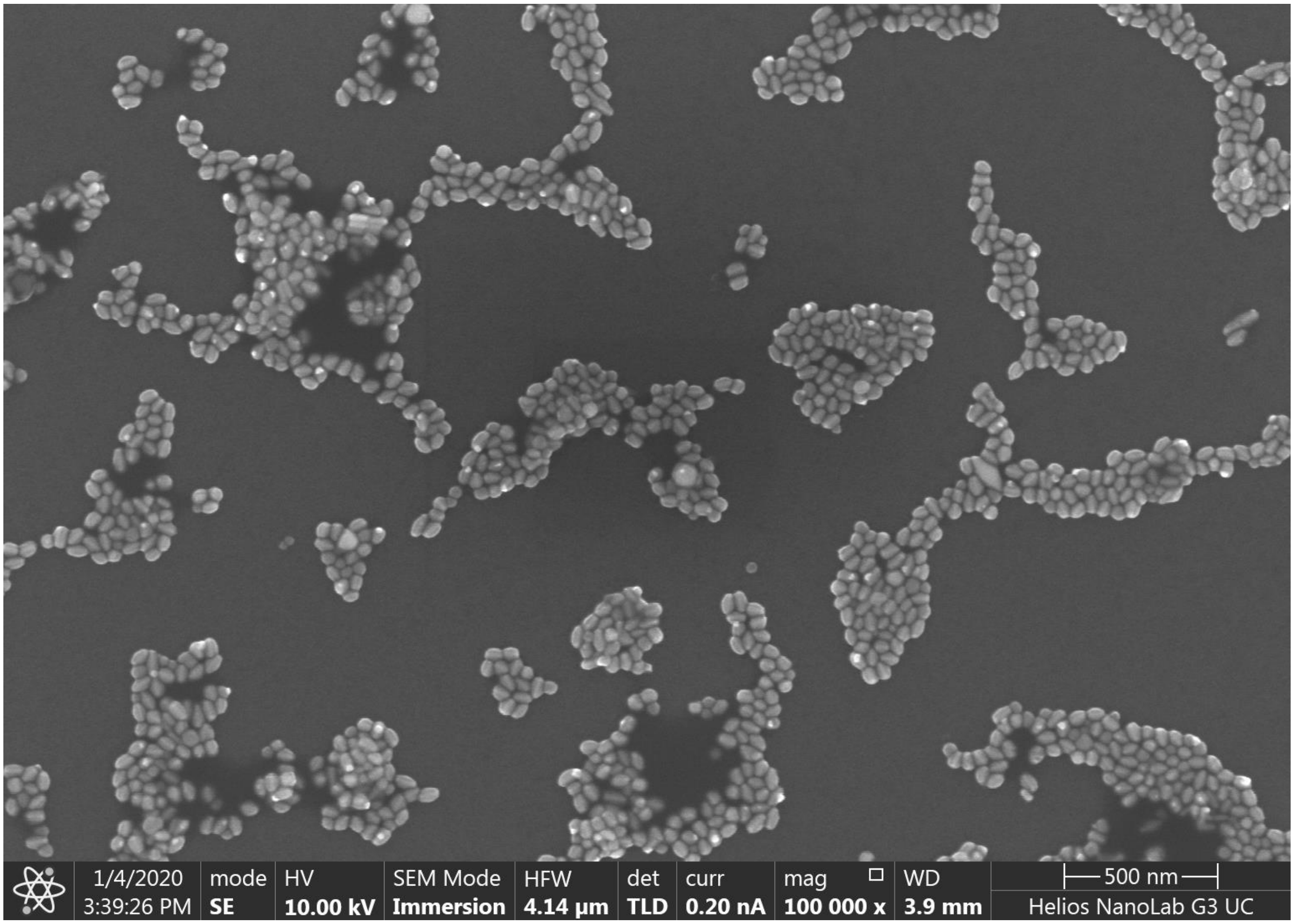

Figure S12. Large-area SEM image of GNR@$Cu_{2-x}Se$ heterostructures prepared with PVP in the presence of 0.56 mM SeO_2.

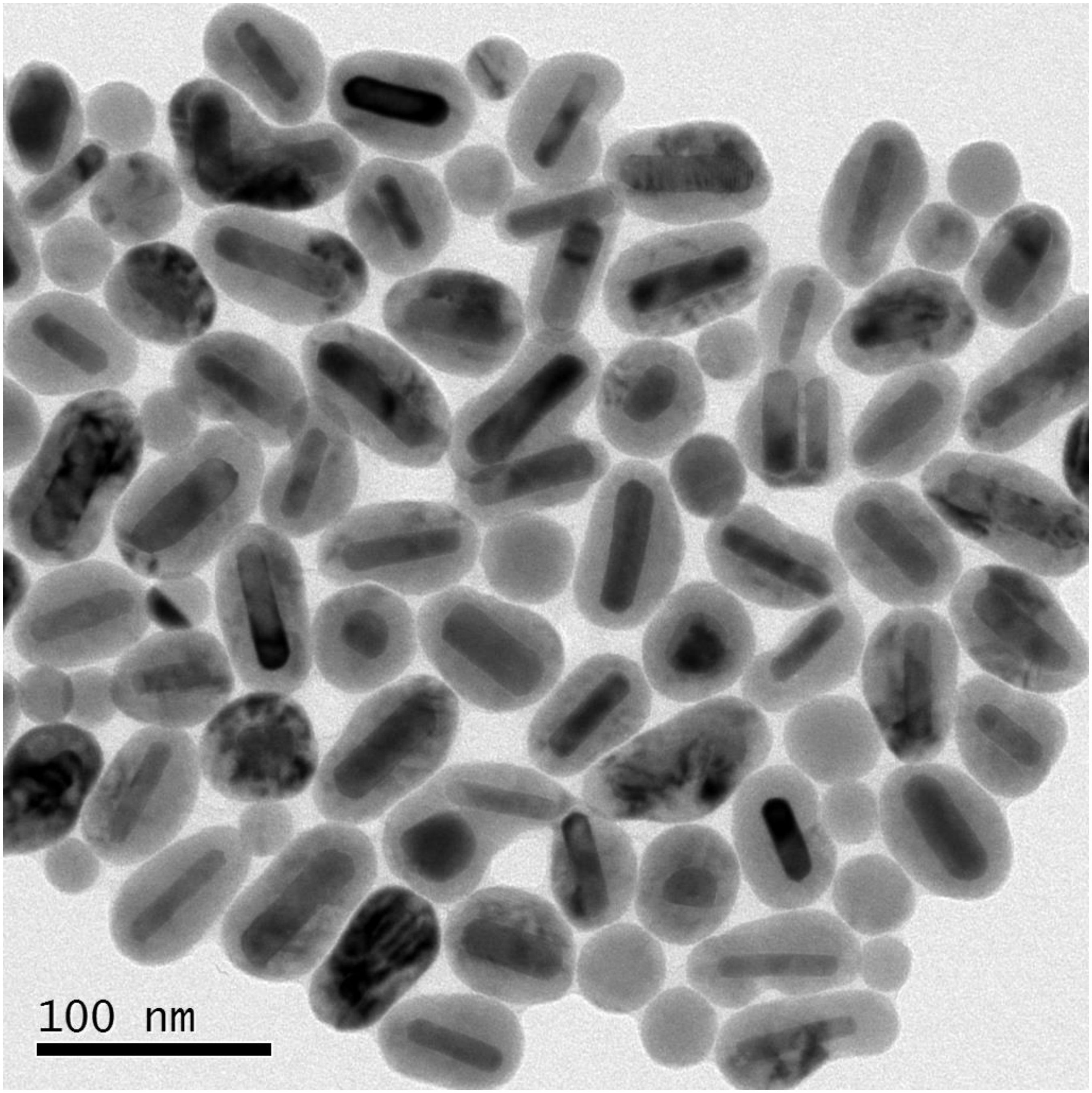

Figure S13. Large-area TEM image of GNR@$Cu_{2-x}Se$ heterostructures prepared with PSS in the presence of 0.56 mM SeO_2.

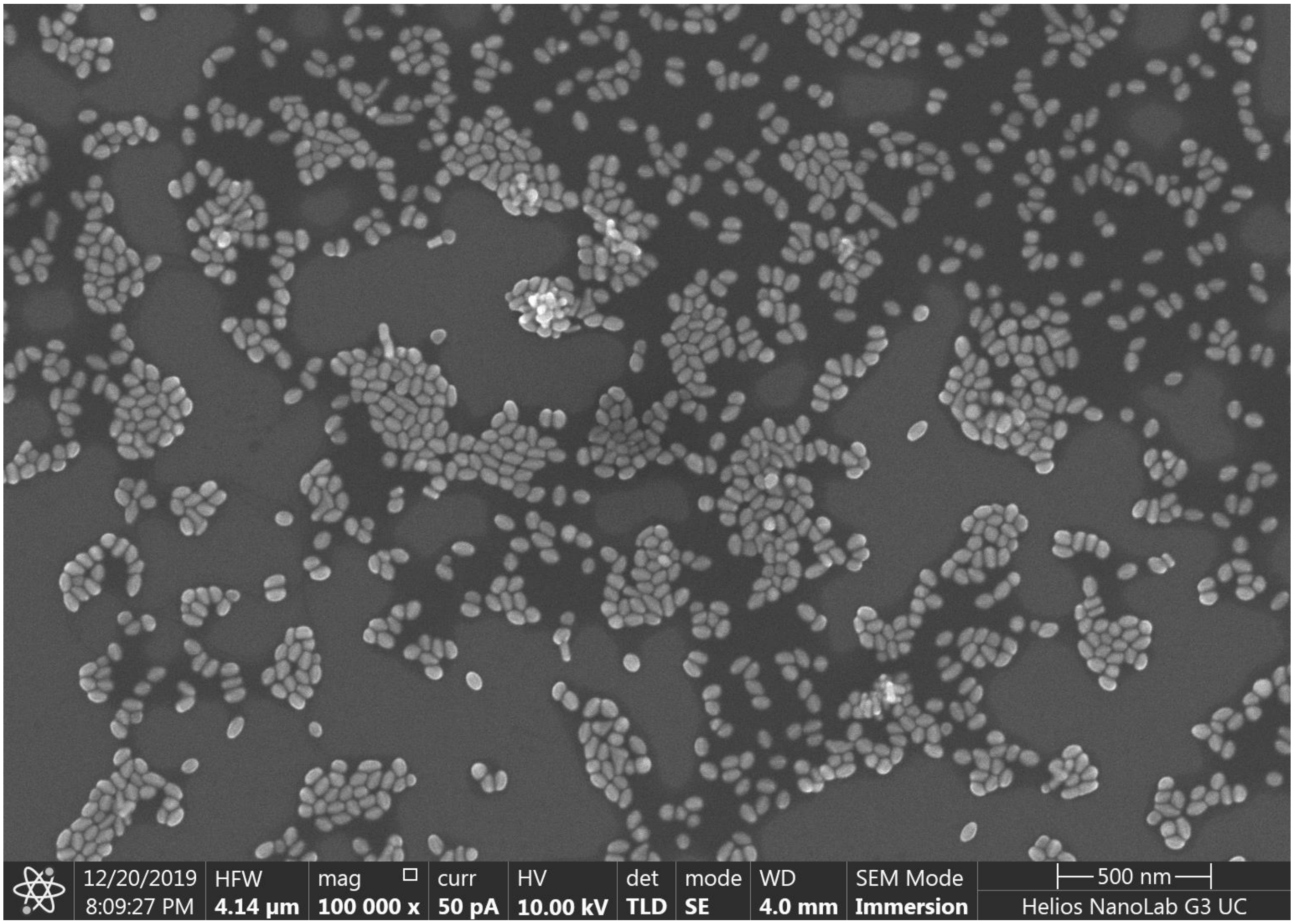

Figure S14. Large-area SEM image of GNR@$Cu_{2-x}Se$ heterostructures prepared with PSS in the presence of 0.56 mM SeO_2.

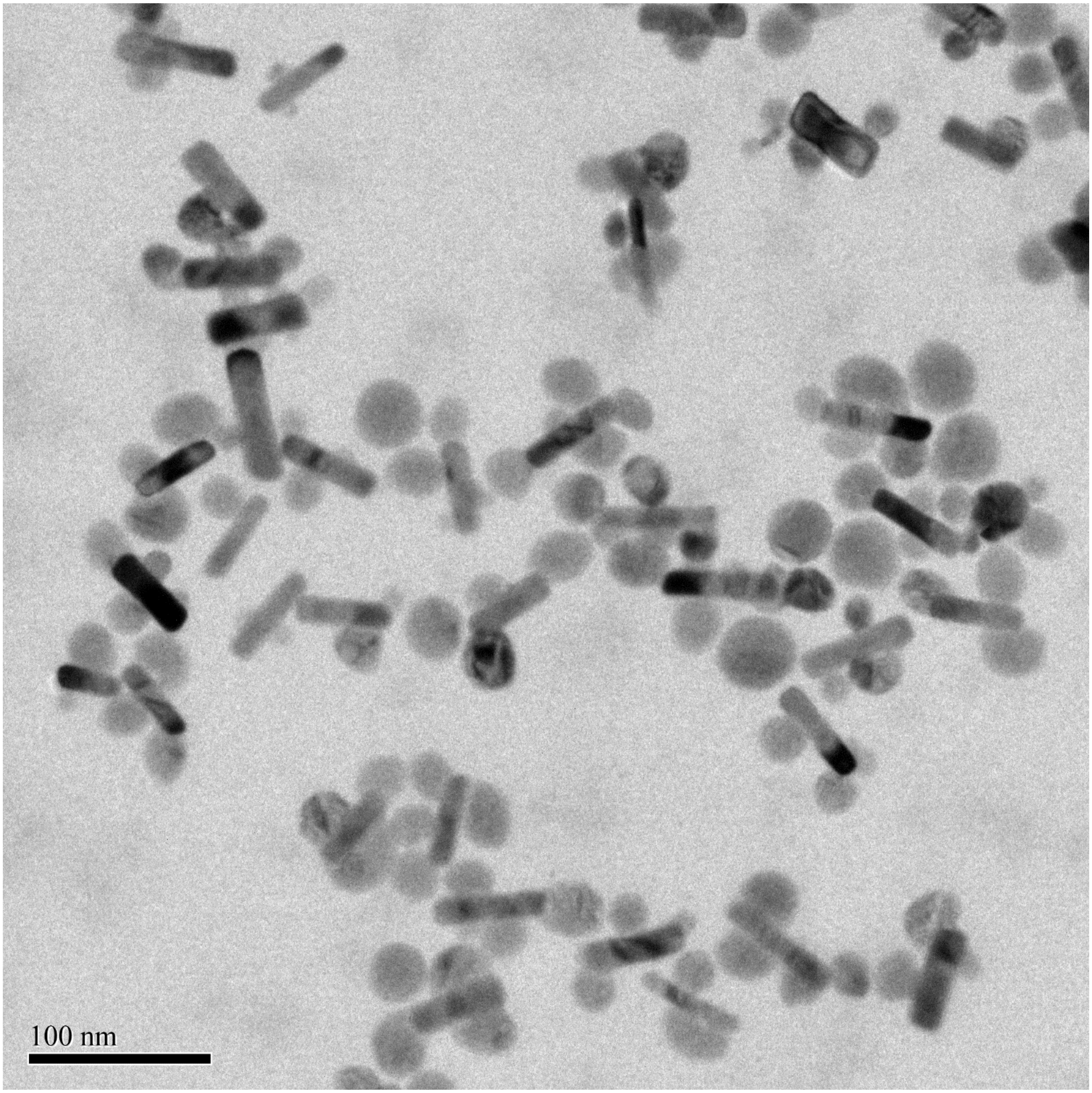

Figure S15. Large-area TEM image of GNR@$Cu_{2-x}Se$ heterostructures prepared with PDDA in the presence of 0.56 mM SeO_2.

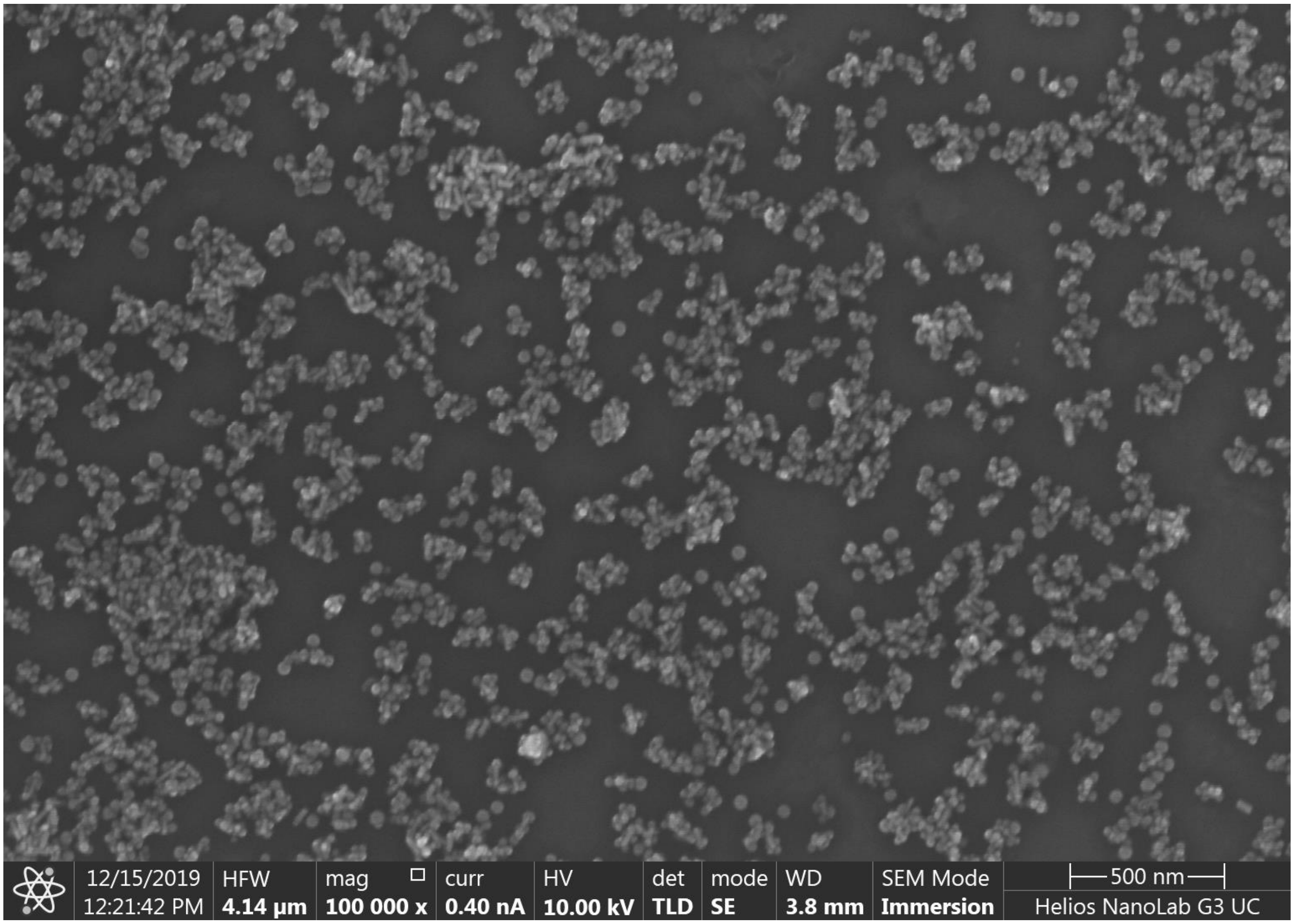

Figure S16. Large-area SEM image of GNR@$Cu_{2-x}Se$ heterostructures prepared with PDDA in the presence of 0.56 mM SeO_2.

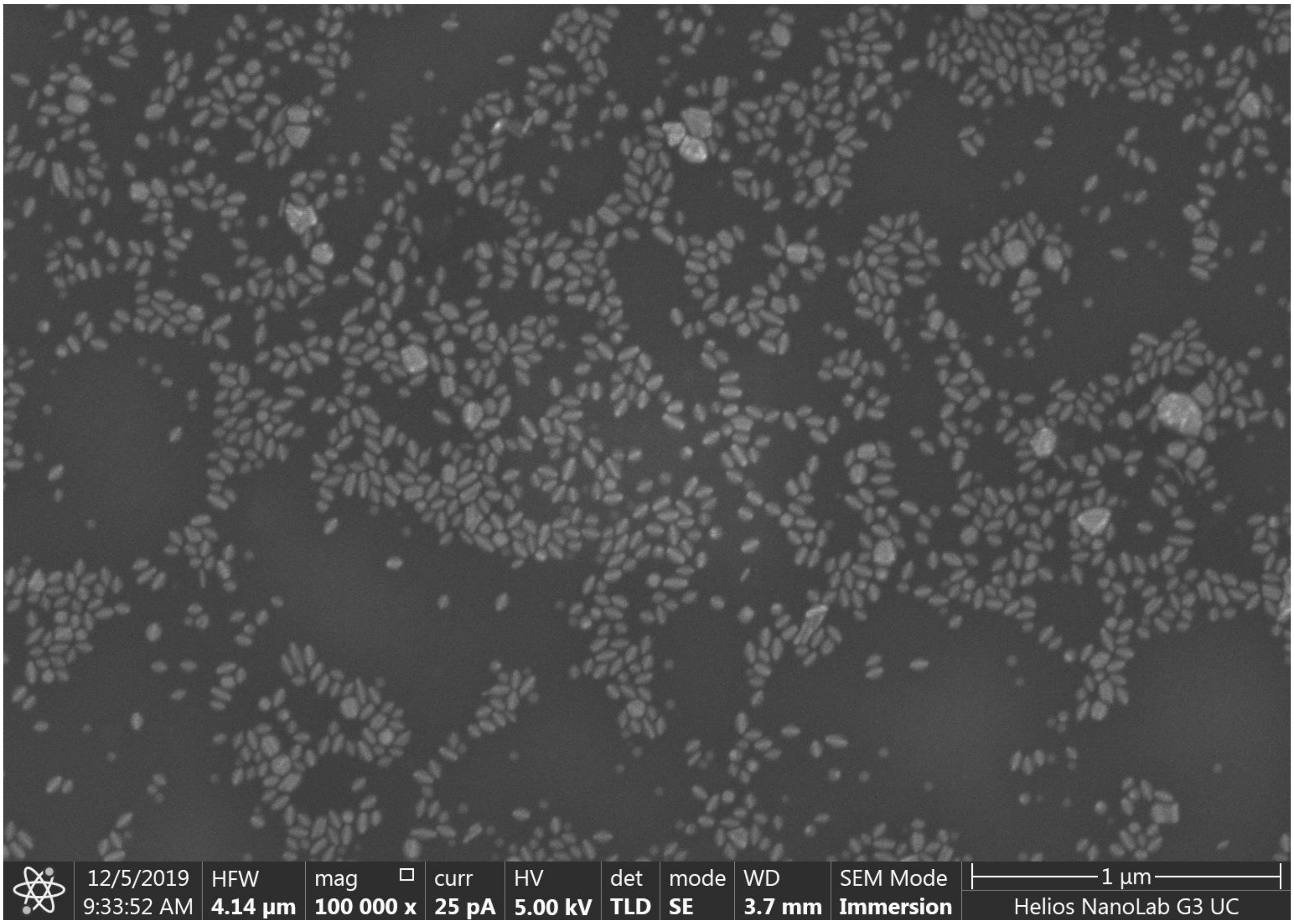

Figure S17. Large-area SEM image of GNR@Se hybrids prepared with CTAB in the presence of 0.56 mM SeO_2.

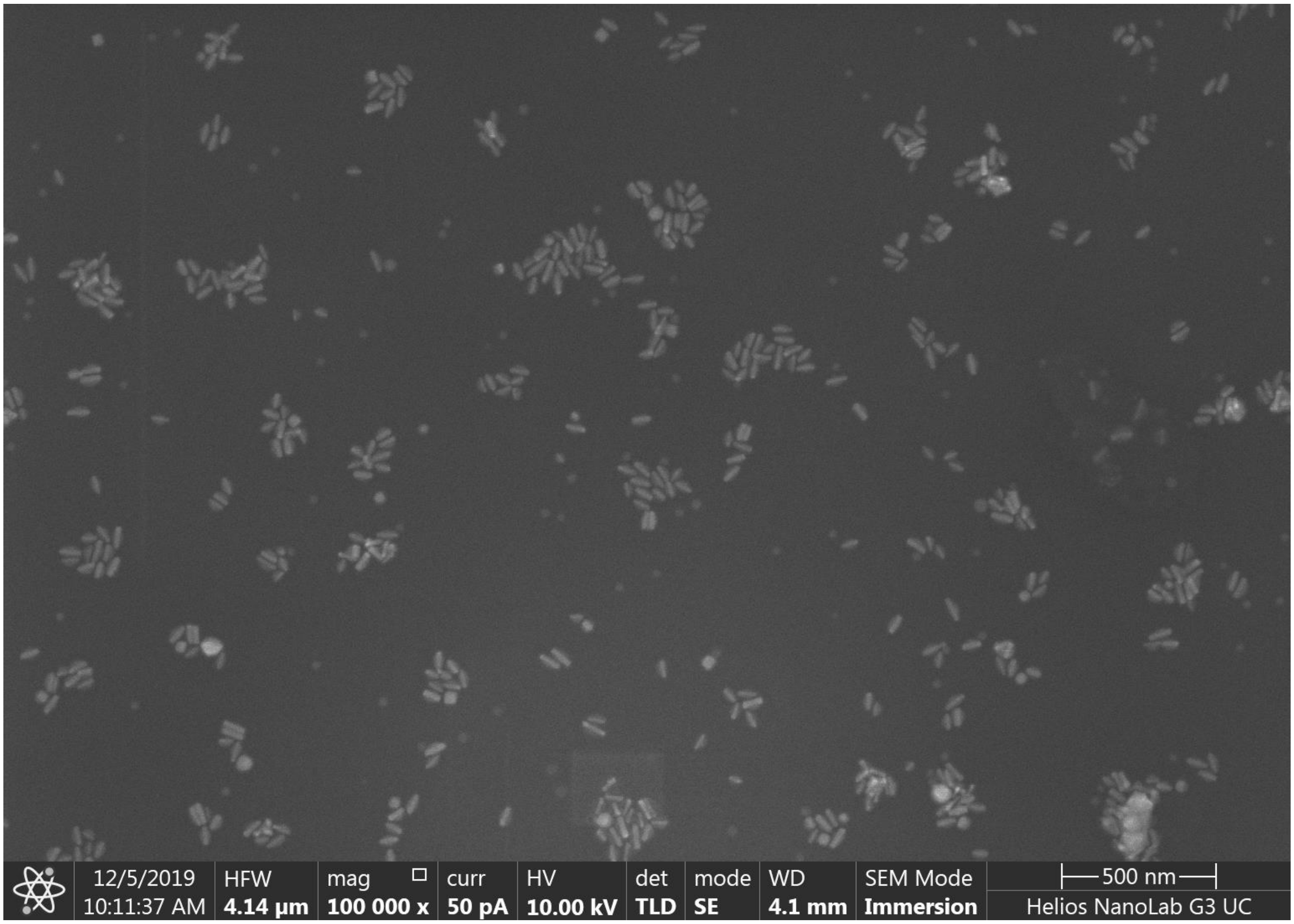

Figure S18. Large-area SEM image of GNR@Se hybrids prepared with CTAC in the presence of 0.56 mM SeO_2.

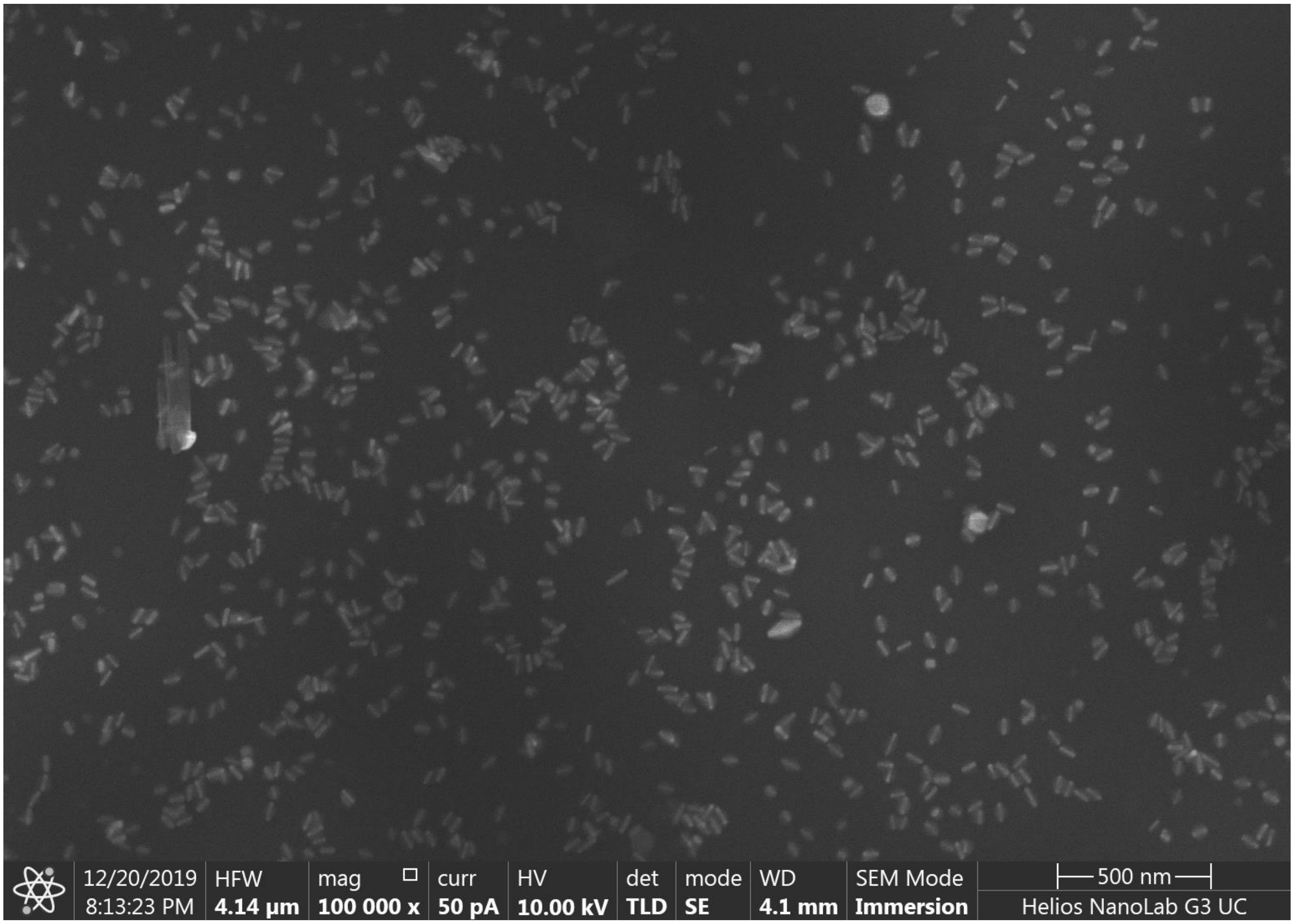

Figure S19. Large-area SEM image of GNR@Se hybrids prepared with PVP in the presence of 0.56 mM SeO_2.

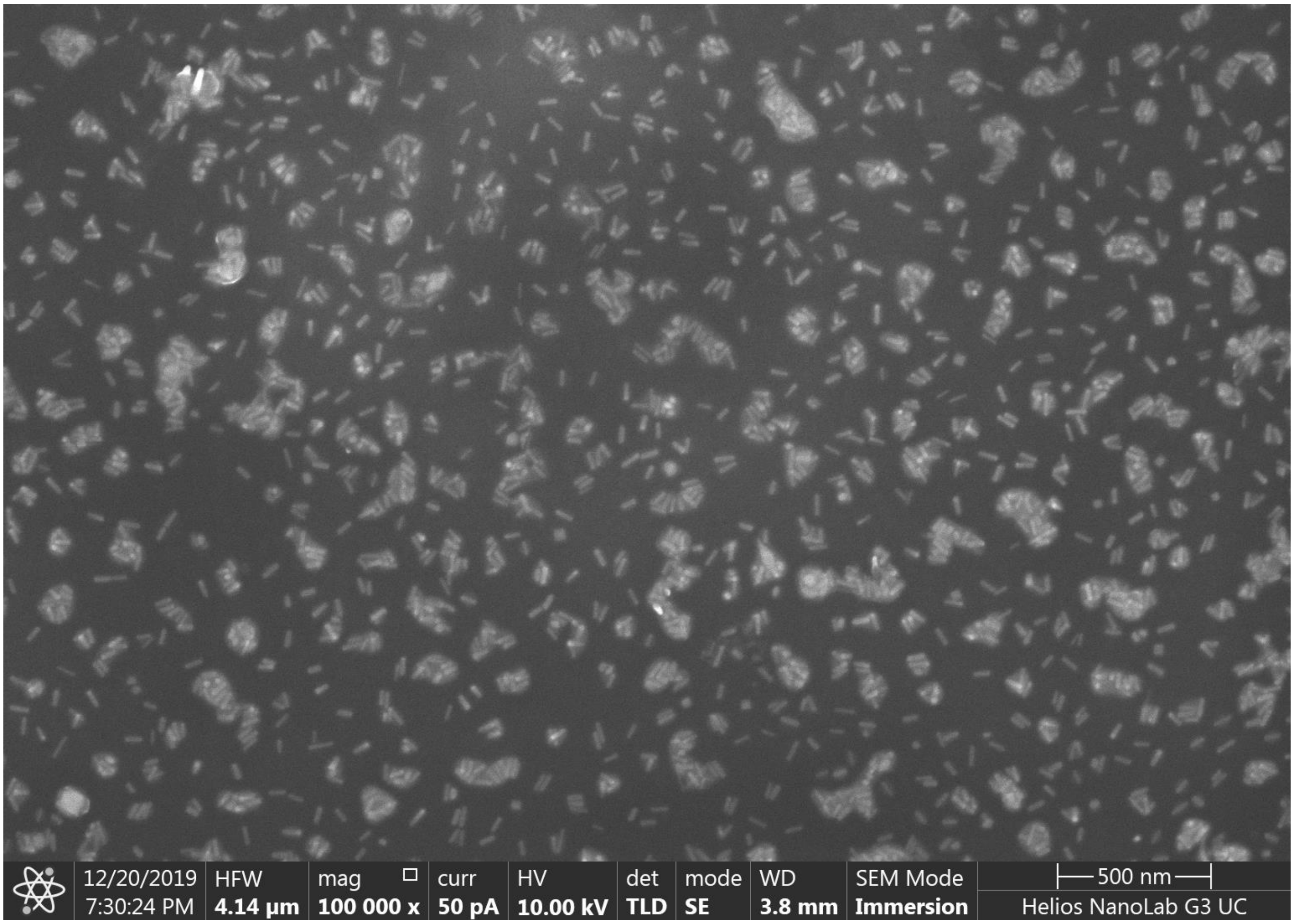

Figure S20. Large-area SEM image of GNR@Se hybrids prepared with PSS in the presence of 0.56 mM SeO_2.

1

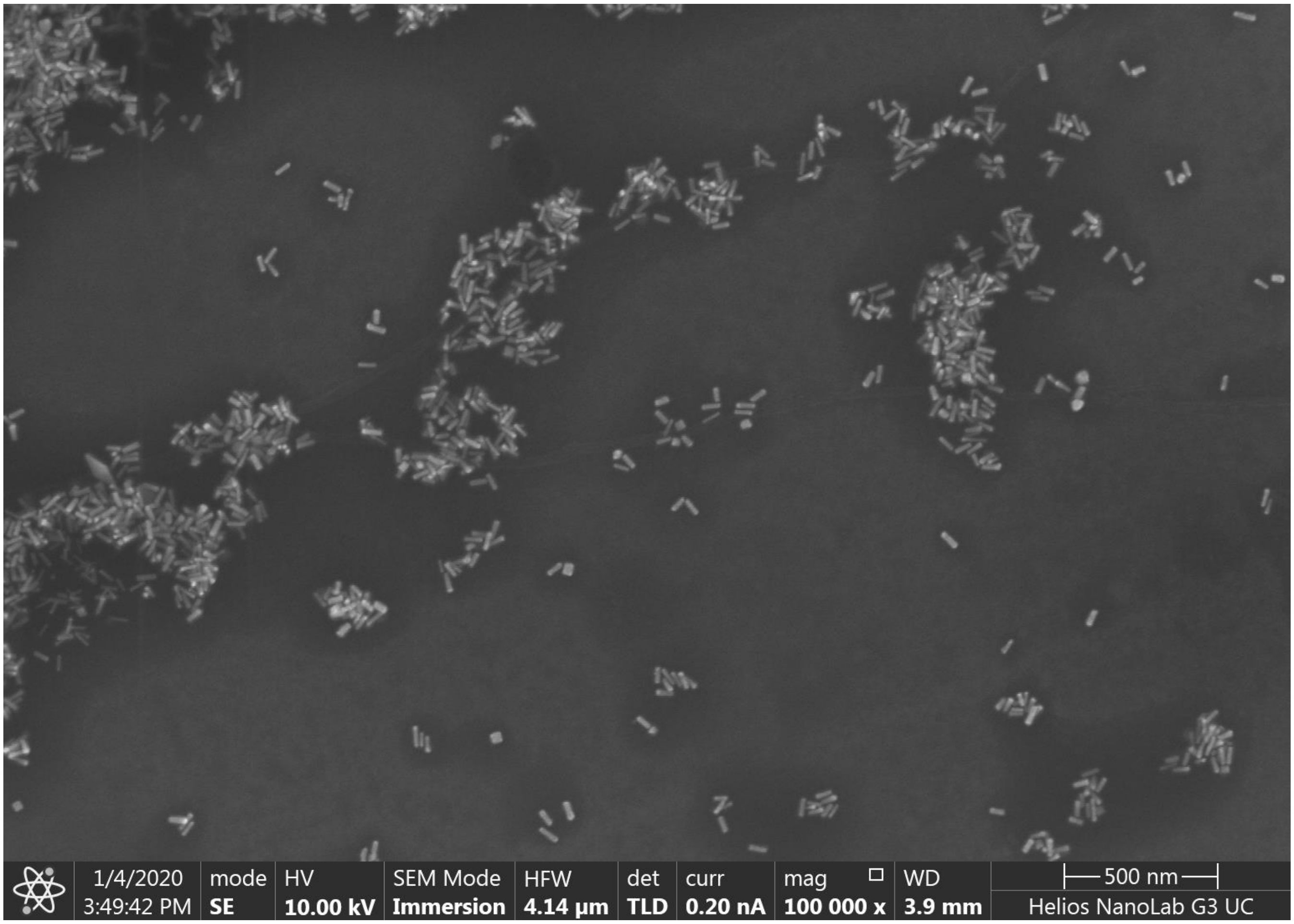

Figure S21. Large-area SEM image of GNR@Se hybrids prepared with PDDA in the presence of 0.56 mM SeO_2.

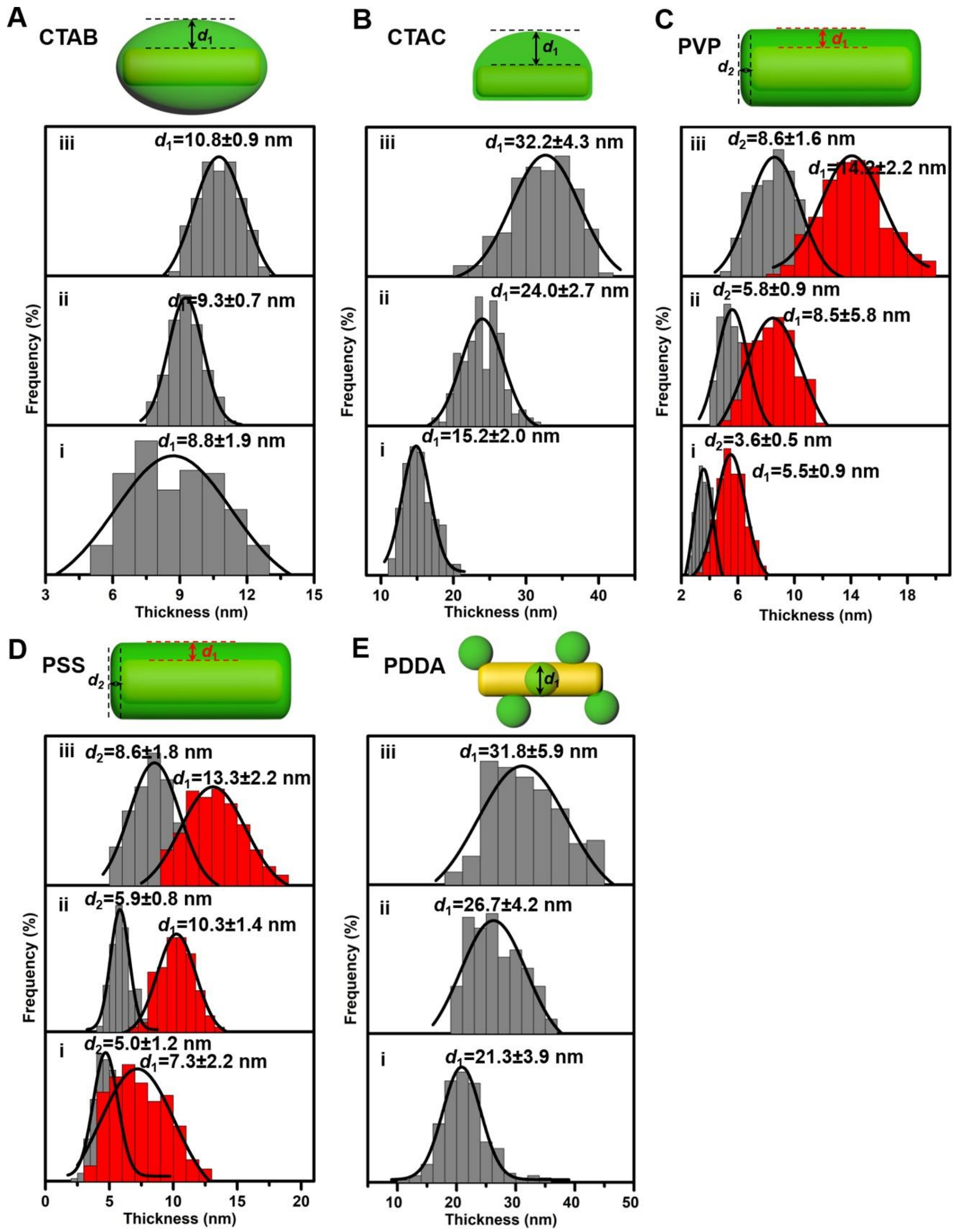

Figure S22. Size distributions of GNR@$Cu_{2-x}Se$ heterostructures prepared with various capping agents in the presence of SeO_2 concentrations. (A-E) Size distributions of GNR@$Cu_{2-x}Se$ heterostructures prepared with (A) CTAB, (B) CTAC, (C) PVP, (D) PSS and (E) PDDA in the presence of (i) 0.28, (ii) 0.56 and (iii) 1.12 mM SeO_2.

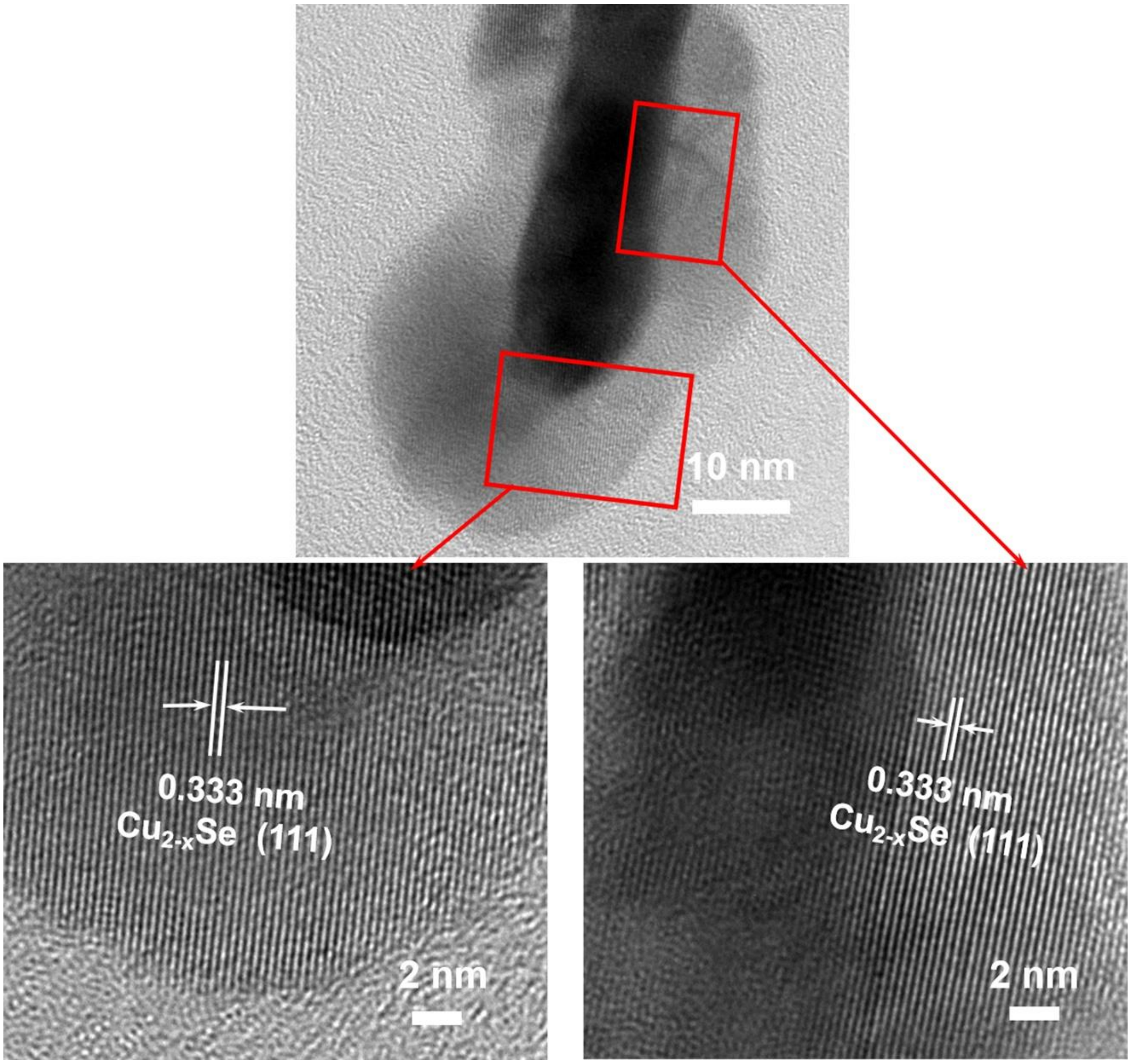

Figure S23. Representative HRTEM image of the $Cu_{2-x}Se$ domain in GNR@$Cu_{2-x}Se$ hybrid heterostructures prepared with PVP. The lattice fringe with the interplanar spacing of 0.333 nm corresponds to the (111) plane of the cubic berzelianite phase structure of $Cu_{2-x}Se$, verifying the cubic berzelianite crystal structure consistent with the results from XRD measurements.

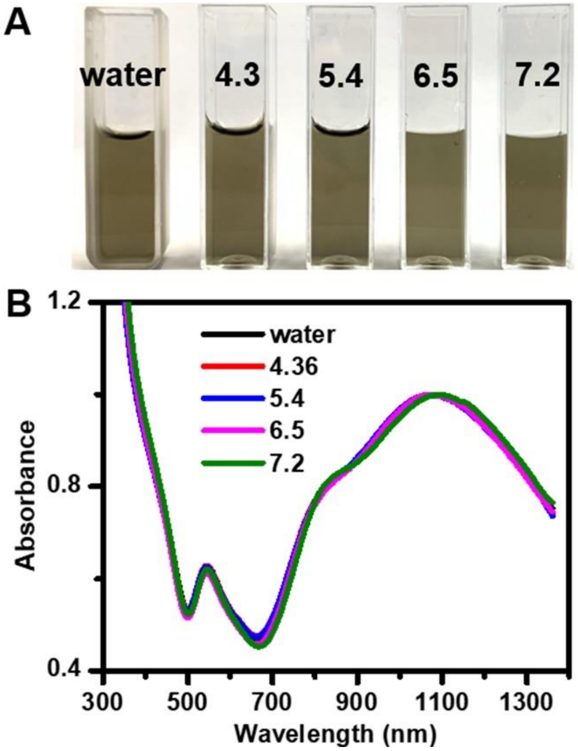

Figure S24. Stability of GNR@$Cu_{2-x}Se$ hybrid heterostructures in various pH values. (A) Optical photographs and (B) optical absorption spectra of GNR@$Cu_{2-x}Se$ hybrids prepared with PVP in suspensions with pH=4.3, 5.4, 6.5 and 7.2. Results clearly confirm the excellent stability of GNR@$Cu_{2-x}Se$ hybrids in a wide pH range from the tumor microenvironment to heathy physiological conditions.

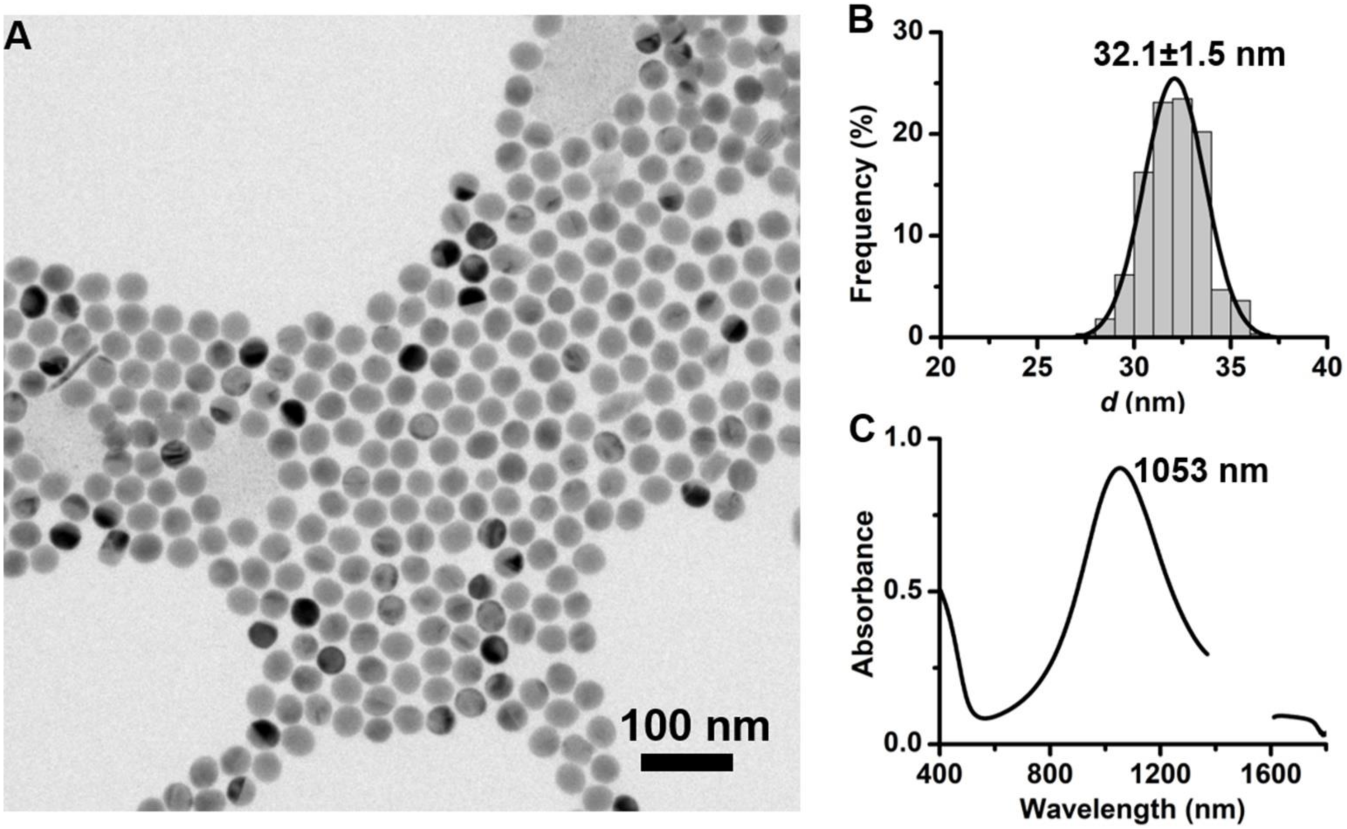

Figure S25. Structure and optical properties of $Cu_{2-x}Se$ NCs. (A) TEM image and (B) size distribution of $Cu_{2-x}Se$ NCs, and (C) optical absorption spectrum of colloidal suspension of $Cu_{2-x}Se$ NCs in water.

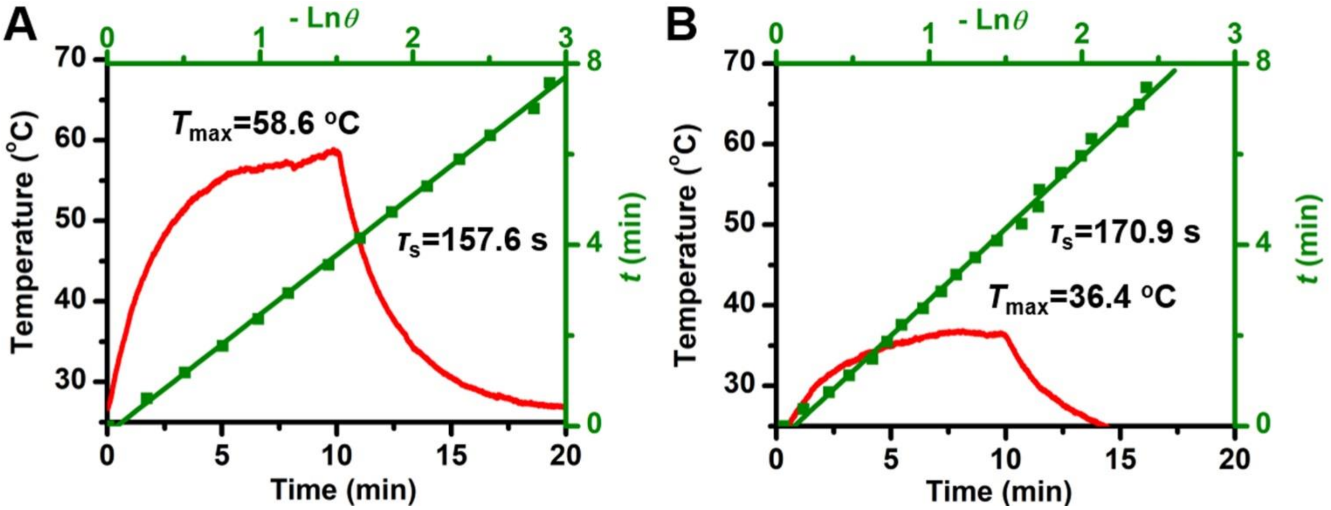

Figure S26. Photothermal effects of GNRs at a power density of 1.0 W/cm^2 under the 808 nm and 1064 nm-laser, respectively. (A, B) Heating-cooling curves and plots of t vs -lnθ taken from the photothermal heating-cooling curves during the cooling period after the laser is switched off (A) under the 808 nm-laser and (B) under the 1064 nm-laser, of aqueous suspensions of GNRs.

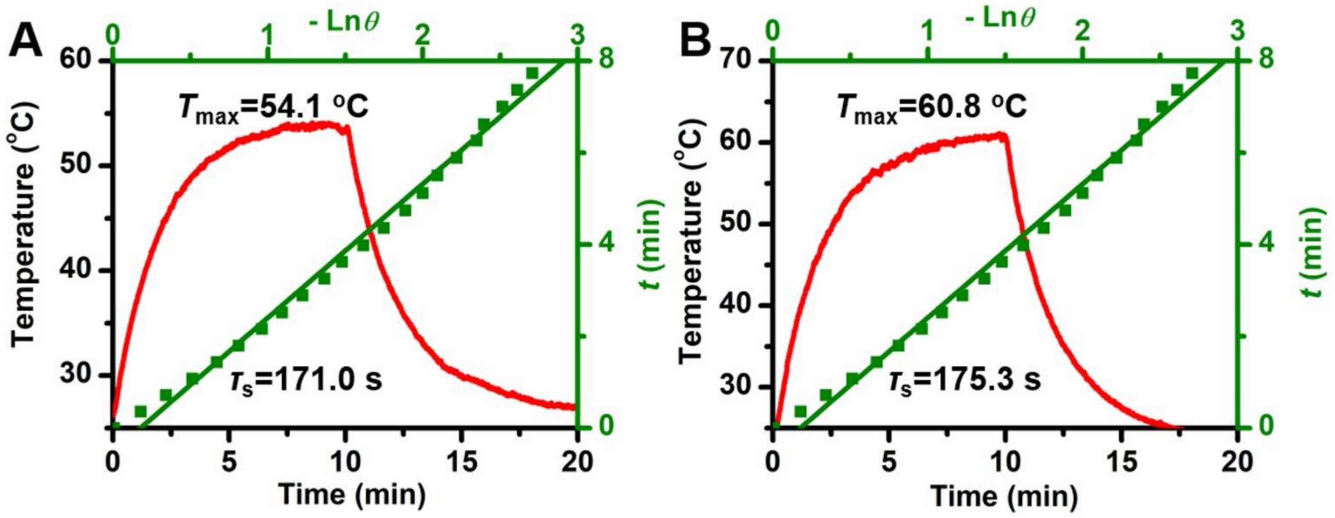

Figure S27. Photothermal effects of $Cu_{2-x}Se$ NCs at a power density of 1.0 W/cm^2 under the 808 nm and 1064 nm-laser, respectively. (A, B) Heating-cooling curves and plots of t vs -lnθ taken from the photothermal heating-cooling curves during the cooling period after the laser is switched off (A) under the 808 nm-laser and (B) under the 1064 nm-laser, of aqueous suspensions of $Cu_{2-x}Se$ NCs.

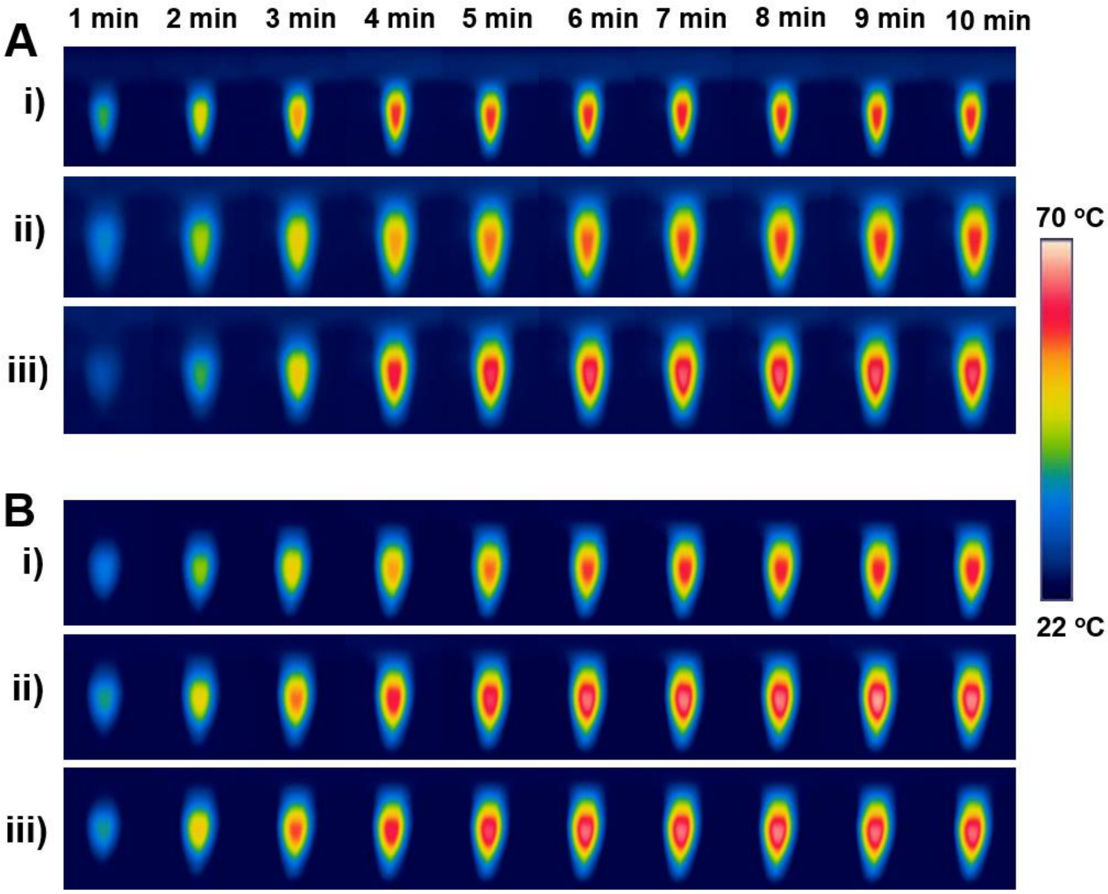

Figure S28. Infrared thermal images under the (A) 808 nm-laser and (B) 1064 nm-laser of aqueous suspensions of GNR@$Cu_{2-x}Se$ heterostructures prepared with CTAB in the presence of (i) 0.28 mM, (ii) 0.56 mM and (iii) 1.12 mM SeO_2 at a power density of 1.0 W/cm^2, respectively.

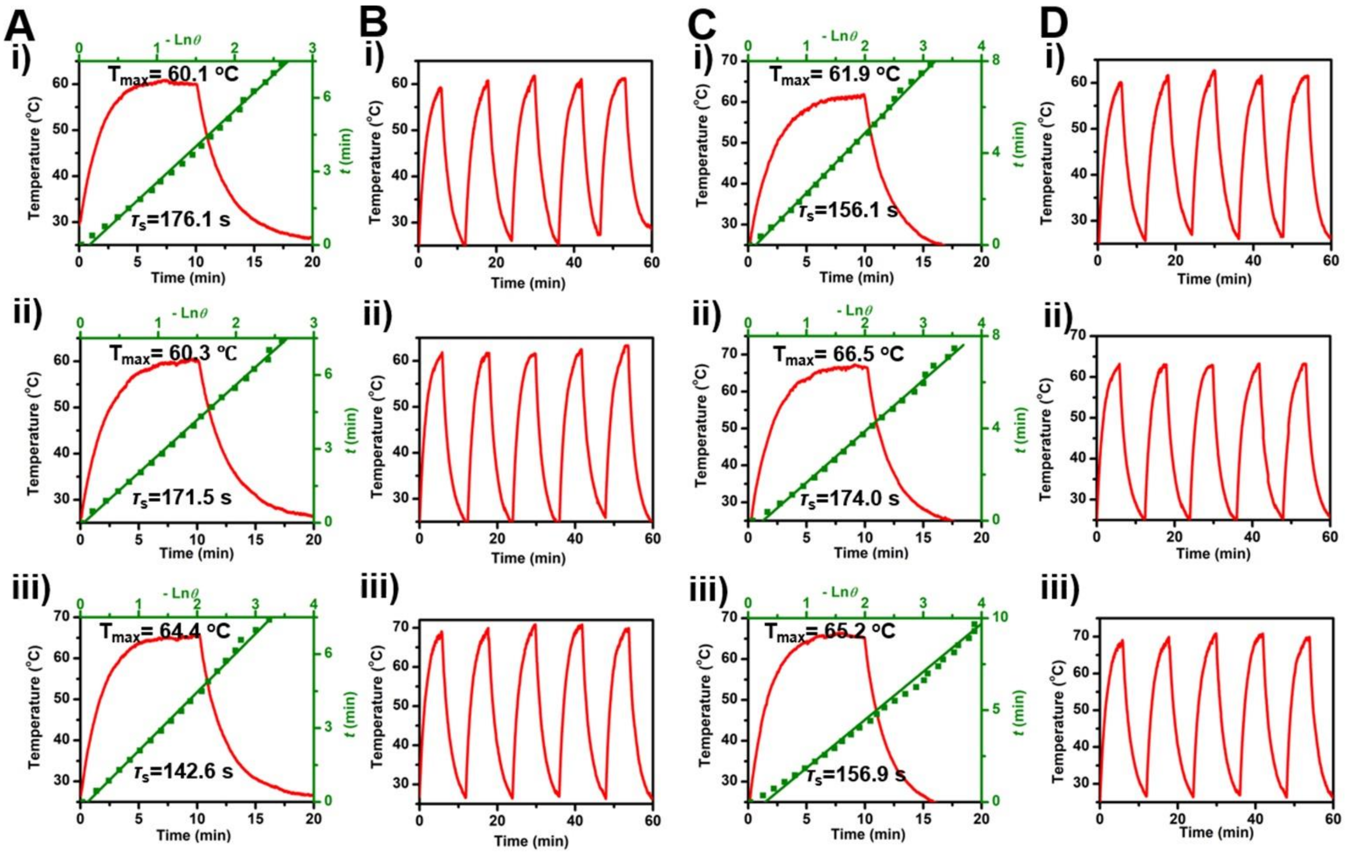

Figure S29. Photothermal effects of GNR@$Cu_{2-x}Se$ heterostructures prepared with CTAB at a power density of 1.0 W/cm^2 under the 808 nm and 1064 nm-laser, respectively. (A, C) Heating-cooling curves and plots of *t* vs -lnθ taken from the photothermal heating-cooling curves during the cooling period after the laser is switched off (A) under the 808 nm-laser and (C) under the 1064 nm-laser, of aqueous suspensions of GNR@$Cu_{2-x}Se$ heterostructures prepared with CTAB in the presence of (i) 0.28 mM, (ii) 0.56 mM and (iii) 1.12 mM SeO_2, respectively. (B, D) Photothermal heating-cooling curves for five laser on/off cycles (B) under the 808 nm-laser and (D) under the 1064 nm-laser of aqueous suspensions of GNR@$Cu_{2-x}Se$ heterostructures with CTAB in the presence of (i) 0.28 mM, (ii) 0.56 mM and (iii) 1.12 mM SeO_2, respectively.

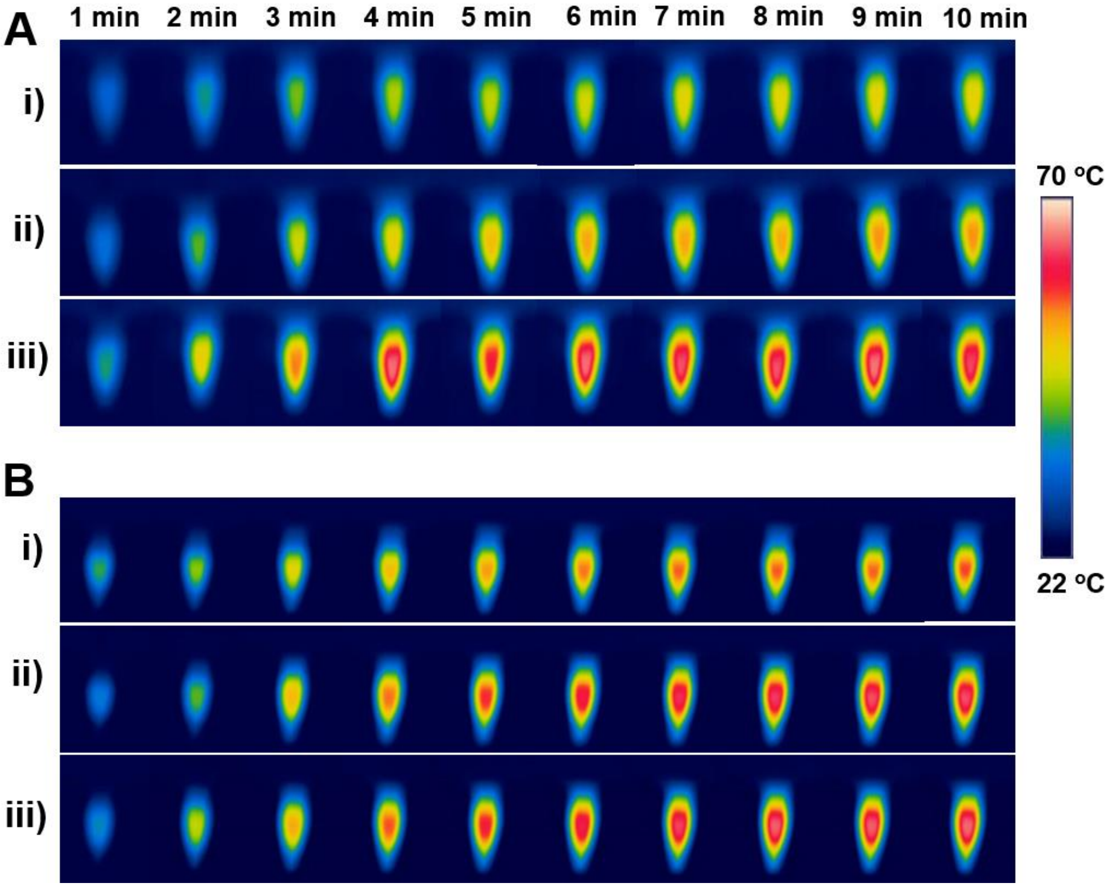

Figure S30. Infrared thermal images under the (A) 808 nm-laser and (B) 1064 nm-laser of aqueous suspensions of GNR@$Cu_{2-x}Se$ heterostructures prepared with CTAC in the presence of (i) 0.28 mM, (ii) 0.56 mM and (iii) 1.12 mM SeO_2 at a power density of 1.0 W/cm^2, respectively.

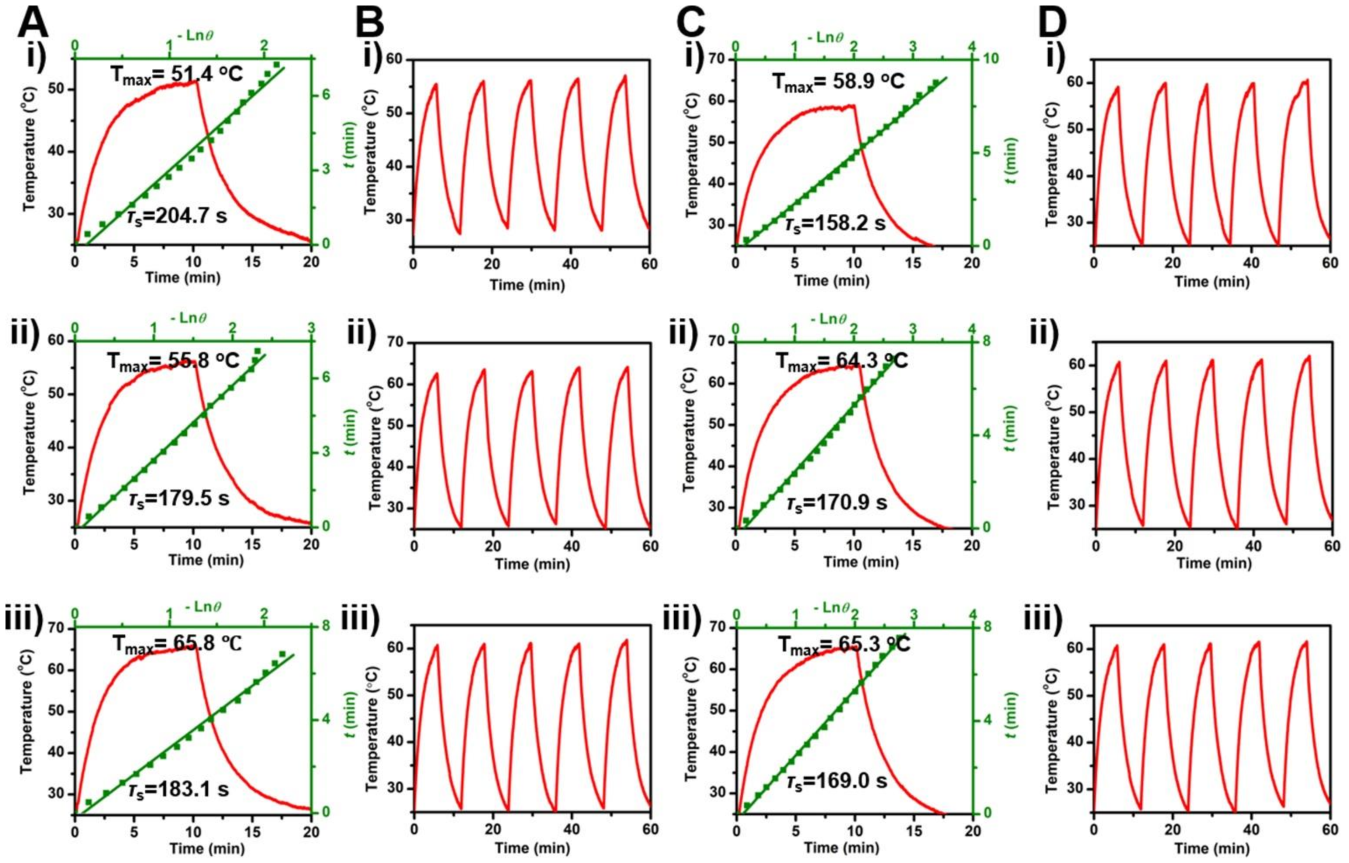

Figure S31. Photothermal effects of GNR@Cu_{2-x}Se heterostructures prepared with CTAC at a power density of 1.0 W/cm^2 under the 808 nm and 1064 nm-laser, respectively. (A, C) Heating-cooling curves and plots of *t* vs -ln*θ* taken from the photothermal heating-cooling curves during the cooling period after the laser is switched off (A) under the 808 nm-laser and (C) under the 1064 nm-laser, of aqueous suspensions of GNR@Cu_{2-x}Se heterostructures prepared with CTAC in the presence of (i) 0.28 mM, (ii) 0.56 mM and (iii) 1.12 mM SeO_2, respectively. (B, D) Photothermal heating-cooling curves for five laser on/off cycles (B) under the 808 nm-laser and (D) under the 1064 nm-laser of aqueous suspensions of GNR@Cu_{2-x}Se heterostructures with CTAC in the presence of (i) 0.28 mM, (ii) 0.56 mM and (iii) 1.12 mM SeO_2, respectively.

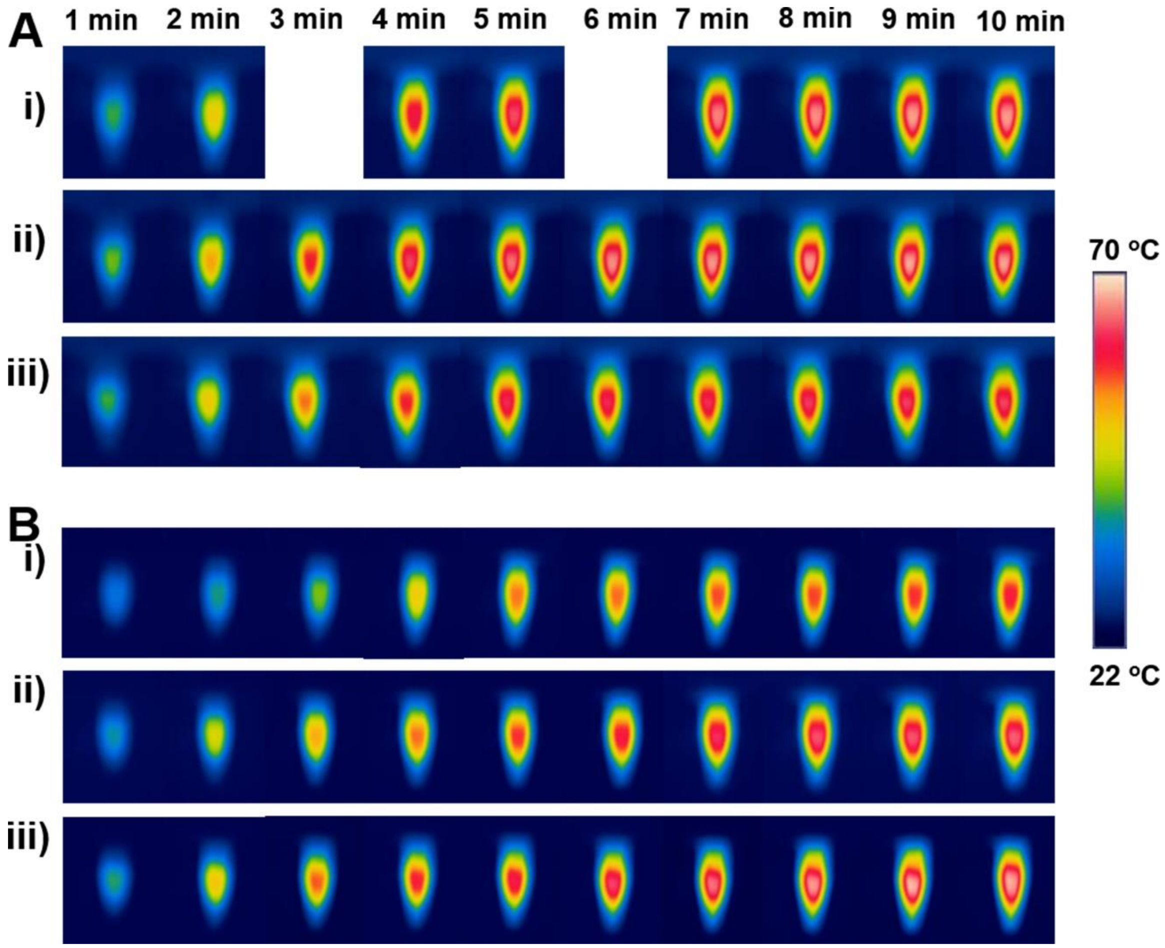

Figure S32. Infrared thermal images under the (A) 808 nm-laser and (B) 1064 nm-laser of aqueous suspensions of GNR@$Cu_{2-x}Se$ heterostructures prepared with PVP in the presence of (i) 0.28 mM, (ii) 0.56 mM and (iii) 1.12 mM SeO_2 at a power density of 1.0 W/cm^2, respectively.

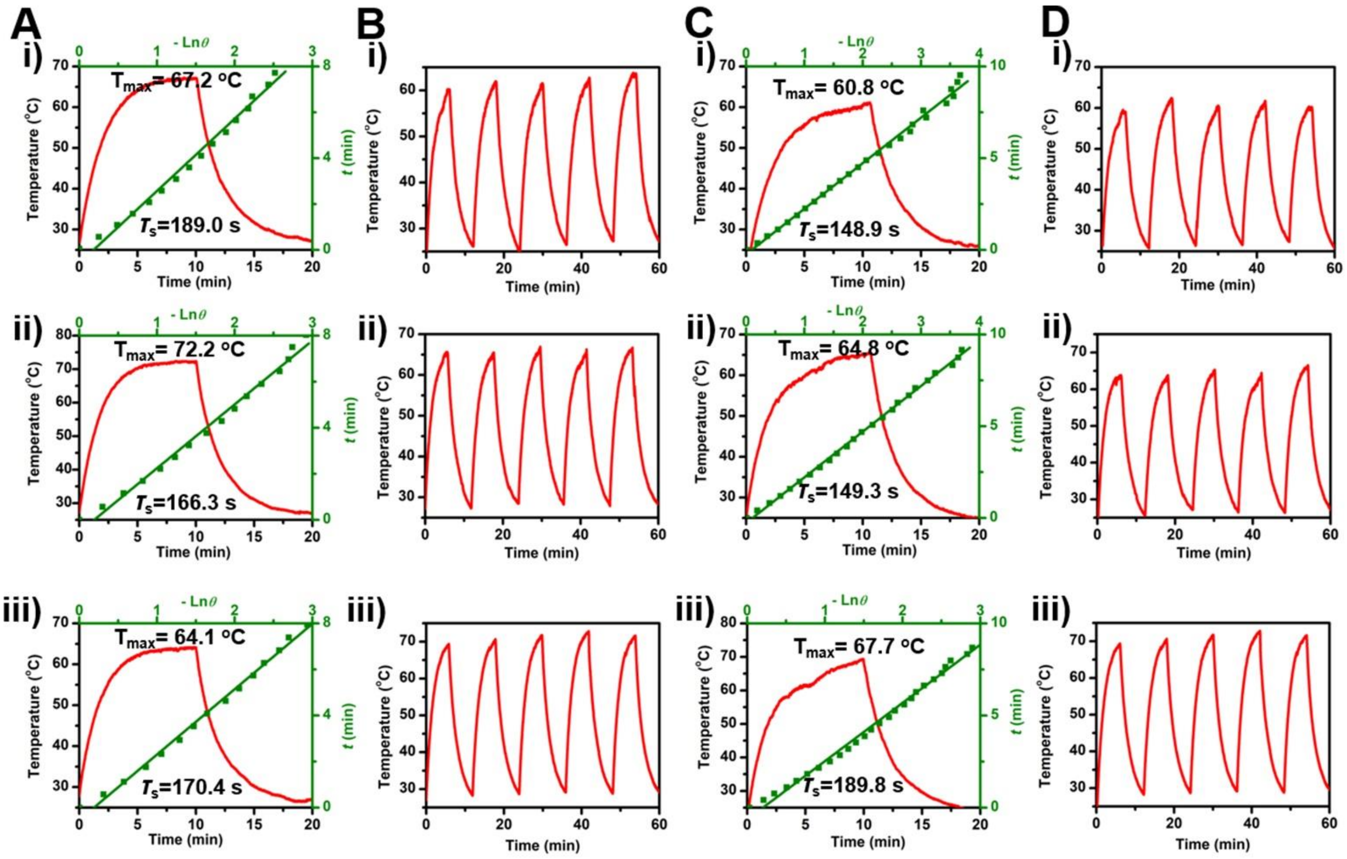

Figure S33. Photothermal effects of GNR@$Cu_{2-x}Se$ heterostructures prepared with PVP at a power density of 1.0 W/cm^2 under the 808 nm and 1064 nm-laser, respectively. (A, C) Heating-cooling curves and plots of *t* vs -lnθ taken from the photothermal heating-cooling curves during the cooling period after the laser is switched off (A) under the 808 nm-laser and (C) under the 1064 nm-laser, of aqueous suspensions of GNR@$Cu_{2-x}Se$ heterostructures prepared with PVP in the presence of (i) 0.28 mM, (ii) 0.56 mM and (iii) 1.12 mM SeO_2, respectively. (B, D) Photothermal heating-cooling curves for five laser on/off cycles (B) under the 808 nm-laser and (D) under the 1064 nm-laser of aqueous suspensions of GNR@$Cu_{2-x}Se$ heterostructures with PVP in the presence of (i) 0.28 mM, (ii) 0.56 mM and (iii) 1.12 mM SeO_2, respectively.

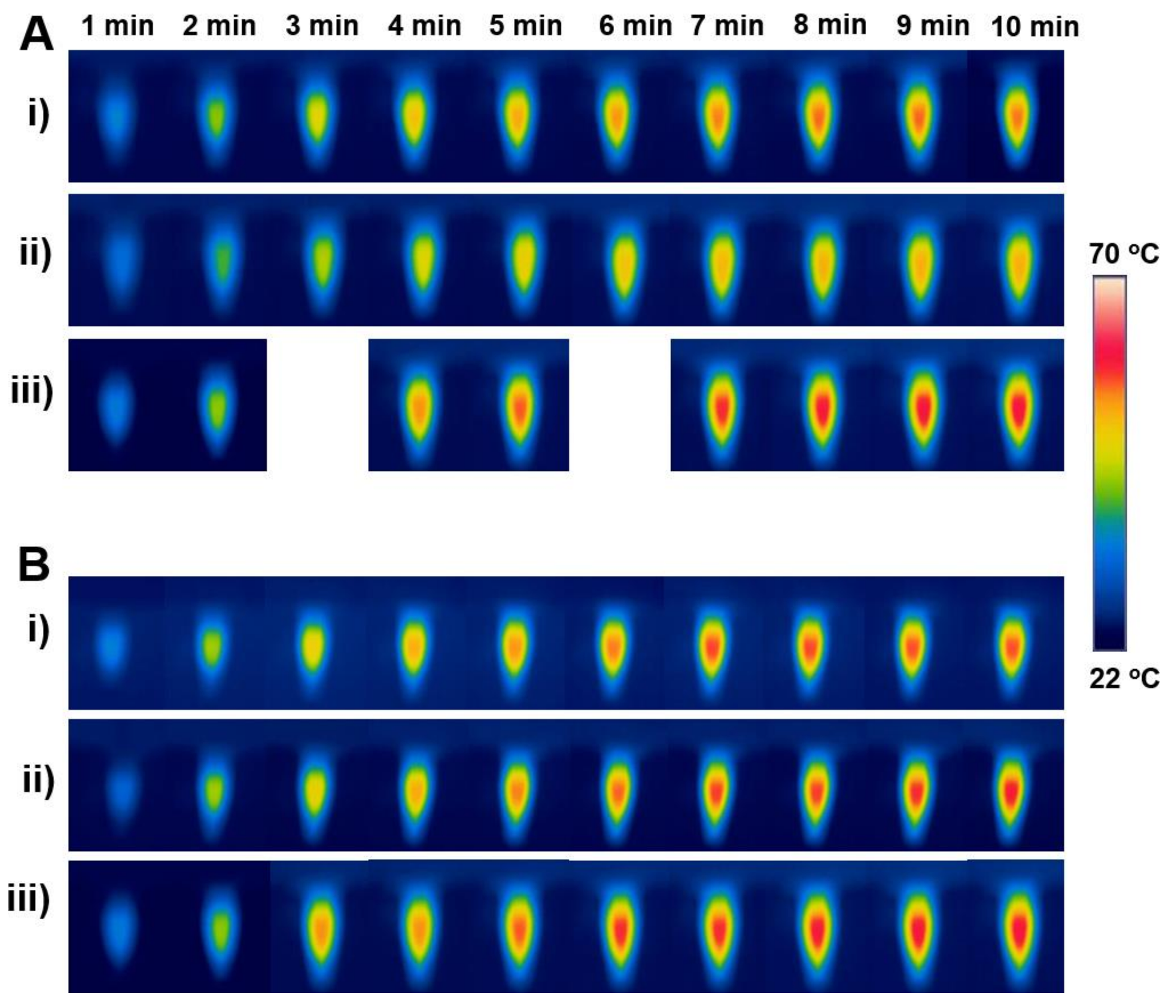

Figure S34. Infrared thermal images under the (A) 808 nm-laser and (B) 1064 nm-laser of aqueous suspensions of GNR@$Cu_{2-x}Se$ heterostructures prepared with PSS in the presence of (i) 0.28 mM, (ii) 0.56 mM and (iii) 1.12 mM SeO_2 at the power density of 1.0 W/cm^2, respectively.

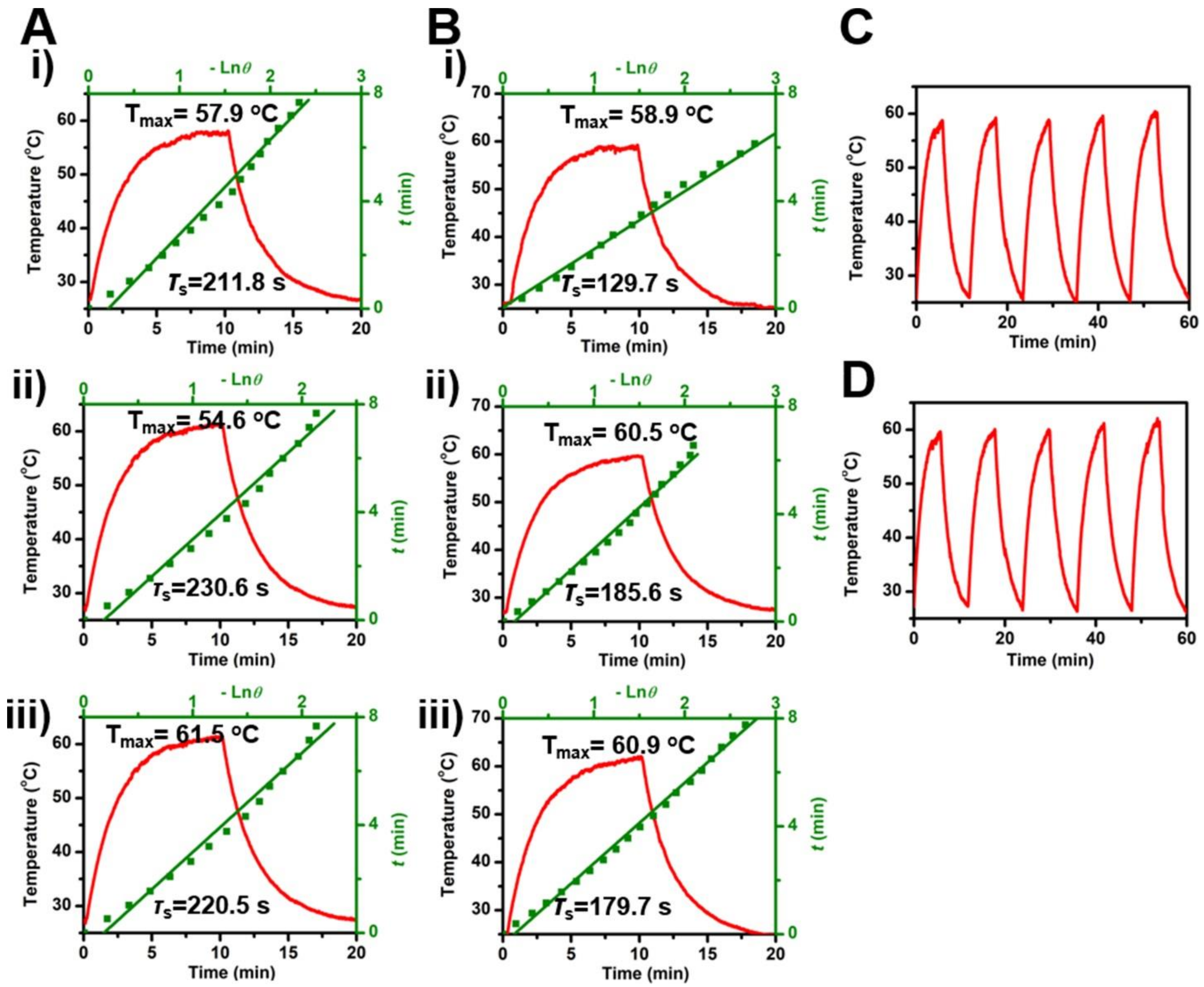

Figure S35. Photothermal effects of GNR@$Cu_{2-x}Se$ heterostructures prepared with PSS at a power density of 1.0 W/cm^2 under the 808 nm and 1064 nm-laser, respectively. (A, B) Heating-cooling curves and plots of *t* vs -lnθ taken from the photothermal heating-cooling curves during the cooling period after the laser is switched off (A) under the 808 nm-laser and (B) under the 1064 nm-laser, of aqueous suspensions of GNR@$Cu_{2-x}Se$ heterostructures prepared with PSS in the presence of (i) 0.28 mM, (ii) 0.56 mM and (iii) 1.12 mM SeO_2, respectively. (C, D) Photothermal heating-cooling curves for five laser on/off cycles (C) under the 808 nm-laser and (D) under the 1064 nm-laser of aqueous suspensions of GNR@$Cu_{2-x}Se$ heterostructures with PSS in the presence of 0.28 mM SeO_2.

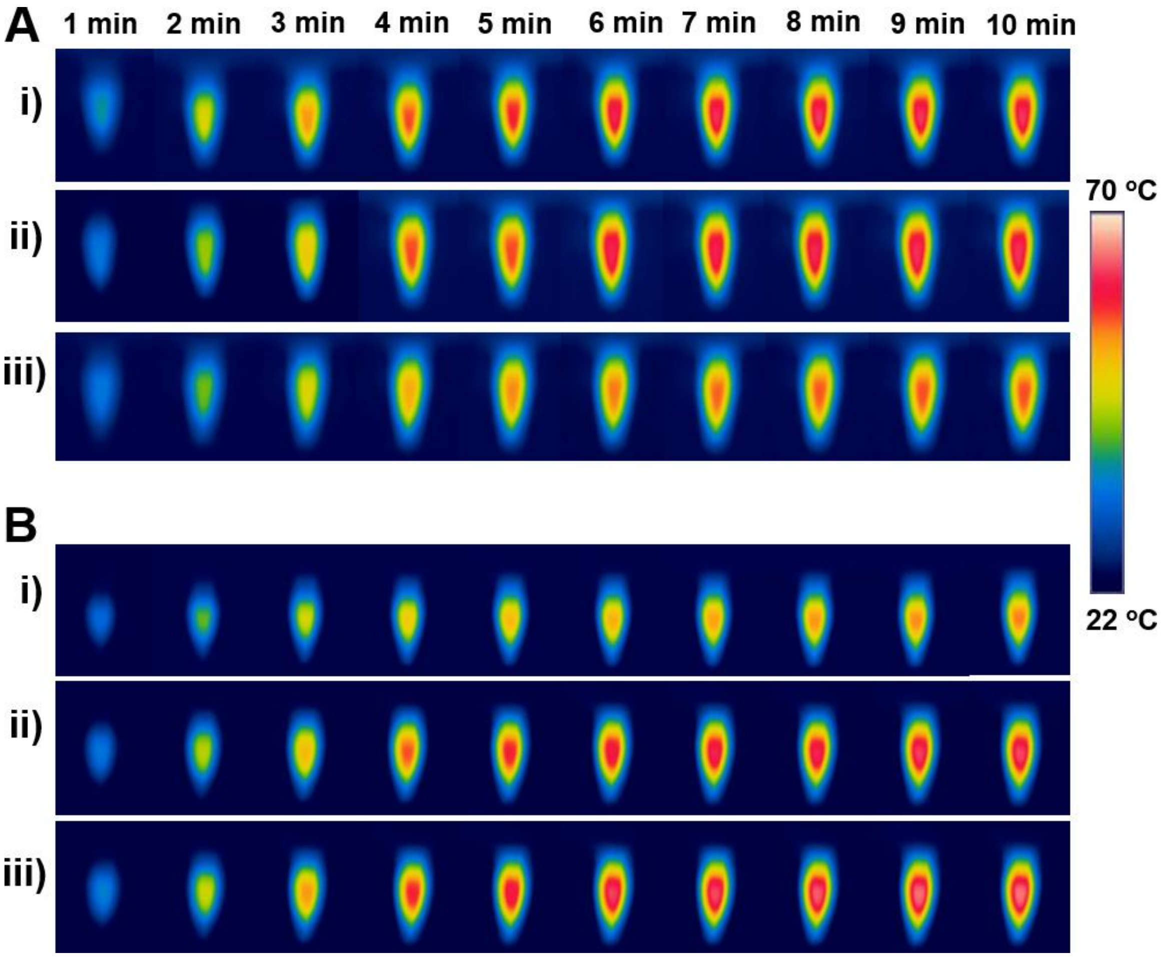

Figure S36. Infrared thermal images under the (A) 808 nm-laser and (B) 1064 nm-laser of aqueous suspensions of GNR@Cu_{2-x}Se heterostructures prepared with PDDA in the presence of (i) 0.28 mM, (ii) 0.56 mM and (iii) 1.12 mM SeO_2 at the power density of 1.0 W/cm^2, respectively.

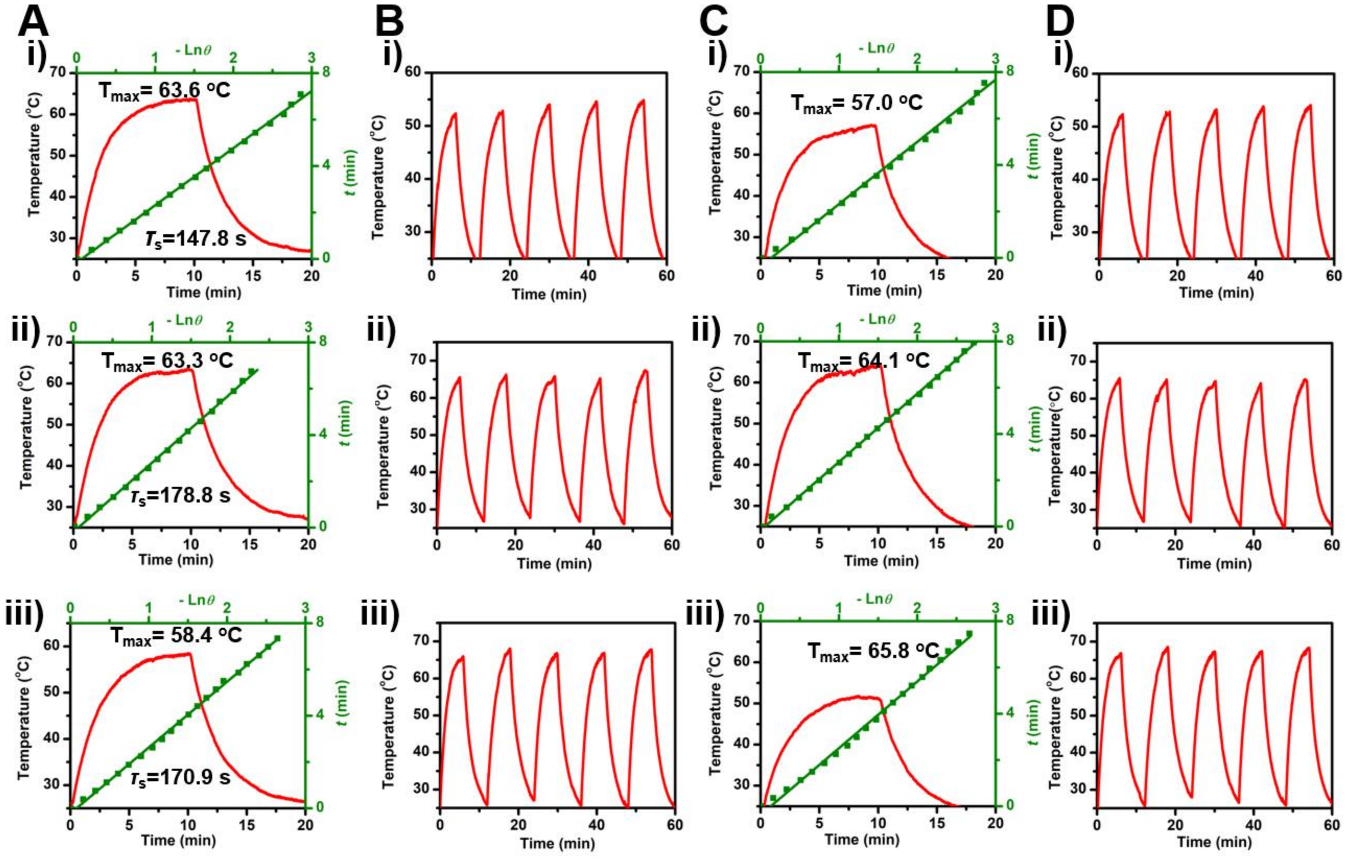

Figure S37. Photothermal effects of GNR@$Cu_{2-x}Se$ heterostructures prepared with PDDA at a power density of 1.0 W/cm^2 under the 808 nm and 1064 nm-laser, respectively. (A, C) Heating-cooling curves and plots of t vs -lnθ taken from the photothermal heating-cooling curves during the cooling period after the laser is switched off (A) under the 808 nm-laser and (C) under the 1064 nm-laser, of aqueous suspensions of GNR@$Cu_{2-x}Se$ heterostructures prepared with PDDA in the presence of (i) 0.28 mM, (ii) 0.56 mM and (iii) 1.12 mM SeO_2, respectively. (B, D) Photothermal heating-cooling curves for five laser on/off cycles (B) under the 808 nm-laser and (D) under the 1064 nm-laser of aqueous suspensions of GNR@$Cu_{2-x}Se$ heterostructures with PDDA in the presence of (i) 0.28 mM, (ii) 0.56 mM and (iii) 1.12 mM SeO_2, respectively.

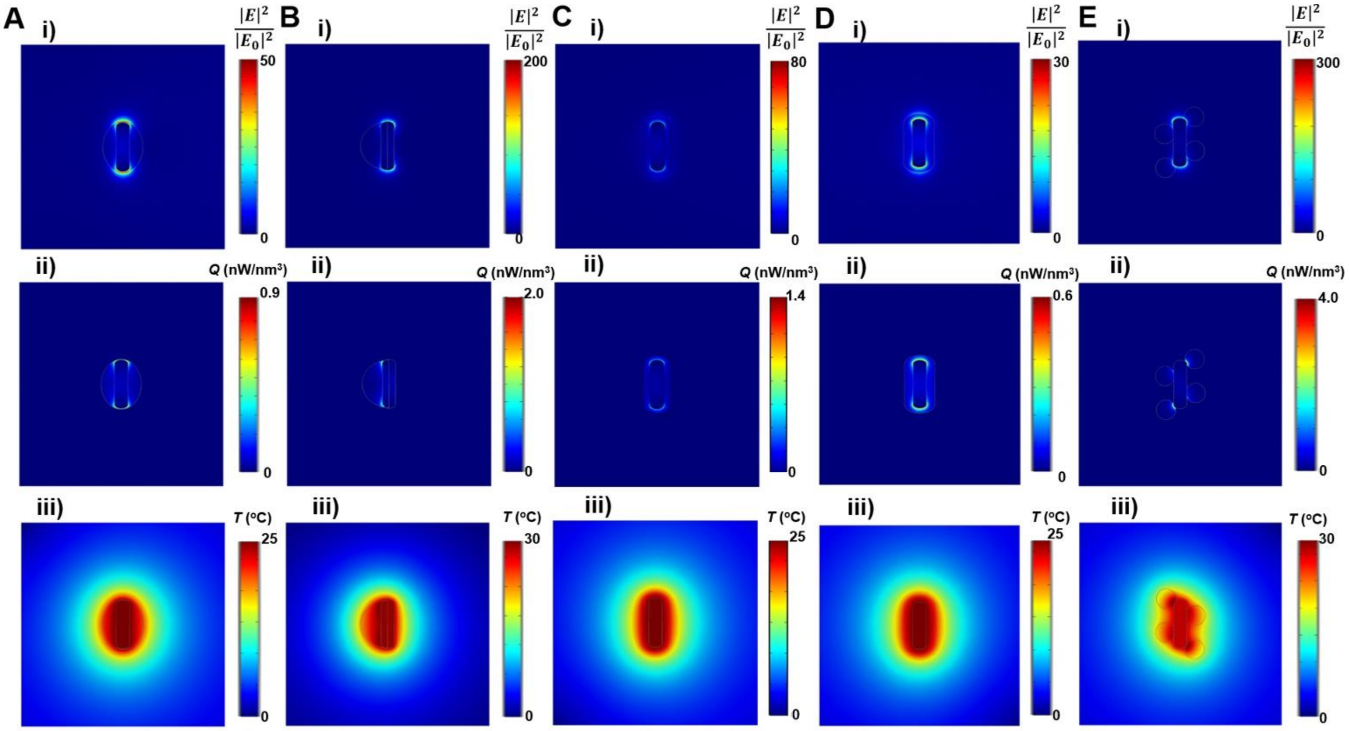

Figure S38. Theoretical analysis of photothermal effects under the 808 nm-laser of a GNR@$Cu_{2-x}Se$ heterostructure in water prepared using various capping agents. (A-E) distributions of (i) calculated electric field (($|\mathbf{E}|^2/|\mathbf{E}_0|^2$) intensity at 808 nm, (ii) heat power volume density (Q, nW/nm^3) and (iii) steady-state temperature (T, °C) under the 808 nm-laser of a GNR@$Cu_{2-x}Se$ heterostructures. The models and set-up parameters for simulations are the same to that shown in Figure 5.

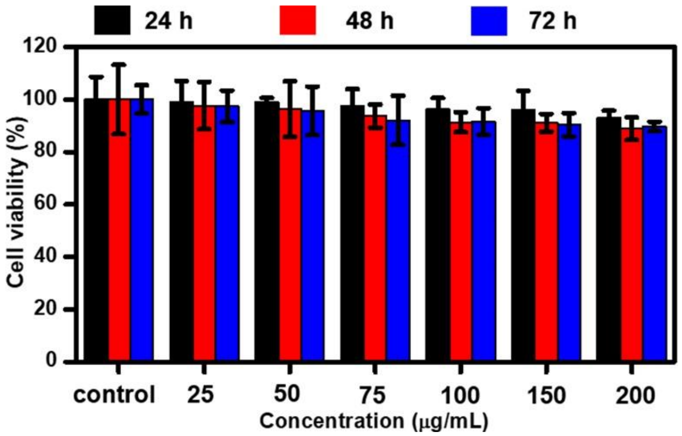

Figure S39. Cell viability of human normal liver cell line L-02 cells after being incubated for 24, 48 and 72 h in culture medium containing GNR@$Cu_{2-x}Se$ hybrids at various concentrations (0-200 μg/mL for gold). GNR@$Cu_{2-x}Se$ hybrids prepared with PVP at 1.12 mM SeO_2 were used.

S2. *In vitro* hemolysis assay

Hemolysis test was performed to evaluate the hemocompatibility of PEG-GNR@$Cu_{2-x}Se$ hybrids. Typically, 1 mL of mice red blood cell (RBC) solution was obtained after anticoagulation and washing three times with PBS solution, and then dispersed in 4 mL of PBS. After that, 0.2 mL of RBCs obtained above was mixed with 0.8 mL of GNR@$Cu_{2-x}Se$ in PBS (25, 50,75, 100, 200, 400 μg/mL). Ultrapure water and PBS were used as the positive control and negative control, respectively. Then the samples were shaken and kept stable at 37 °C for 4 h, the mixtures were centrifuged at 10000 rpm for 10 min. Optical photographs were taken, and hemoglobin in the supernatant was estimated on the basis of the absorbance at 545 nm. The hemolysis ratio was calculated using the following equation:

$$\text{hemolysis percentage (\%)} = \frac{A_{sample} - A_{negative}}{A_{positive} - A_{negative}} \times 100$$

where A_{sample}, A_{negative} and A_{positive} are the absorbance of the samples, negative control and positive control, respectively.

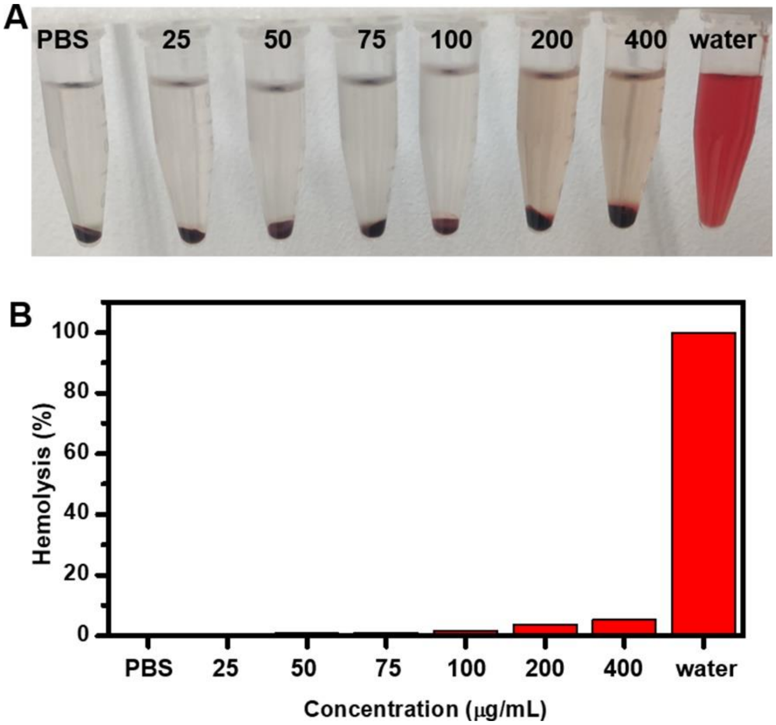

Figure S40. Hemolytic effect of red blood cells induced by GNR@$Cu_{2-x}Se$ hybrids. (A) Optical photographs and (B) hemolysis percentage after incubation for 24 h in suspensions of GNR@$Cu_{2-x}Se$ hybrids prepared with PVP and 1.12 mM SeO_2 at various concentrations (25, 50, 75, 100, 200 and 400 μg/mL). PBS and water were used as negative control and positive control, respectively. These results clearly show no hemolysis induced by GNR@$Cu_{2-x}Se$ hybrids.

S3. Test of blood biochemistry

The analysis of blood biochemistry was performed with three groups of healthy mice (n=3). Blood was collected directly from either healthy mice injected with 50 μL PBS or at 24 h- and 48 h-postinjection from heathy mice intravenously administrated with PEGylated GNR@$Cu_{2-x}Se$ solution (50 μL, 200 μg/mL for gold). Levels of different makers including alanine transaminase (ALT), aspartate transaminase (AST), alkaline phosphatase (ALP), blood urea nitrogen (BUN), globulin (GLB) and creatinine (CREA) were tested to evaluate the hepatic and kidney functions.

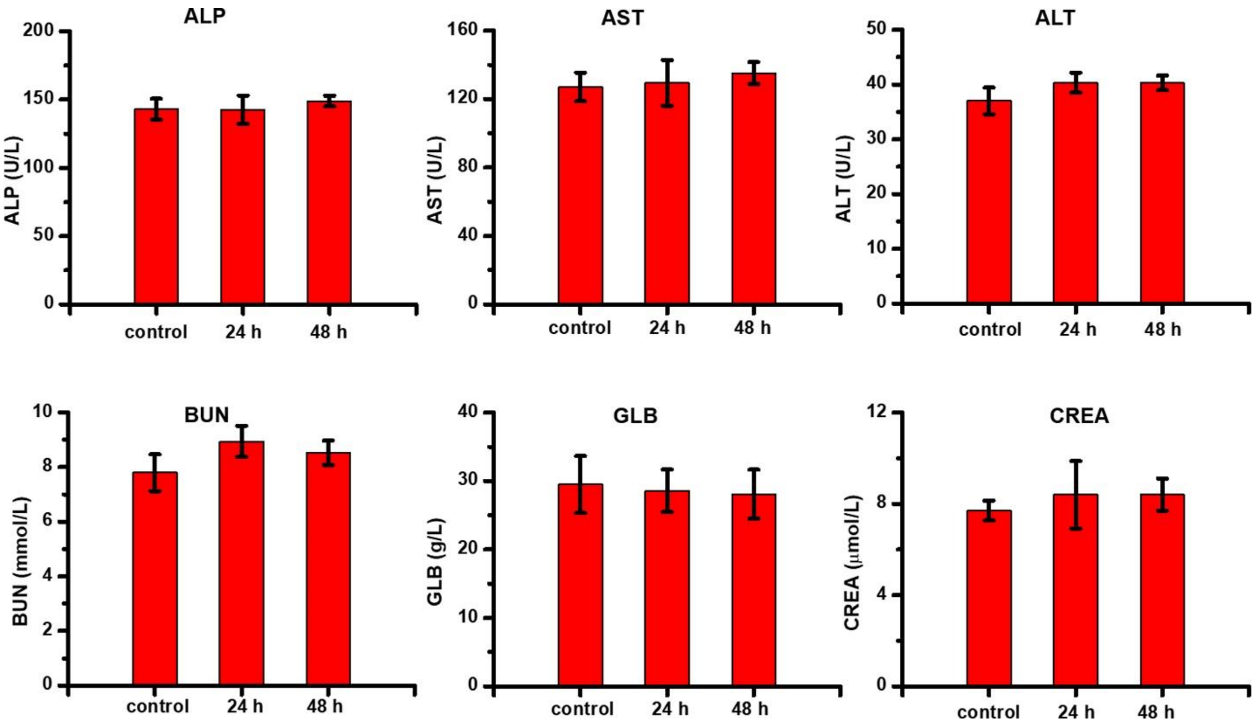

Figure S41. Analysis of blood biochemistry of heathy mice intravenously administrated with GNR@$Cu_{2-x}Se$ hybrids. ALP, AST, ALT, BUN, GLB and CREA levels from heathy mice after administration with GNR@$Cu_{2-x}Se$ hybrids at 24 h- and 48 h-post-injections are compared with the control mice. No significant difference for all these levels was observed, indicating no hepatic and kidney toxicity by GNR@$Cu_{2-x}Se$ hybrids prepared with PVP and 1.12 mM SeO_2.

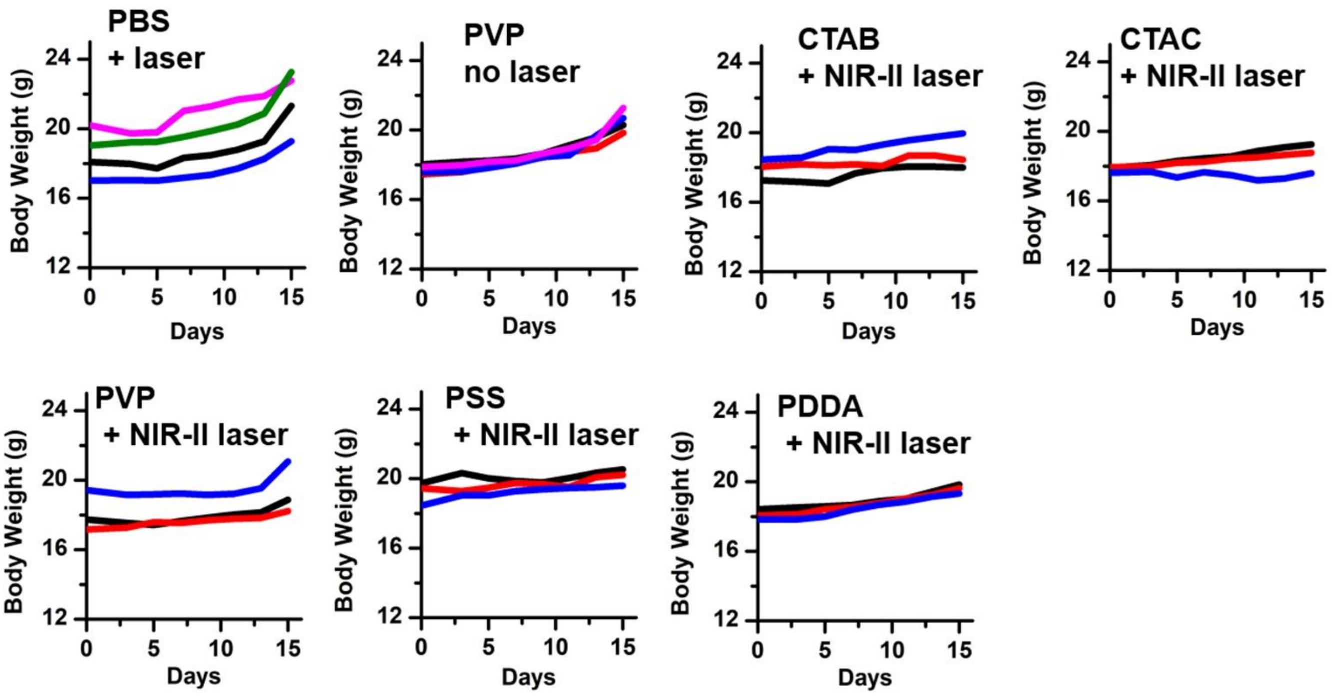

Figure S42. Individual body weight change curves of breast tumor-bearing mice shown in Figure 7C, with various treatments.

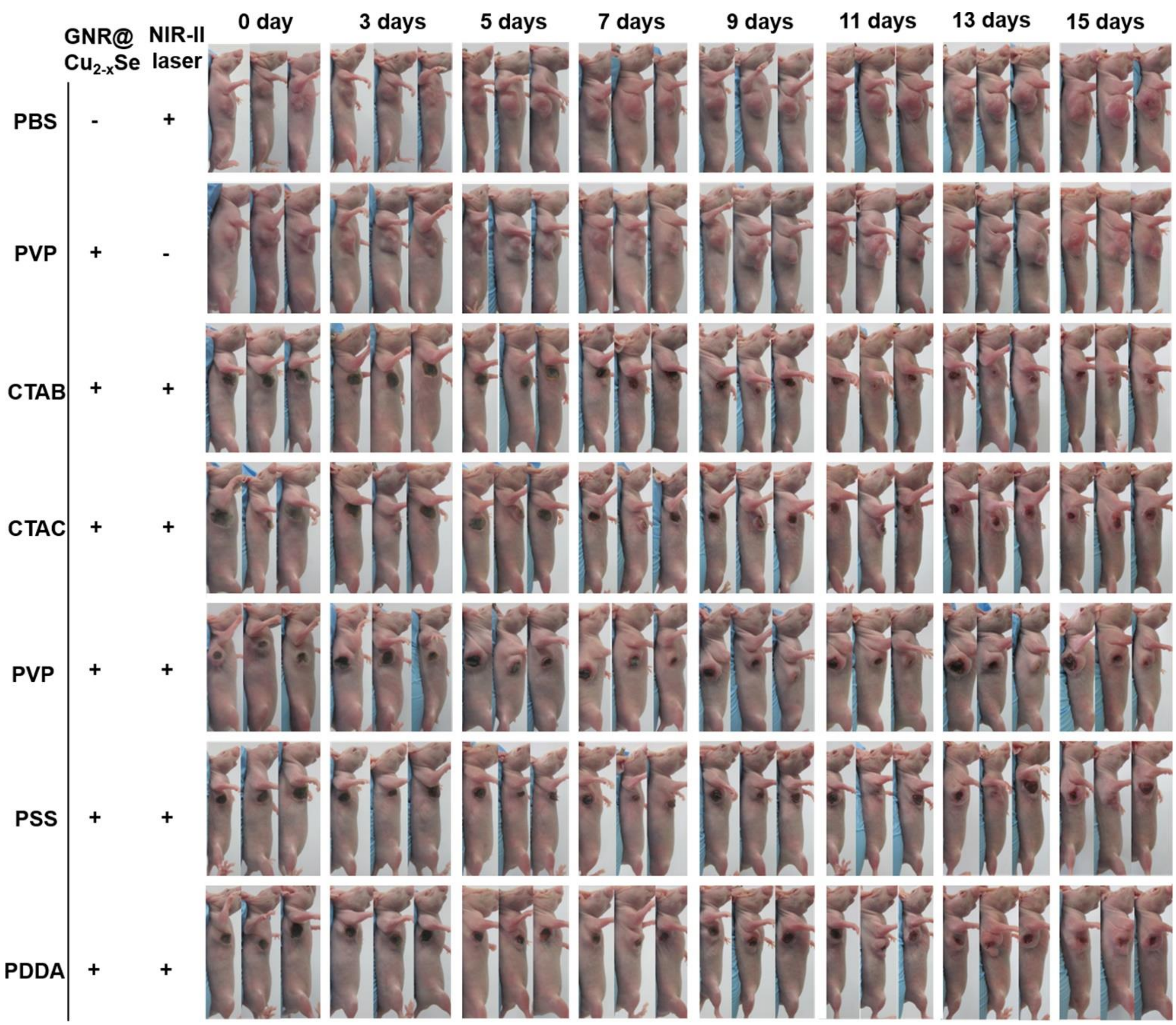

Figure S43. Photographs of breast tumor-bearing mice following various treatments including PBS + NIR-II laser, GNR@Cu_{2-x}Se heterostructures only, and GNR@Cu_{2-x}Se heterostructures prepared with various capping agents + NIR-II laser, respectively.

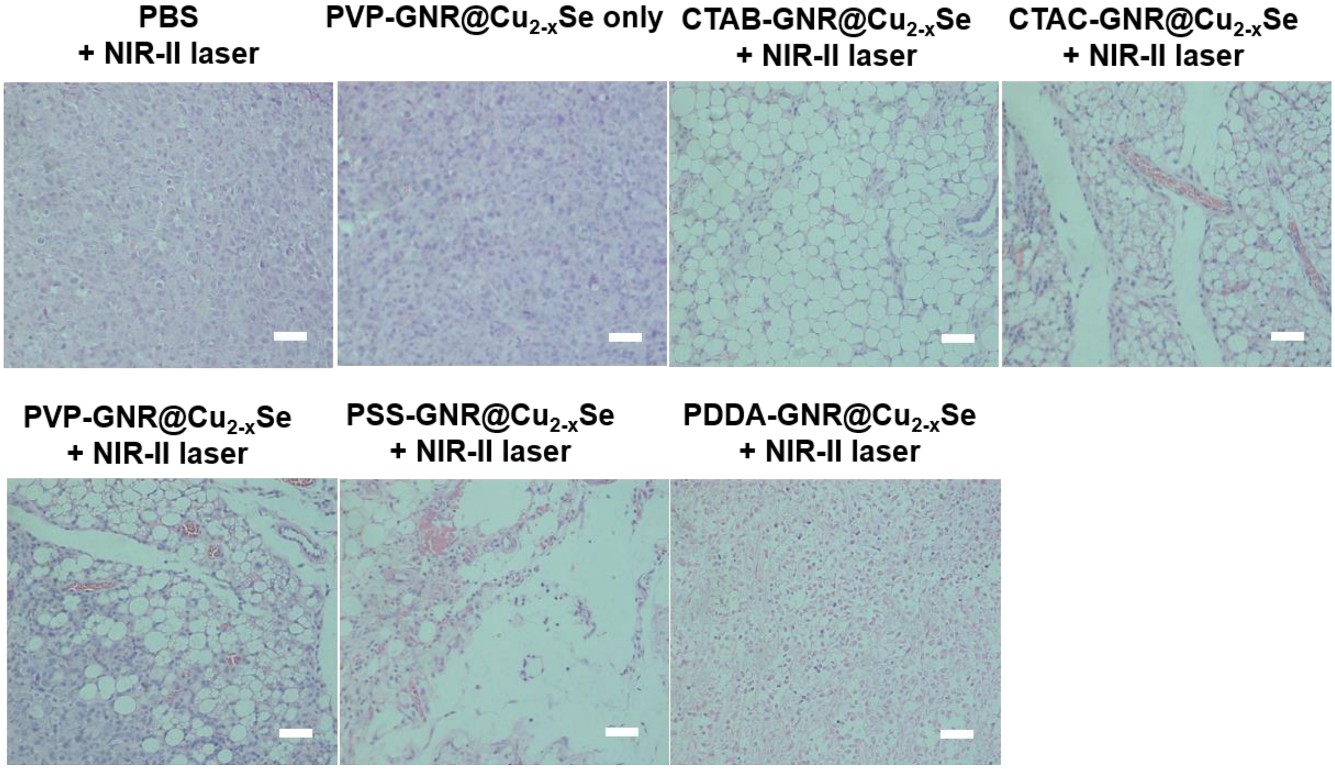

Figure S44. Representative H&E histological images of tumor tissues of tumor-bearing mice following various treatments with PBS + NIR-II laser, GNR@$Cu_{2-x}Se$ heterostructures prepared with PVP, and GNR@$Cu_{2-x}Se$ heterostructures (prepared with various capping agents) + NIR-II laser. Scale bar: 50 μm.
